# Revision of the genus-group *Hystricella* R. T. Lowe, 1855 from Porto Santo (Madeira Archipelago), with descriptions of new recent and fossil taxa (Gastropoda, Helicoidea, Geomitridae)

**DOI:** 10.3897/zookeys.732.21677

**Published:** 2018-01-24

**Authors:** Willy De Mattia, Marco T. Neiber, Klaus Groh

**Affiliations:** 1 Natural History Museum Vienna, Burgring 7, 1010 Vienna, Austria; 2 International Centre for Genetic Engineering and Biotechnology, Padriciano 99, 34149, Trieste, Italy; 3 Centre of Natural History, Zoological Museum, University of Hamburg, Martin-Luther-King-Platz 3, D-20146 Hamburg, Germany; 4 Hinterbergstraße 15, D-67098 Bad Dürkheim, Germany

**Keywords:** *Callina*, *Discula*, *Hystricella*, *Wollastonia* gen. n., Macaronesia, Porto Santo (Madeira Archipelago), recent, Quaternary, phylogeny

## Abstract

The genus-group *Hystricella* R. T. Lowe, 1855 is revised on the basis of conchological, anatomical and genetic characteristics. A new genus *Wollastonia* **gen. n.**, two recent species, *W. jessicae* **sp. n.** and *W. klausgrohi* **sp. n**., and one recent subspecies, *W. jessicae monticola* **ssp. n.** are described as new to science, as well as five fossil taxa, *H. microcarinata* **sp. n.**, *W. beckmanni* **sp. n.**, *W. falknerorum* **sp. n.**, *W. ripkeni* **sp. n.**, and *W. inexpectata* **sp. n.** For *Helix vermetiformis* R. T. Lowe, 1855, *H. leacockiana* Wollaston, 1878, *H. oxytropis* R. T. Lowe, 1831, *H. duplicata* R. T. Lowe, 1831 and *H. oxytropis var. ß subcarinulata* Wollaston, 1878 lectotypes are designated. For the taxa *Helix bicarinata* G. B. Sowerby I, 1824, *Helix bicarinata var. ß aucta* Wollaston, 1878 and *Discula bulverii* W. Wood, 1828 neotypes are selected. The taxa *aucta* and *subcarinulata* are elevated to specific rank. For the hitherto monospecific (sub-) genus *Callina* R. T. Lowe, 1855 it is shown that it is not closely related to the genus *Discula* but to the *Hystricella*-group and its generic rank is confirmed. The taxon *D. bulverii* W. Wood, 1828 is transferred from the genus *Discula* s. str. to the genus *Callina*. A further fossil taxon *C. waldeni* **sp. n.** is described as new to science.

## Abstract (de)

Die Gattungsgruppe *Hystricella* R. T. Lowe, 1855 wird anhand von conchologischen, anatomischen und genetischen Merkmalen revidiert. Eine neue Gattung *Wollastonia* **gen. n.**, zwei rezente Arten, *W. jessicae* **sp. n.** und *W. klausgrohi* **sp. n.** sowie eine rezente Unterart, *W. jessicae monticola* **ssp. n.**, werden neu beschrieben, wie auch fünf fossile Taxa, *H. microcarinata* **sp. n.**, *W. beckmanni* **sp. n.**, *W. falknerorum* **sp. n.**, *W. ripkeni* **sp. n.**, und *W. inexpectata* **sp. n.** Für *Helix vermetiformis* R. T. Lowe, 1855, *H. leacockiana* Wollaston, 1878, *H. oxytropis* R. T. Lowe, 1831, *H. duplicata* R. T. Lowe, 1831 und *H. oxytropis var. ß subcarinulata* Wollaston, 1878 werden Lectotypen designiert, für die Taxa *Helix bicarinata* G. B. Sowerby I, 1821, *Helix bicarinata var. ß aucta* Wollaston, 1878 und *Discula bulverii* W. Wood, 1828 Neotypen festgelegt. Die Taxa *aucta* und *subcarinulata* werden in einen spezifischen Rang erhoben. Es wird gezeigt, dass die bisher monospezifische (Unter-) Gattung *Callina* R. T. Lowe, 1855 nicht mit der Gattung *Discula*, sondern mit der *Hystricella*-Gruppe näher verwandt ist und im Gattungsrang bestätigt. Das Taxon *D. bulverii* W. Wood, 1828 wird aus der Gattung *Discula* s. str. zu *Callina* gestellt und ein weiteres fossiles Taxon *C. waldeni* **sp. n.** in dieser Gattung neu beschrieben.

## Abstract (es)

No presente trabalho o grupo genérico *Hystricella* R. T. Lowe, 1855 é revisto com base em critérios morfológicos, anatómicos e genéticos. É descrito um novo género, *Wollastonia* gen. n., duas novas espécies atuais, *W. jessicae* **sp. n.** e *W. klausgrohi* **sp. n.** e uma nova subespécie também atual, *W. jessicae monticola* **ssp. n.** São também descritas neste novo género cinco novas espécies fósseis: *H. microcarinata* **sp. n.**, *W. beckmanni* **sp. n.**, *W. falknerorum* **sp. n.**, *W. ripkeni* **sp. n.** e *H. inexpectata* **sp. n.** São designados lectótipos para as espécies, *Helix vermetiformis* R. T. Lowe, 1855, *H. leacockiana* Wollaston, 1878, *H. oxytropis* R. T. Lowe, 1831, *H. duplicata* R. T. Lowe, 1831 e *H. oxytropis var. ß subcarinulata* Wollaston, 1878. São designados neótipos para as taxones *Helix bicarinata* G. B. Sowerby I, 1824, *Helix bicarinata var. ß aucta* Wollaston, 1878 e *Discula bulverii* W. Wood, 1828. Os taxa *aucta* e *subcarinulata* são elevados à categoria de espécie. É demonstrado que o (sub) género, até agora monotípico, *Callina* R. T. Lowe, 1855, não está relacionado com o grupo genérico *Discula*, mas sim com o grupo genérico *Hystricella* e o seu estatuto de género é confirmado. O táxon *D. bulverii* W. Wood, 1828 é transferido do género *Discula* s. str. para o género *Callina* e é descrita uma nova espécie fóssil adicional, *C. waldeni* **sp. n.**

## Introduction

**Figure 1.**
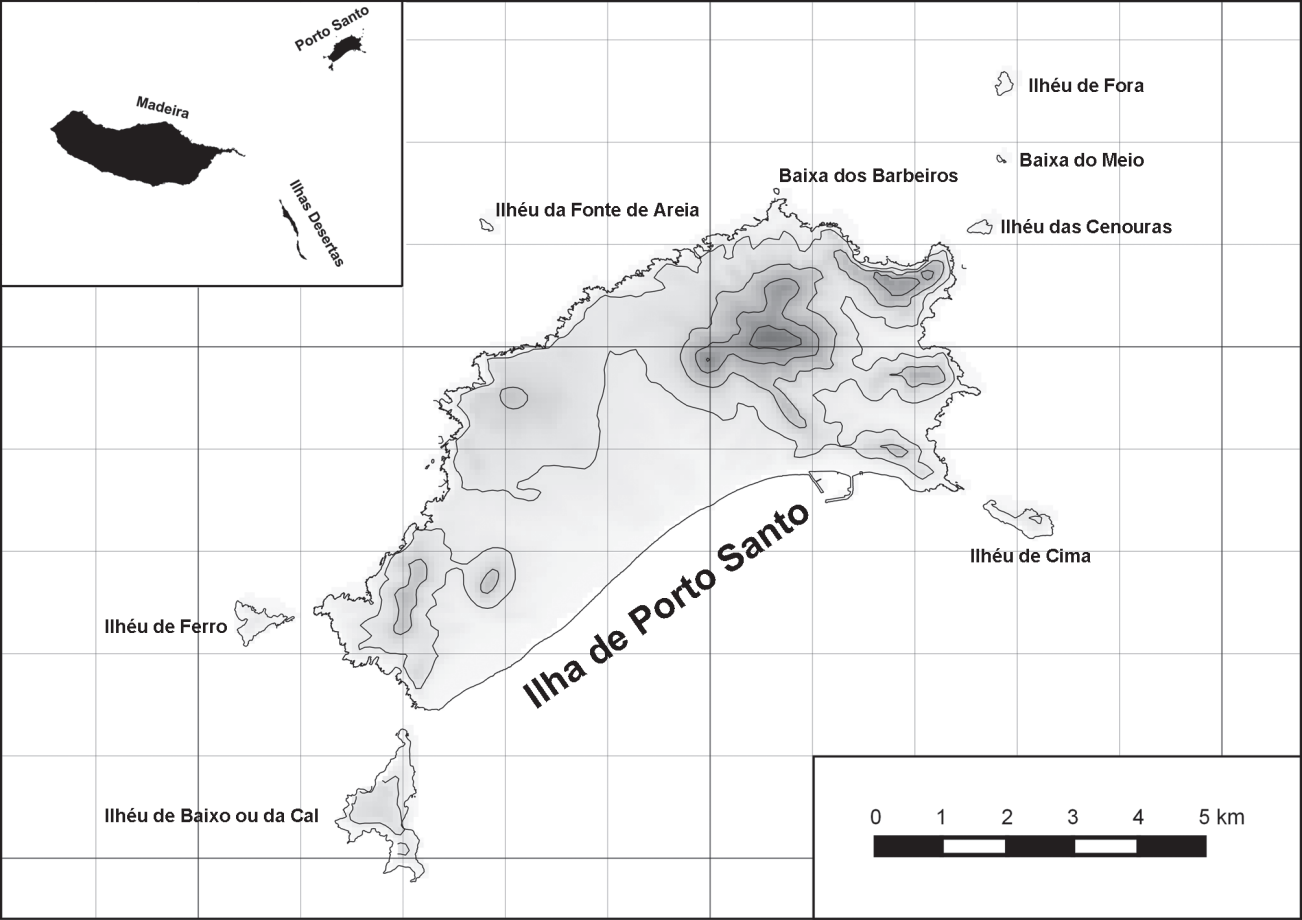
Map of Porto Santo with offshore islets and map of the Madeiran Archipelago in the Atlantic Ocean.

The island of Porto Santo (42.17 km²) is the northernmost island of the Madeiran Archipelago (Fig. 1), situated in the eastern Atlantic Ocean off the northwest African coast. As with the other islands of the archipelago, i.e., the island of Madeira and the Ilhas Desertas, Porto Santo is of volcanic origin, with its oldest subaerial deposits dated at 14.2 to 13.1 Ma before present to the Middle Miocene (Geldmacher et al. 2000; Ribeiro and Ramalho 2010). Thus, there was nearly three times as much time on Porto Santo for the evolution of an endemic land snail fauna compared to the island of Madeira with an estimated age of 5.2 to 4.6 Ma before present (Geldmacher et al. 2000; Ribeiro and Ramalho 2010) or to the Ilhas Desertas which are with approximately 3.6 Ma (Geldmacher et al. 2000), even younger than Madeira. The finding of a land snail from 20 to 13 Ma old Neogene deposits of Ilhéu de Cima, a satellite islet off southwestern Porto Santo, which Groh (1984) referred to Caseolus (Leptostictea) sp., suggests a relatively early colonization of Porto Santo by terrestrial gastropods belonging to lineages which are still present on the island today. Eleven genera and subgenera are currently considered to be endemic to Porto Santo incl. offshore islets: Leiostyla (Craticula) R. T. Lowe, 1852 (Lauriidae), Amphorella (Fusillus) R. T. Lowe, 1852 (Ferussaciidae), *Serratorotula* Groh & Hemmen, 1986, *Lemniscia* R. T. Lowe, 1855, *Pseudocampylaea* L. Pfeiffer, 1877, *Callina* R. T. Lowe, 1855, Discula (Mandahlia) Forcart, 1965 and *Hystricella* R. T. Lowe, 1855 (Geomitridae), Leptaxis (Katostoma) R. T. Lowe, 1855 (Hygromiidae), *Lampadia* Albers, 1854 and *Idiomela* T. D. A. Cockerell, 1921 (Helicidae). In total, 91 of the hitherto 102 known species and subspecies (c. 89%) recorded from Porto Santo are endemic (67 recent and 24 fossil taxa) resulting in a density of endemism of 2.13 taxa per square kilometre. Taking into consideration that most (sub-) species today only exist in the higher eastern part of the island, this value increases to 4.7 taxa per square kilometre, a value that is unique when measured on a global scale. This is especially noteworthy, since on Porto Santo, contrary to Madeira, the vertical differentiation of habitats was greatly reduced due to massive erosion in the past million years as evidenced by deeply cut ravines, relatively thick Aeolian deposits in the island’s central part (Ribeiro and Ramalho 2010) and a large marine abrasion platform surrounding the island, with abrasion values of 12 cm per year (Lietz and Schwarzbach 1971).

The documentation of the land snail diversity of Porto Santo started in 1824 when George Brettingham Sowerby I described the first four land snails from that island, among them *Helix bicarinata*, the type species of the genus *Hystricella*. The first more in-depth investigations of the terrestrial mollusc fauna of the Madeiran Archipelago were carried out by the clergyman Richard Thomas Lowe who visited the archipelago in 1828 for the first time. The results of his colleting efforts in that year were published in 1831 in his important synopsis of the fauna “Primitiæ Faunæ et Floræ Maderæ et Portus Sancti”, in which many of the land snails from Porto Santo considered as valid taxa today were named and described. From 1832 until 1854 R. T. Lowe served as Anglican chaplain in the archipelago and the material that he collected during these years formed the basis for twelve additional publications on the malacofauna of the archipelago (see Bank et al. 2002).

During this period only few additional taxa had been described, e.g., by Férussac (1832) and Deshayes (1835, 1850) in Férussac and Deshayes (1819–1851), Férussac (1835), Pfeiffer (1846), and Albers (1854). António da Costa de Paiva (Barão de Castelo de Paiva) described a number of species in 1866 and published a comprehensive thesis on the Madeiran land snail fauna in 1867, presenting an overview of the knowledge of the time.

Another important contribution towards an inventory of the malacofauna of the Madeira Archipelago was published by Thomas Vernon Wollaston under the title “Testacea Atlantica” in 1878, in which he summarised all the knowledge of the time on the non-marine molluscs of the Mid-Atlantic islands including the results of his five extended expeditions to the Madeiran Archipelago between 1847 and 1855. In this book, T. V. Wollaston listed ten taxa under the section Hystricella of the genus *Helix* Linnaeus, 1758, namely H. bicarinata var. aucta Wollaston, 1878, *H. bicarinata*, *H. echinoderma* Wollaston, 1878, *H. echinulata* R. T. Lowe, 1831, *H. leacockiana* Wollaston, 1878, *H. turricula* R. T. Lowe, 1831, *H. oxytropis* R. T. Lowe, 1831, H. turricula var. pererosa Wollaston, 1878, H. oxytropis var. subcarinulata Wollaston, 1878, and *H. vermetiformis* R. T. Lowe, 1855.

Subsequent researchers have not added any new taxa to the *Hystricella*-group, but evaluated its constituent taxa supra-specifically differently, without any substantial taxonomic basis, mostly placing them at a sub-generic level in the genus *Discula* R. T. Lowe, 1852. Furthermore, the status of many nominal taxa was, and still remains, controversial, ranging from treatments as mere varieties to subspecies or species. The two most up-to-date publications listed nine (Bank et al. 2002), viz. seven (Seddon 2008) specific and subspecific taxa in the genus *Hystricella*, the latter ignoring (except two) described fossil taxa from Quaternary deposits. Groh et al. (2009) published additions and corrections to Seddon (2008), including also three fossil taxa belonging to the genus *Hystricella*.

Anatomical investigations are currently lacking for most species currently placed in the genus *Hystricella*. The only exception is *H. turricula*, the anatomy of which was described by Watson (1923) in detail. Mandahl-Barth (1950) provided additional anatomical data for many geometrid taxa, but only repeated the results of Watson (1923) with regard to *Hystricella* because of the lack of preserved soft parts. Although Seddon (2008) provided distribution maps and added comments on the known distribution in the IUCN Red List (Seddon 2011a–e) for five recent taxa currently classified in *Hystricella*, some of the records presented in those publications seem to be erroneous or highly unlikely and ought to be critically revised.

In the present contribution, we present a comprehensive revision of the species currently assigned to the genus *Hystricella* including, aside from conchological comparisons, anatomical descriptions of all recent taxa (except for *W. vermetiformis*) as well as molecular phylogenetic analyses on the basis of mitochondrial as well as nuclear DNA sequences. Distribution data for all taxa are critically revised and stratigraphic as well as biogeographic relations are discussed.

## Materials and methods

### Collecting and preservation of samples

Recent, living snails were collected by hand. Although specimens were quite abundant in many populations (often reaching densities of more than 50 specimens per square meter), we limited sampling to a maximum of 15–20 specimens per site for ethical and conservation reasons. The developmental stage of each specimen was carefully checked during the collecting process and all juvenile and subadult specimens were left in the field. Half of the selected specimens were immediately fixed in absolute ethanol after collection in order to preserve the DNA for molecular investigations. The remaining specimens were drowned in water for 6–10 hours and subsequently fixed in 80% buffered ethanol. This was done to ensure that most of the distal parts of the snail’s body (foot, head, and distal genitalia) were stretched out from the shell, which facilitates dissection for morphological investigations. Two weeks after the first fixation, the ethanol solution was changed to remove the precipitate that mostly consists of mucus and denatured proteins. In addition to living specimens, empty shells from recent populations were collected to obtain a better impression of the shell morphology and its intra-specific as well as intra-population variability.

Most of the subfossil to fossil material was collected by hand. Fossiliferous deposits were identified directly in the field or with the help of a geological map. At some sites with aeolinite deposits sediment was collected and immediately sieved in the field in order to separate the sandy part from the coarser portion of the sample that was expected to contain fossil shells. Larger samples of strongly cemented material were carefully crushed in the field. The smaller pieces were brought to the laboratory where they were soaked in a 2% solution of hydrogen peroxide (H_2_O_2_) and then rinsed with boiling water to separate the shells and to remove any agglutinations and encrustations adhering to them. The shells cleaned in this way were again washed with water and finally dried.

### Morphological investigations

**Figure 2.**
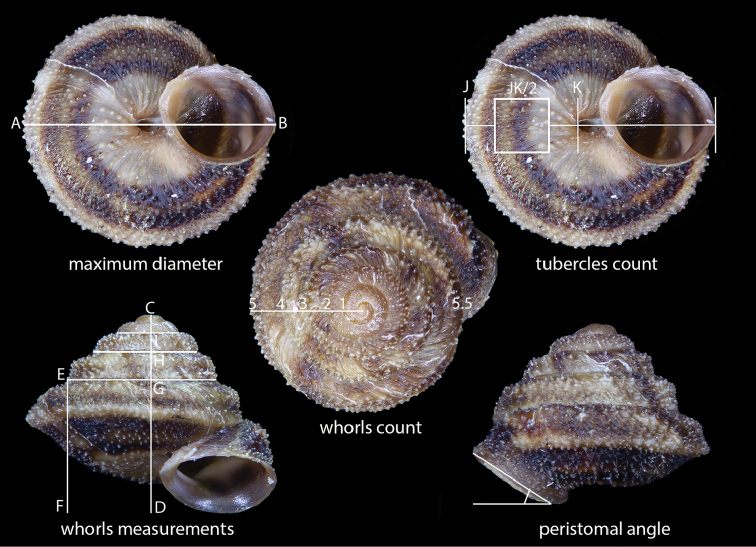
Standardisation method for counting the tubercles on the shell surface. **AB** Maximum diameter **CD** Maximum height **EF** height of last whorl **HG** height of second whorl **IC** height of third whorl; peristoma angle, measured as the angle between the peristomal plane and the horizontal axis of the shell **JK** width of the last whorl measured along the maximum diameter **(JK/2)^2^** square on the lower surface of the last whorl delimiting the area for the count of tubercles. The centre of the square corresponds to the mid point between J and K and the length of its sides is **JK/2**. This method allows to compare different specimens and taxa as the area of the square depends on the shell’s overall dimensions and is always proportional (the tubercles that partially traverse the contour of the square were included in the count of tubercles).

Shells were photographed with a Canon EOS camera equipped with 60 mm macro lenses mounted on a Kaiser microslide frame for multi-image stacking. The shells of all the dissected specimens and additional empty shells were photographed in front, bottom, side, and top view. Maximum shell diameter (D) and maximum shell height (H) were measured using a calliper. Additional measurement, i.e., height of the first (body) whorl (FW), peristomal angle (PA), diameter of umbilicus (DU), standardised number of tubercles on the shell surface (see Fig. 2), and the number of whorls of the protoconch, the teleoconch and their sum were taken using an image editing software. Mean values, standard deviations as well as D/H and FW/H ratios were calculated using MS Excel.

Dissections and examination of the anatomy were performed under a Zeiss stereoscope with a ring LED illumination apparatus, connected to a digital, high-resolution camera and a camera lucida. The genitalia were isolated from the rest of the body, usually after crushing the shell, using very fine and pointed micro-tweezers (Dumostar Biology 55) in a Petri dish with the bottom filled with black paraffin. The genitalia were fixed with very fine steel micro pins (commonly used for the preparation of microlepidopteran specimens in entomology). The internal features of genitalia (including cross and longitudinal sections) were examined after dissection with micro tweezers or by a pair of biological micro scissors (Aesculap OC series). Measurements were taken using a millimetric measurement scale. All examined genitalia were photographed in different positions (40 to 50 high-resolution images depicting all relevant anatomical features) in order to create an image database. Drawings were prepared by accurately tracing the most representative digital images after contrast enhancement with a picture editing software. The drawings, instead of digital photos, were used here because photos of the genitalia do not usually allow the reader to easily observe finer anatomical/morphological details of the soft tissues (such as small, fine pleats, striae, folds, papillae, ornamentations and diverticula). The drawings were performed when the samples were still displayed under the stereoscope in order to constantly check their morphology in case of doubtful situations.

For the preparation of jaws and radulae individual buccal masses were removed and immersed in a 2 M potassium hydroxide (KOH) solution for 5 h before extracting the jaw and the radula, which were then preserved in 70% ethanol for further investigations. Jaws and radulae were mounted and observed directly, i.e., without sputter coating, under a low vacuum SEM (Miniscope TM-1000, Hitachi High-Technologies, Tokyo).

### Molecular data preparation and phylogenetic analyses

For molecular genetic analyses samples of foot muscle tissue stored in 70–96% ethanol from representatives of all recent (sub-) species (except *H. vermetiformis*) and of species potentially belonging to the *Hystricella* group existing on the island of Porto Santo and of additional specimens of geomitrid species belonging to the nominal genera *Discula*, *Caseolus* R. T. Lowe, *Callina* and *Serratorotula* Groh & Hemmen, 1986 were used (see Table 1 for details). To root phylogenetic trees *Cochlicella conoidea* (Draparnaud, 1801) (Geomitridae, Geomitrinae, Cochlicellini) was included as outgroup (see Table 1). Total genomic DNA was extracted following a slightly modified version of the protocol of Sokolov (2000) as detailed in Scheel and Hausdorf (2012). Partial sequences of the mitochondrial cytochrome c oxidase subunit 1 (*cox1*) and (for a subset of samples representing all genus-group taxa, see Table 1) of the nuclear rRNA gene cluster (including partial sequences of the 18S rDNA, the complete internal transcribed spacer 1 (ITS1), 5.8S rDNA and internal transcribed spacer 2 (ITS2) as well as partial sequences of the 28S rDNA) were amplified by polymerase chain reaction (PCR) using the primer pairs LCO1490 plus HCO2198 (Folmer et al. 1994) and Hyg18S_Fw (5’-GCT ACT ATC GAT TGA GCG GTT CAG-3’, J. Harl, pers. comm.) and Hyg28S_Rv: 5’-CGT CCC ACA CAC CAC AGT T-3’, J. Harl, pers. comm.), respectively, following the protocols described in Neiber and Hausdorf (2015) and Harl et al. (2017), respectively. Both strands of the amplified products were sequenced at Macrogen Europe Laboratory (Amsterdam, The Netherlands). Information on vouchers and GenBank accession numbers are compiled in Table 1.

**Table 1.** Vouchers, locality data and GenBank accession numbers of Geomitrinae samples used for molecular analyses.

Taxon	Voucher number	Latitude / Longitude	GenBank accession number	
			*cox1*	18S rDNA + ITS1 + 5.8S rDNA + ITS2 + 28S rDNA
*Hystricella bicarinata*	HY03_2	33°04'16"N/16°18'53"W	MG575049	MG575189
*Hystricella bicarinata*	HY03_3	33°04'16"N/16°18'53"W	MG575050	–
*Hystricella bicarinata*	HY04_1	33°04'42"N/16°20'00"W	MG575051	MG575190
*Hystricella bicarinata*	HY10_1	33°04'51"N/16°18'41"W	MG575052	–
*Hystricella bicarinata*	HY10_2	33°04'51"N/16°18'41"W	MG575053	–
*Hystricella bicarinata*	HY10_3	33°04'51"N/16°18'41"W	MG575054	–
*Hystricella bicarinata*	HY32_1	33°04'38"N/16°19'48"W	MG575055	–
*Hystricella bicarinata*	HY32_2	33°04'38"N/16°19'48"W	MG575056	–
*Hystricella bicarinata*	HY33_1	33°04'52"N/16°19'26"W	MG575057	–
*Hystricella bicarinata*	HY33_2	33°04'52"N/16°19'26"W	MG575058	–
*Hystricella bicarinata*	HY33_3	33°04'52"N/16°19'26"W	MG575059	MG575191
*Hystricella bicarinata*	HY41_1	33°05'04"N/16°19'21"W	MG575060	–
*Hystricella bicarinata*	HY42_1	33°04'48"N/16°18'31"W	MG575061	–
*Hystricella bicarinata*	HY42_2	33°04'48"N/16°18'31"W	MG575062	–
*Hystricella bicarinata*	HY42_3	33°04'48"N/16°18'31"W	MG575063	–
*Hystricella bicarinata*	HY43_1	33°04'55"N/16°20'07"W	MG575064	–
*Hystricella bicarinata*	HY43_2	33°04'55"N/16°20'07"W	MG575065	–
*Hystricella bicarinata*	HY45_2	33°04'42"N/16°18'01"W	MG575066	–
*Hystricella bicarinata*	HY45_3	33°04'42"N/16°18'01"W	MG575067	MG575192
*Hystricella bicarinata*	HY47_1	33°05'01"N/16°19'14"W	MG575068	MG575193
*Hystricella bicarinata*	HY47_2	33°05'01"N/16°19'14"W	MG575069	–
*Hystricella bicarinata*	HY47_3	33°05'01"N/16°19'14"W	MG575070	–
*Hystricella bicarinata*	MN3003	33°05'02"N/16°19'14"W	MG575071	–
*Hystricella bicarinata*	MN3005	33°04'44"N/16°17'59"W	MG575072	–
*Hystricella bicarinata*	MN3008	33°04'15"N/16°18'55"W	MG575073	–
*Hystricella bicarinata*	MN3009	33°05'04"N/16°19'21"W	MG575074	–
*Hystricella bicarinata*	MN3014	33°04'52"N/16°19'26"W	MG575075	–
*Hystricella bicarinata*	MN3016	33°04'42"N/16°20'00"W	MG575076	–
*Hystricella bicarinata*	MN3018	33°04'32"N/16°19'38"W	MG575077	–
*Hystricella bicarinata*	MN3019	33°04'42"N/16°18'01"W	MG575078	–
*Hystricella bicarinata*	MN3020	33°04'55"N/16°20'07"W	MG575079	–
*Hystricella bicarinata*	MN3022	33°05'01"N/16°19'14"W	MG575080	–
*Hystricella bicarinata*	MN3023	33°04'51"N/16°18'41"W	MG575081	–
*Hystricella bicarinata*	MN3029	33°04'48"N/16°18'31"W	MG575082	–
*Hystricella bicarinata*	MN3047	33°04'44"N/16°17'59"W	MG575083	–
*Hystricella bicarinata*	MN3049	33°05'34"N/16°19'11"W	MG575084	–
*Hystricella echinulata*	HY39_1	33°05'29"N/16°18'14"W	MG575085	–
*Hystricella echinulata*	HY39_2	33°05'29"N/16°18'14"W	MG575086	–
*Hystricella echinulata*	HY39_3	33°05'29"N/16°18'14"W	MG575087	–
*Hystricella echinulata*	HY40_1	33°05'38"N/16°18'01"W	MG575088	–
*Hystricella echinulata*	HY40_2	33°05'38"N/16°18'01"W	MG575089	–
*Hystricella echinulata*	HY40_3	33°05'38"N/16°18'01"W	MG575090	–
*Hystricella echinulata*	MN3004	33°05'36"N/16°18'14"W	MG575091	–
*Hystricella echinulata*	MN3006	33°05'29"N/16°18'14"W	MG575092	–
*Hystricella echinulata*	MN3010	33°05'38"N/16°18'01"W	MG575093	–
*Hystricella bicarinataxechinulata*	HY06_1	33°05'42"N/16°19'23"W	MG575094	–
*Hystricella bicarinata x echinulata*	HY35_1	33°05'35"N/16°18'25"W	MG575095	–
*Hystricella bicarinata x echinulata*	HY35_2	33°05'35"N/16°18'25"W	MG575096	–
*Hystricella bicarinata x echinulata*	HY35_3	33°05'35"N/16°18'25"W	MG575097	–
*Hystricella bicarinata x echinulata*	HY36_1	33°05'57"N/16°19'32"W	MG575098	–
*Hystricella bicarinata x echinulata*	HY36_2	33°05'57"N/16°19'32"W	MG575099	–
*Hystricella bicarinata x echinulata*	HY36_3	33°05'57"N/16°19'32"W	MG575100	–
*Hystricella bicarinata x echinulata*	HY37_1	33°06'09"N/16°19'25"W	MG575101	–
*Hystricella bicarinata x echinulata*	HY37_2	33°06'09"N/16°19'25"W	MG575102	MG575194
*Hystricella bicarinata x echinulata*	MN3007	33°05'35"N/16°18'25"W	MG575103	–
*Hystricella bicarinata x echinulata*	MN3011	33°06'09"N/16°19'25"W	MG575104	–
*Hystricella bicarinata x echinulata*	MN3015	33°05'57"N/16°19'32"W	MG575105	–
*Hystricella bicarinata x echinulata*	MN3026	33°05'42"N/16°19'23"W	MG575106	–
*Hystricella bicarinata x echinulata*	MN3027	33°04'16"N/16°18'53"W	MG575107	–
*Wollastonia jessicae jessicae*	HY05_1	33°03'44"N/16°19'35"W	MG575108	MG575195
*Wollastonia jessicae jessicae*	HY05_2	33°03'44"N/16°19'35"W	MG575109	–
*Wollastonia jessicae jessicae*	HY05_3	33°03'44"N/16°19'35"W	MG575110	–
*Wollastonia jessicae jessicae*	HY34_1	33°03'47"N/16°19'41"W	MG575111	–
*Wollastonia jessicae jessicae*	HY34_2	33°03'47"N/16°19'41"W	MG575112	–
*Wollastonia jessicae jessicae*	HY34_3	33°03'47"N/16°19'41"W	MG575113	–
*Wollastonia jessicae jessicae*	MN3017	33°03'47"N/16°19'41"W	MG575114	–
*Wollastonia jessicae jessicae*	MN3021	33°03'44"N/16°19'35"W	MG575115	–
*Wollastonia jessicae monticola*	HY03_1	33°04'16"N/16°18'53"W	MG575116	–
*Wollastonia klausgrohi*	HY31_1	33°04'06"N/16°18'52"W	MG575117	MG575196
*Wollastonia klausgrohi*	HY31_2	33°04'06"N/16°18'52"W	MG575118	–
*Wollastonia klausgrohi*	HY31_3	33°04'06"N/16°18'52"W	MG575119	–
*Wollastonia klausgrohi*	MN3028	33°04'06"N/16°18'51"W	MG575120	–
*Wollastonia leacockiana*	HY01_1	33°02'57"N/16°22'04"W	MG575121	–
*Wollastonia leacockiana*	HY01_2	33°02'57"N/16°22'04"W	MG575122	MG575197
*Wollastonia leacockiana*	MN3002	33°02'39"N/16°22'11"W	MG575123	–
*Wollastonia leacockiana*	MN3024	33°02'57"N/16°22'04"W	MG575124	–
*Wollastonia oxytropis*	HY38_1	33°04'42"N/16°17'56"W	MG575125	MG575198
*Wollastonia oxytropis*	MN3001	33°04'43"N/16°18'19"W	MG575126	–
*Wollastonia oxytropis*	MN3013	33°04'42"N/16°17'56"W	MG575127	–
*Wollastonia oxytropis*	MN3048	33°04'41"N/16°17'57"W	MG575128	–
*Wollastonia turricula*	MN3000	33°03'13"N/16°16'48"W	MG575129	–
*Callina bulverii*	DI04_1	33°04'51"N/16°18'41"W	MG575130	–
*Callina bulverii*	DI15_2	33°04'16"N/16°18'53"W	MG575131	MG575199
*Callina bulverii*	DI15_3	33°04'16"N/16°18'53"W	MG575132	–
*Callina bulverii*	DI27_1	33°04'06"N/16°18'52"W	MG575133	–
*Callina bulverii*	DI28_1	33°04'38"N/16°19'48"W	MG575134	–
*Callina rotula*	DI23_1	33°05'35"N/16°18'25"W	MG575135	–
*Callina rotula*	DI25_1	33°04'42"N/16°17'51"W	MG575136	–
*Callina rotula*	DI26_1	33°05'57"N/16°19'32"W	MG575137	–
*Callina rotula*	DI31_1	33°06'09"N/16°19'25"W	MG575138	–
*Callina rotula*	DI31_2	33°06'09"N/16°19'25"W	MG575139	–
*Callina rotula*	DI35_1	33°04'48"N/16°18'31"W	MG575140	–
*Callina rotula*	DI35_2	33°04'48"N/16°18'31"W	MG575141	–
*Callina rotula*	DI37_1	33°05'29"N/16°18'14"W	MG575142	–
*Callina rotula*	DI37_2	33°05'29"N/16°18'14"W	MG575143	–
*Callina rotula*	DI44_1	33°05'38"N/16°18'01"W	MG575144	–
*Callina rotula*	DI44_2	33°05'38"N/16°18'01"W	MG575145	MG575200
*Callina rotula*	MN3051	33°04'48"N/16°18'31"W	MG575146	–
Discula (Discula) attrita	DI05_1	33°04'44"N/16°18'37"W	MG575147	MG575201
Discula (Discula) attrita	DI05_2	33°04'44"N/16°18'37"W	MG575148	–
Discula (Discula) attrita	DI05_3	33°04'44"N/16°18'37"W	MG575149	–
Discula (Discula) attrita	DI12_1	33°02'57"N/16°22'04"W	MG575150	–
Discula (Discula) attrita	DI12_2	33°02'57"N/16°22'04"W	MG575151	–
Discula (Discula) attrita	DI21_2	33°03'37"N/16°17'55"W	MG575152	–
Discula (Discula) attrita	DI29_1	33°03'45"N/16°17'57"W	MG575153	–
Discula (Discula) attrita	DI29_2	33°03'45"N/16°17'57"W	MG575154	–
Discula (Discula) attrita	DI29_3	33°03'45"N/16°17'57"W	MG575155	–
Discula (Discula) attrita	DI29_4	33°03'45"N/16°17'57"W	MG575156	–
Discula (Discula) attrita	DI29_5	33°03'45"N/16°17'57"W	MG575157	–
Discula (Discula) attrita	DI34_1	33°04'48"N/16°18'31"W	MG575158	–
Discula (Discula) attrita	DI38_1	33°02'38"N/16°22'56"W	MG575159	–
Discula (Discula) attrita	DI38_2	33°02'38"N/16°22'56"W	MG575160	–
Discula (Discula) attrita	DI43_2	33°04'01"N/16°18'26"W	MG575161	–
Discula (Discula) attrita	MN3053	-	-	MG575162	–
Discula (Discula) calcigena	DI42_1	33°04'16"N/16°17'58"W	MG575163	MG575202
Discula (Discula) discina	DI08_1	33°05'48"N/16°18'54"W	MG575164	–
Discula (Discula) discina	DI08_2	33°05'48"N/16°18'54"W	MG575165	–
Discula (Discula) discina	DI08_3	33°05'48"N/16°18'54"W	MG575166	–
Discula (Discula) discina	DI08_4	33°05'48"N/16°18'54"W	MG575167	–
Discula (Discula) discina	DI16_1	33°05'25"N/16°20'58"W	MG575168	–
Discula (Discula) discina	DI16_2	33°05'25"N/16°20'58"W	MG575169	–
Discula (Discula) discina	DI16_3	33°05'25"N/16°20'58"W	MG575170	–
Discula (Discula) discina	DI16_4	33°05'25"N/16°20'58"W	MG575171	–
Discula (Discula) discina	DI22_1	33°04'44"N/16°18'37"W	MG575172	–
Discula (Discula) discina	DI22_2	33°04'44"N/16°18'37"W	MG575173	–
Discula (Discula) discina	DI22_3	33°04'44"N/16°18'37"W	MG575174	–
Discula (Discula) discina	DI22_4	33°04'44"N/16°18'37"W	MG575175	–
Discula (Discula) discina	DI24_1	33°05'48"N/16°18'54"W	MG575176	–
Discula (Discula) discina	DI24_2	33°05'48"N/16°18'54"W	MG575177	MG575203
Discula (Discula) polymorpha alleniana	MN3052	32°52'03"N/17°09'56"W	MG575178	–
Discula (Discula) polymorpha arenicola	DI06_1	32°44'41"N/16°41'58"W	MG575179	MG575204
Discula (Discula) polymorpha arenicola	DI06_2	32°44'41"N/16°41'58"W	MG575180	MG575205
Discula (Discula) pulvinata	DI30_1	33°05'07"N/16°21'21"W	MG575181	MG575206
Discula (Discula) pulvinata	DI30_2	33°05'07"N/16°21'21"W	MG575182	–
Discula (Discula) pulvinata	DI30_3	33°05'07"N/16°21'21"W	MG575183	–
Discula (Discula) tabellata	DI03_1	32°38'21"N/16°51'03"W	MG575184	–
Discula (Discula) tabellata	DI03_2	32°38'21"N/16°51'03"W	MG575185	–
‘Discula’ testudinalis	MN3077	33°06'14"N/16°19'18"W	MG575186	MG575207
Caseolus (Caseolus) innominatus innominatus	CA08_1	32°52'03"N/17°09'56"W	MG575187	MG575208
Caseolus (Leptostictea) hartungi	CA10_1	33°05'15"N/16°21'16"W	MG575188	MG575209
*Serratorotula juliformis*	MN3036	33°03'45"N/16°17'57"W	MG575210	–
Cochlicella (Cochlicella) conoidea	SP165	41°08'15"N/08°40'00"W	KY818425 ^1^	KY818623 ^1^

^1^: Neiber et al. (2017)

ChromasPro 1.7.1 (Technelysium) software was used to assemble forward and reverse sequence reads. Nuclear rDNA sequences were aligned with MAFFT 7 (Katoh and Standley 2013) using the Q-INS-i iterative refinement algorithm, whereas *cox1* sequences were aligned with MUSCLE (Edgar 2004) as implemented in MEGA 6.0 (Tamura et al. 2013). We used maximum likelihood (ML), Bayesian Inference (BI) and maximum parsimony (MP) approaches to reconstruct the phylogenetic relationships. Appropriate evolutionary models for the mitochondrial and nuclear sequence data sets were selected with PartitionFinder 1.1.1 (Lanfear et al. 2012) conducting an exhaustive search with separate estimation of branch lengths for each partition and with the Bayesian information criterion (as recommended by Luo et al. 2010) as criterion to select among models. As the nuclear ribosomal DNA sequence were hardly variable among ingroup and outgroup taxa, nuclear sequence data were not divided further into partitions. The models to choose from were restricted to those available in MrBayes 3.2.6 (Ronquist et al. 2012) as well as in Garli 2.1 (Zwickl, 2006). As best-fit partitioning scheme, the PartitionFinder analysis suggested to use the HKY + I + G model for the mitochondrial sequences and the GTR + I + G model for the nuclear sequences.

BI analysis was performed using MrBayes. Metropolis-coupled Monte Carlo Markov chain (MC^3^) searches in MrBayes were run with four chains in two separate runs for 50,000,000 generations with default priors, trees sampled every 1,000 generations under default heating using the best-fit model as suggested by PartitionFinder. Diagnostic tools in MrBayes, including Effective Sample Size (ESS) values > 200, Potential Scale Reduction Factors ~ 1.000 and an average standard deviation of split frequencies < 0.01, were used to ensure that the MC^3^ searches had reached stationarity and convergence. The first 5,000,000 generations of each run were discarded as burn-in.

ML analyses were performed with Garli and iqtree 1.5.3 (Nguyen et al. 2015) using the best-fit models as suggested by PartitionFinder and otherwise default settings. Support values were computed by bootstrapping (BS) with 1,000 replications.

MP searches were carried out with PAUP* 4.0b10 (Swofford 2002) using 1,000 random-addition-sequence replicates and TBR branch swapping. Support values were computed by bootstrapping with 1,000 replications.

BS values above 70% are interpreted as meaningful node support and PP values above 0.95 as statistically significant support.

### Collection acronyms

**ANSP** Academy of Natural Sciences of Drexel University, Philadelphia, PA, USA

**CFW** private collection of Frank Walther, Essen, Germany

**CJG** private collection of Jochen Gerber, Chicago, USA

**CKG** private collection of Klaus Groh, Bad Dürkheim, Germany

**CMN** private collection of Marco T. Neiber, Sehnde, Germany

**CWDM** private collection of Willy De Mattia, Trieste, Italy

**DRAM** Direcção Regional do Ambiente da Madeira, Funchal, Madeira, Portugal

**GNM** Göteborgs Naturhistorika Museum, Göteborg, Sweden

**HNHM** Hungarian National History Museum, Budapest, Hungary

**NHC** Natural History Collections, University of Edinburgh, Edinburgh, Great Britain

**NHM** Natural History Museum, London, Great Britain

**NHMW** Natural History Museum Wien, Austria, Malacological collection

**NMS** National Museum of Scotland, Edinburgh, Great Britain

**MMF** Museu de Historia Natural do Funchal (formerly Museo Municipal do Funchal), Funchal, Madeira, Portugal

**MMUE** Manchester Museum, University of Manchester, Great Britain

**NMW** National Museum of Wales, Cardiff, Great Britain

**OUMNH** Oxford University Museum of Natural History, Oxford, Great Britain

**RAM** Royal Albert Memorial Museum, Exeter, Great Britain

**SMF** Forschungsinstitut und Naturmuseum Senckenberg, Frankfurt/M., Germany

**ZMH** Zoological Museum Hamburg, Hamburg, Germany

### Morphological abbreviations

**Genital anatomy**

**A** atrium

**AF** atrial folds

**AG** albumen gland

**BC** bursa copulatrix

**DBC** duct of bursa copulatrix

**DP** distal penis

**F** flagellum

**FAP** accessory papilla of the flagellum

**FO** free oviduct

**FMP** main papilla of the flagellum

**LDL** left dorsal lobe

**LSPP** longitudinal section of penial papilla

**MR** retractor muscle

**OSD** ovispermiduct

**PC** penial channel

**PCE** penial channel exit

**PM** penial matrix

**PN** pneumostoma

**PP** penial papilla

**PW** penial wall

**SL** subpneumostomal lobe

**RLL** right lateral lobe

**SP** spongy pilaster of the distal penis

**SVA** single vaginal appendage (dartless stylphore)

**TSPP** transversal section of penial papilla

**V** vagina

**VD** vas deferens

**VG** vaginal glands

**VS** vaginal section

**Shell**

**D** maximum shell diameter

**H** maximum shell height

**FW** height of the first (body) whorl

**NT** number of tubercles

**NW** number of whorls

**PA** peristomal angle

**DU** diameter of umbilicus

Taxa exclusively known from fossil material are indicated by a dagger symbol (†).

## Results and discussion

### Phylogenetic analyses

**Figure 3.**
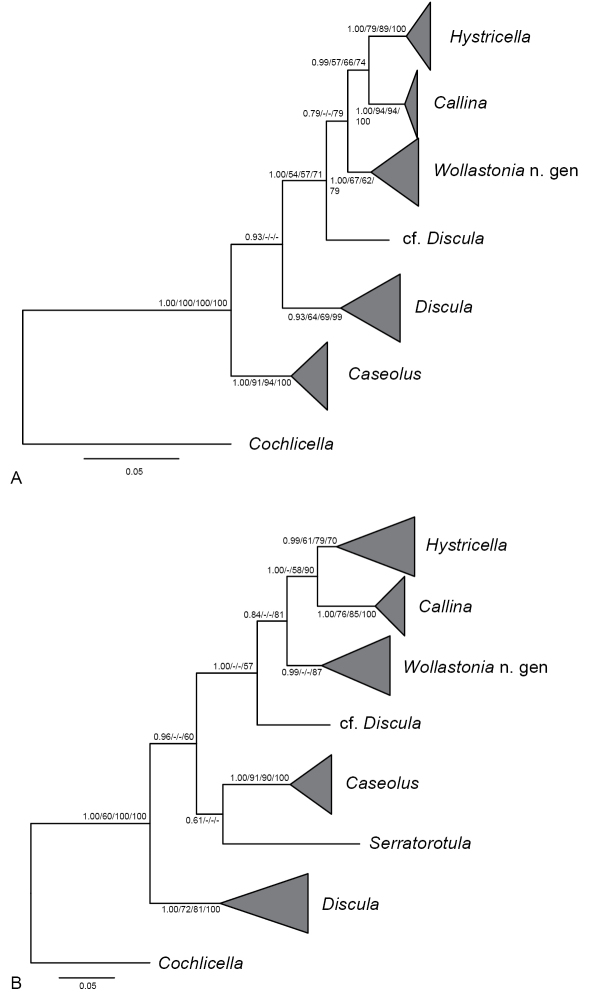
Bayesian 50% majority rule consensus tree based (**A**) on the concatenated sequences of *cox1* and 18S rDNA + ITS1 + 5.8S rDNA + ITS2 + 28S rDNA and (**B**) on the *cox1* sequences alone. Posterior probability values (first value), bootstrap support values from the maximum likelihood analysis with Garli (second value) and iqtree (third value) and maximum parsimony analysis with PAUP* (fourth value) are indicated at the nodes. Only posterior probability values ≥ 0.5 and bootstrap support values ≥ 50% are shown. For phylogenetic relationships of *Hystricella*, *Wollastonia* gen. n., *Callina*, and *Discula* species included in the analyses, see Figure 4–6.

The phylogenetic analyses, both based on the mitochondrial *cox1* data alone and the concatenated mitochondrial and nuclear data recovered the included Geomitrini as a mostly well-supported monophyletic group (Fig. 3). Within this clade, several groups, here interpreted as genus-group taxa, were mostly significantly/meaningfully supported (Fig. 3), albeit relationships among these groups did not receive significant/meaningful support in all analyses. Neither the genus *Hystricella* nor the genus *Discula* in the sense of Bank et al. (2002), Seddon (2008), Groh et al. (2009) or Bank (2009) were recovered as monophyletic groups. Species formerly included in *Hystricella* were assigned to two different clades (Fig. 3).

**Figure 4.**
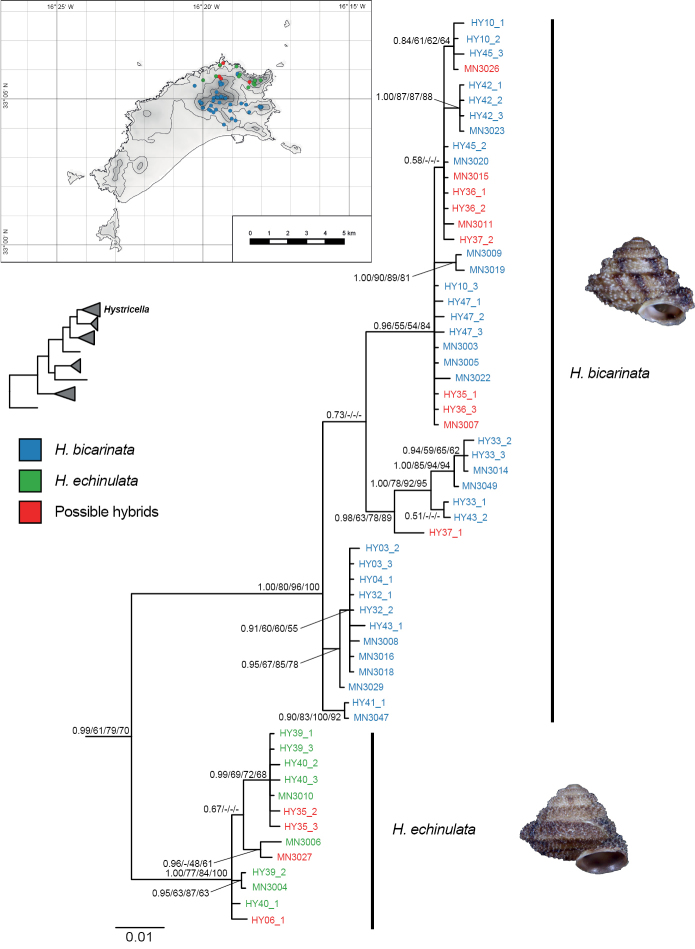
Bayesian 50% majority rule consensus tree based on *cox1* sequences of *Hystricella* species. Posterior probability values (first value), bootstrap support values from the maximum likelihood analysis with iqtree (second value) and Garli (third value) and maximum parsimony analysis with PAUP* (fourth value) are indicated at the nodes. Only posterior probability values ≥ 0.5 and bootstrap support values ≥ 50% are shown. The inset map shows the distribution of *H. bicarinata* (blue), *H. echinulata* (green) and putative hybrids of both species (red) on Porto Santo. The tip labels on the tree are coloured accordingly. For information on vouchers, see Table 1 and the corresponding material lists under the species’ sections. The inset tree shows the position of *Hystricella* in relation to the other investigated genus-group taxa based on the analysis of concatenated mitochondrial and nuclear sequences as shown in Figure 3.

The clade including the type species of *Hystricella*, *H. bicarinata*, also included *H. echinulata* and received significant/meaningful support in all phylogenetic analyses except in the ML analysis with Garli based on *cox1* data alone (Fig. 4). Within the *Hystricella* clade two well-separated clades which largely correspond to specimens morphologically assigned to either *H. bicarinata* or *H. echinulata* were recovered (Fig. 4). *Hystricella bicarinata* has its distribution centre in the eastern central to southeastern part of Porto Santo, whereas *H. echinulata* is restricted to the northeastern part of the island (Fig. 4). In a small area in the north of Porto Santo populations with intermediate or deviating morphologies (i.e., presence/absence of a double keel on the body whorl and presence/absence of papilla on the inner wall of the terminal part of the flagellum) were found. In the *cox1* phylogenies these specimens, either clustered with *H. bicarinata* or *H. echinulata* (Fig. 4) and are here interpreted as putative hybrids between the two species.

**Figure 5.**
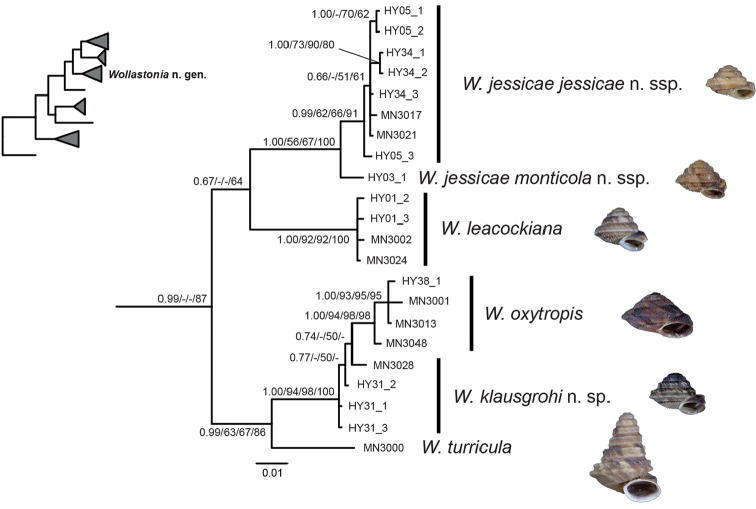
Bayesian 50% majority rule consensus tree based on *cox1* sequences of *Wollastonia* gen. n. species. Posterior probability values (first value), bootstrap support values from the maximum likelihood analysis with Garli (second value) and iqtree (third value) and maximum parsimony analysis with PAUP* (fourth value) are indicated at the nodes. Only posterior probability values ≥ 0.5 and bootstrap support values ≥ 50% are shown. For information on vouchers, see Table 1 and the corresponding material lists under the species’ sections. The inset tree shows the position of *Wollastonia* gen. n. in relation to the other investigated genus-group taxa based on the analysis of concatenated mitochondrial and nuclear sequences as shown in Figure 3.

The other clade including species hitherto assigned to *Hystricella*, although only supported in the BI and MP analyses (Figs 3, 5), will be formally described as *Wollastonia* gen. n. below. This clade includes *W. leacockiana* comb. n., *W. turricula* comb. n., and *W. oxytropis* comb. n., as well as three additional recent taxa that will be formally described below: *W. klausgrohi* sp. n., *W. jessicae* sp. n., and *W. jessicae monticola* ssp. n. (Fig. 5). The relationships of most of the taxa within *Wollastonia* gen. n. could not be well resolved (Fig. 5) on the basis of the phylogenetic analyses of the *cox1* data. Specifically, although the monophyly of *W. oxytropis* comb. n. was well-supported in all analyses, the phylogenetic analyses failed to recover the monophyly of *W. klausgrohi* sp. n. (Fig. 5). However, the very distinct shell morphology and genital anatomy of *W. oxytropis* comb. n. (see below) clearly supports the recognition of two distinct, though closely related species.

**Figure 6.**
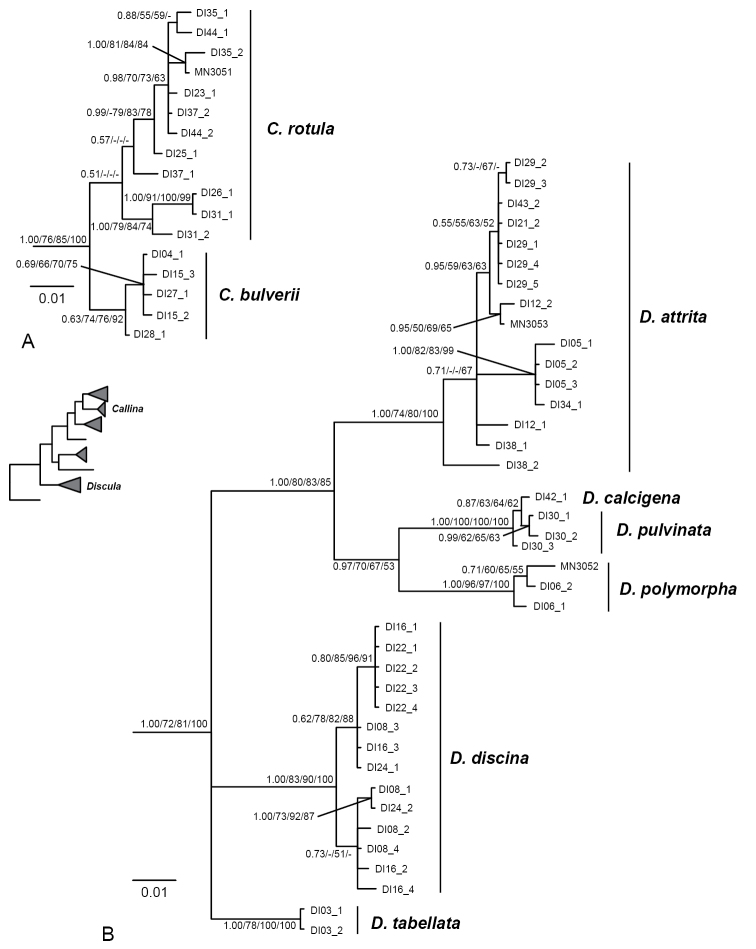
Bayesian 50% majority rule consensus tree based on *cox1* sequences of (**A**) *Callina* and (**B**) *Discula* species. Posterior probability values (first value), bootstrap support values from the maximum likelihood analysis with Garli (second value) and iqtree (third value) and maximum parsimony analysis with PAUP* (fourth value) are indicated at the nodes. Only posterior probability values ≥ 0.5 and bootstrap support values ≥ 50% are shown. For information on vouchers, see Table 1 and the corresponding material lists under the species’ sections. The inset tree shows the position of *Callina* and *Discula* in relation to the other investigated genus-group taxa based on the analysis of concatenated mitochondrial and nuclear sequences as shown in Figure 3.

Interspersed between the *Hystricella* and *Wollastonia* gen. n. clades, a clade containing *Callina rotula* (R. T. Lowe, 1831), the type species of *Callina* R. T. Lowe, 1855, was placed as the sister group of *Hystricella* with significant/meaningful support in the BI and MP analyses (Figs 3, 6A). *Callina* was up till now either regarded as a monospecific genus related to the genus *Discula* (Waldén 1983; Schileyko 2005), or included as a subgenus in *Discula*, but the phylogenetic analyses presented here do not support the monophyly of *Callina* plus *Discula* s. str. (i.e., the clade including the type species of the genus *D. discina* (R. T. Lowe, 1852), see Figs 3, 6A). Interestingly, the phylogenetic analyses recovered a close relationship of *C. rotula* and *C. bulverii* (Wood, 1828), comb. n., a taxon up till now included in *Discula* s. str. (Bank et al. 2002; Seddon 2008; Bank 2009).

In the phylogenetic analyses, both based on the concatenated and the *cox1* data set, a specimen of *Discula testudinalis* (R. T. Lowe, 1852) from Porto Santo was placed in a sister group relationship to the clade including *Hystricella*, *Wollastonia* gen. n. and *Callina*, albeit not supported in all analyses (Fig. 3). The remaining *Discula* species were grouped together with statistical support in the BI and MP analyses based on the concatenated mitochondrial and nuclear data and in all analyses based on the *cox1* data alone (Fig. 6B). Within the *Discula* clade three well-supported clades were placed in a polytomy (Fig. 6B): the first of these clades grouped two specimens of *D. tabellata* (R. T. Lowe, 1852) from Madeira together, the second group included specimens of *D. discina* (R. T. Lowe, 1852) from Porto Santo and the third clade included specimens of *D. attrita* (R. T. Lowe, 1831), *D. pulvinata* (R. T. Lowe, 1831) and *D. calcigena* (R. T. Lowe, 1831) from Porto Santo as well as *D. polymorpha* (R. T. Lowe, 1831) from Madeira. Within the latter clade, *D. attrita* was recovered as a mostly well-supported sister group of the remaining three taxa, and *D. polymorpha* as the sister group of a clade containing *D. calcigena* and *D. pulvinata* (Fig. 6B). *Discula pulvinata* was recovered as a paraphyletic group with respect to *D. pulvinata*, however, divergences within this clade were rather low overall and the branching patterns not well-resolved.

### Systematic account

The suprageneric classification follows Razkin et al. (2015) and Neiber et al. (2017), elevating the Geomitridae from subfamily to family rank with Geomitridae plus Canariellidae Schileyko, 1991 as the sister group of Hygromiidae Tryon, 1866.

**Helicoidea** Rafinesque, 1815

**Geomitridae** C. R. Boettger, 1909

**Geomitrinae** C. R. Boettger, 1909

Syn. Ochthephilinae Zilch, 1960 in Wenz and Zilch (1959–1960)

**Geomitrini** C. R. Boettger, 1909

#### Hystricella

Taxon classificationAnimaliaStylommatophoraGeomitridae

R. T. Lowe, 1855

Discula auct., partim [non R. T. Lowe, 1852].
Helix (Ochthephila) sensu Albers (1850) and Albers (1854), partim.
Helix (Octephila) , Paiva (1867) [incorrect subsequent spelling Ochthephila Albers, 1850], partim.
Geomitra (Actinella (Hystricella)), Pilsbry (1893–1895).
Geomitra sensu Bank et al. (2002).

##### Type species, by original designation in R. T. Lowe (1855).

*Helix bicarinata* G. B. Sowerby I, 1824 = *Hystricella bicarinata* (G. B. Sowerby I, 1824).

Lowe (1855: 186–187) describes the new genus *Hystricella* in the section § 24, basing his description upon shell features only, as follows:

(Typ. *H. bicarinata*, Sow.). T. perf. v. angustae umbil. conuloidea v. trochiformis, aliquando turrito-pyramidata acute v. distincte 1–2-carinata solidiuscula aspero-granulata v. echinulata subfasciata. Anfr. 6–9 lente crescentes planiusculi, ult. valde carinato subtus planato, antice deflexo. Umbil. parvus anguste cylindricus v. subspiralis constrictus. Apert. circularis circinata labris connexis; perist. continuum solutum expanso-reflexiusculum tenue acutum.

Lowe (1855: 186–187) cites for the genus (under section § 24) the following species:

*H. bicarinata* G. B. Sowerby I, 1824

*H. echinulata* R. T. Lowe, 1831

*H. oxytropis* R. T. Lowe, 1831

*H. turricula* R. T. Lowe, 1831

*H. vermetiformis* R. T. Lowe, 1855

*Hystricella mustelina* Reeve, 1854 [under authorship R. T. Lowe, 1855], currently considered as a subspecies of Discula (Discula) cheiranthicola Reeve, 1854 [even though the description was prepared by R. T. Lowe in 1854 the work was only published in 1855 and therefore the earlier publication of that name in Reeve in 1854 has priority].

*Hystricella cheiranticola* R. T. Lowe, 1831 and its morphae (later put in the mono-specific subgenus [as section] *Turritella* by Wollaston 1878 [non Lamarck 1799]).

Later, *H. mustelina* (currently Discula (Discula) cheiranthicola mustelina (Reeve, 1854)) and *H. cheiranticola* (currently Discula (Discula) cheiranthicola cheiranthicola (R. T. Lowe, 1831)) were assigned to the genus *Discula* R. T. Lowe, 1852 (Mandahl-Barth 1950).

##### Description of the genus.

**Shell.** The shell is dextral, hairless and it is usually conical and scalariform. The protoconch is whitish to dark brown, with 1.3 to 2.2 whorls. It is almost smooth along the first whorl and shows fine radial striae and extremely small, scattered tubercles along its remaining portion. The teleoconch has from 4.2 to 5.0 rapidly increasing whorls. It is usually dark brown with brick red and/or dark violet colour shades. The dark areas of the shell are mottled with more or less light brown to whitish areas, usually placed longitudinally and slightly slanting. No band pattern is visible along the upper whorls. On the lower part of the last whorl two well-defined, dark bands are visible that can differ in width. The area around the umbilicus is usually the lightest in colour. The spire is slightly variable in height, ranging in shape from compressed to somewhat more elevated. Along the last and the penultimate whorls one to two evident keels are present. The external upper surface has very fine but clearly visible, irregularly spaced growth lines. Along the last whorls the growth lines usually disappear along the lateral area and reappear on the lower part. Irregularly disposed tubercles are found all over the teleoconch. The dimensions of the tubercles tend to increase slightly from the first toward the last whorl, the density remaining, however, approximately the same. The tubercles are somewhat denser along the keels of the penultimate and last whorls, letting the keel/keels appear like a rough chord. On the lower part of the last whorl the tubercles are usually bigger and slightly less dense than in the remaining parts of the shell. The last whorl is large, with a contribution of 60% of the total shell height and descending towards the aperture. The umbilicus is open but very narrow, concentric and has a diameter of approximately 10% of the maximum shell diameter. The aperture is elliptical with a faint thickening along the inner side of the last whorl. The peristome is continuous, slightly reflected with the columellar margin somewhat thicker and more reflected.

**Body.** Head and neck are grey to pale grey, slightly transparent. The sides and posterior upper section of the foot are whitish. The sole of the foot is whitish and longitudinally divided into three areas. The central area is smooth whereas the two lateral areas are equipped with bands of muscles roughly arranged in a chevron pattern. The mantle border is grey to dark grey with five more or less developed lobes. The ratio among lateral and dorsal lobes varies from specimen to specimen also in the same population. In some specimens, particular lobes (regardless if lateral or dorsal) may be completely missing. The walls of the pallial cavity are colourless, without any stripes or spots. A strong pulmonary vein is visible.

**Genitalia.** The general arrangement of the genitalia is semi-diaulic monotrematic. A convoluted to almost straight first hermaphroditic duct arises from a multi-lobated gonad. The albumen gland is long and moderately thin and connected to an equally long sperm-oviduct consisting of a prostatic and a uterine portion. Distally, the prostatic part extends into a thin vas deferens, twice as long as the sperm-oviduct, terminating in the penial complex. The distal portion of the uterine part inserts into the free oviduct, then transforming into a vagina at the level of the insertion of the duct of the bursa copulatrix. The free oviduct is usually three to four times shorter than the vagina. The duct of the bursa copulatrix is usually wide, approximately as long as the penis and uniform in diameter. This duct terminates in a variable, oval to roundish, small bursa copulatrix. The spermatophore is unknown. One tuft of digitiform glands arises from the proximal part of the vagina. There are usually two or three, equally long and very rarely branched glands. A short and thin vaginal appendix arises from the vagina’s wall, immediately distal of the glandular tuft. Very smooth, little elevated and spaced pleats run longitudinally along the inner surface of the vagina, reaching into the genital atrium as far as the genital orifice. The atrium can be short or moderately long. The penial complex consists of a flagellum, an epiphallus (which extends from insertion of the vas deferens to the penial retractor muscle) and a penis that inserts into the genital atrium. The penial flagellum is short, remarkably cylindrical and with a blunt apex. It is usually twice as long as the epiphallus. Its internal walls can be either completely smooth or ornamented with very small papillae distributed mainly close to the blunt end. The epiphallus is usually extremely short and its internal walls are smooth. The retractor muscle is well developed, strong and is variable in length. The penis lacks a muscular or glandular sheath. It is thick-walled and approximately four times longer than the flagellum. It is usually cylindrical to sometimes slightly swollen in its distal part. The inner walls of the penis are usually smooth or with very smooth, little elevated and spaced pleats which run longitudinally and reach the genital atrium. The section where the penial papilla is located is usually detectable from the outside by virtue of a fine circular swelling corresponding to the origin of the papilla itself. The penial papilla is very variable in size, ranging from 1/6 to 1/2 of the total penial length and is conical to subcylindrical in shape. The inner lumen of the penial papilla is filled with a spongy and sturdy tissue which directly connects with the walls of the epiphallus. It has smooth external walls with the opening emerging apically. The channel of the penial papilla is thin and narrow. The longitudinal section of the penial papilla shows that its walls are the continuation of the penial walls that abruptly bend inward.

**Jaw and radula.** The jaw and radular apparatus of *Hystricella* is depicted in Fig. 8. No notable variability was found among the species of the genus. The jaw is odontognathous, almost straight to markedly arched, with 13 to 15 smooth ridges. The radula ribbon is typical helicoid, it is elongated but not very slender. Central tooth present, tricuspid, the main cusp (endocone) is rhomboid, pointed; the ectocones are much smaller than the endocone; they are triangular, pointed. There are 19–20 laterals and marginals which do not distinctly differ from each other, i.e., their shape changes gradually from the first laterals towards the marginals. Laterals are bicuspid, endocones are rhomboid or triangular and pointed. The ectocones are much smaller, pointed, and triangular. The endocones of the central tooth and the first laterals are approximately of the same size. Both, the endocone and the ectocone of the laterals gradually become bifurcated towards the marginal teeth, but the ectocones may occasionally have three cusps as well. The cusps of the marginals are gradually decreasing in size; therefore, the outermost marginals appear nearly serrated.

##### Distribution.

**Figure 7.**
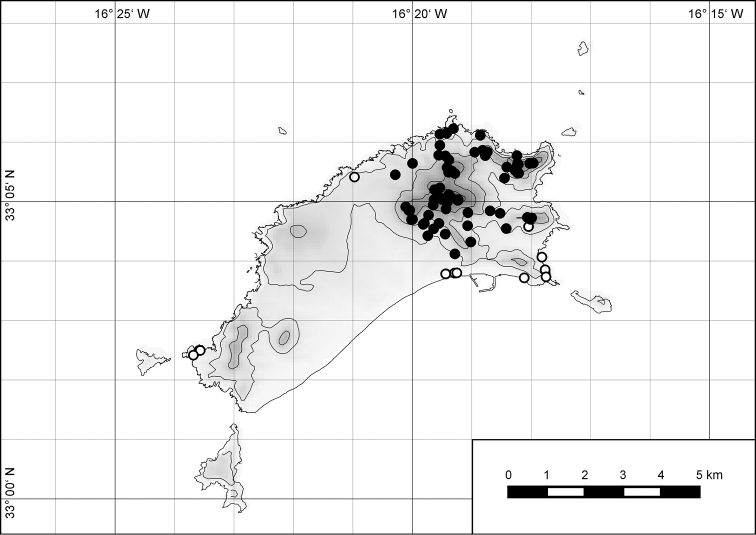
Distribution of the genus *Hystricella*. Filled circles refer to recent and open circles to fossil records.

The genus *Hystricella* is endemic to the island of Porto Santo (Madeiran Archipelago, Portugal). The genus is restricted to the eastern, mountainous part of the island and occurs there only in the central-northern section of this area (Fig. 7). Species assigned to the genus are commonly found on the slopes of Pico do Castelo, Pico do Facho, Pico de Juliana, Pico da Gandaia, Rocha de Nossa Senhora, the western slopes of Pico do Concelho, and those of the Pico Branco and Terra Chã complex. It is also commonly found at lower elevations of Serra de Dentro, Barranco Branco, and Lombo de Paredes. Recent *Hystricella* are absent from the southeastern part of the island, i.e., from Vale do Touro, Portela, Pico do Maçarico, and Pico do Baixo. It is likewise not present on the small islets surrounding the main island, namely Ilhéu de Cima, Ilhéu de Baixo, and Ilhéu de Ferro. Subfossil representatives of the genus are found mainly in the mud and aeolinite deposits along the southeastern coast, namely Vale do Touro, Ponta do Passo, Barbinha, and Calhau da Serra de Fora.

**Figure 8.**
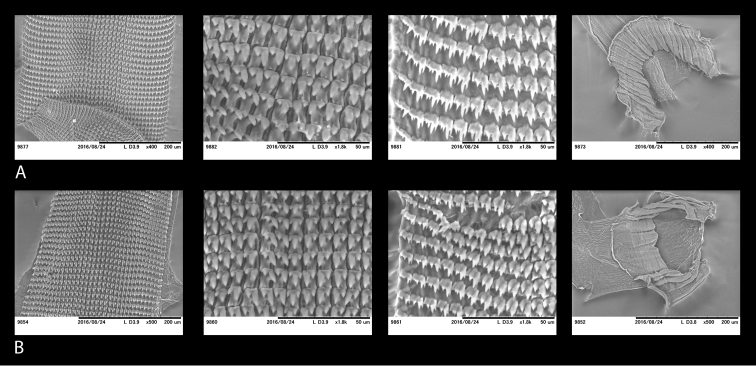
Radulae and jaws of *Hystricella* species. **A** *Hystricella bicarinata*, Ribeira da Areia **B** *Hystricella echinulata*, path to Pico Branco.

##### Ecology.

Representatives of the genus *Hystricella* are commonly found under volcanic rocks scattered on grassland in open fields that are more or less strongly sloping. They have been found under stones in pine woods (Pico do Castelo) or in cracks and crevices of rocky walls (Pico da Gandaia). Disturbed or anthropogenic habitats have also been colonised by representatives of the genus, such as stone walls (Pedregal de Dentro) or terraced areas (southern slopes of Pico do Castelo). Specimens aestivate on the lower surfaces of stones or rocks, frequently forming large clusters of individuals attached to one another, reaching more than 40 to 50 in number. Under a single stone of roughly 60 × 40 cm approximately 200 individuals were counted (southern slopes of Pico do Facho).

##### Nomenclatural and taxonomic remarks.

*Hystricella* was considered a subgenus of *Helix* for a long time (until Wollaston 1878) or was replaced by *Ochthephila* Beck, 1837 (Albers 1850) or *Octephila* Paiva, 1867 (Paiva 1867). Later it was considered as a subgenus of *Discula* R. T. Lowe, 1852 and only since 2002 (Bank et al. 2002) accepted as a distinct genus.

Bank et al. (2002) used the generic name *Geomitra* Swainson, 1840 despite Herrmannsen’s (1847: 470) selection of the nominal species *Helix tiarella* Webb & Berthelot, 1833 as the type species of that genus. The decision of Herrmannsen was followed by all subsequent authors (e.g., Wollaston 1878; Mandahl-Barth 1950; Waldén 1983). The reason to contradict that type selection was the fact that it was wrong with respect to the rules of the International Commission on Zoological Nomenclature’s (ICZN) Code of Zoological Nomenclature (hereafter ‘the Code’) Art. 67.2.1: Swainson introduced *Geomitra* describing and depicting *tiarella*, namely on p. 166 and 332, but he did not mention that name, but instead only *bicarinata*, which therefore would be the type species by monotypy. In consequence *Hystricella* R. T. Lowe, 1855 would become a synonym of *Geomitra* and *Geomitra* *sensu auct*. has to be referred to *Craspedaria* R. T. Lowe, 1852. Nevertheless, this decision was much criticised (Seddon 2008) and a new study of the Code revealed a solution which permits the stabilisation of the former use of *Geomitra* (Groh et al. 2009). Following Art. 70.3 of the Code the combination of the figure of *tiarella* with the name *bicarinata* can be interpreted as a misidentification by Swainson and therefore following Art. 70.3.2, the name *tiarella* becomes available for type selection under the synonymous name *Geomitra bicarinata* Swainson, 1840. This was already recognised by Pfeiffer (1847: 191) who listed Swainson’s combination as a synonym of *Helix tiarella* (cf. Groh et al. 2009).

##### Comparison and comments.

*Hystricella* shows a number of morphological characters that clearly distinguishes it from the other native geomitrid genera from Porto Santo, in particular with regard to the genus *Discula* s. lat., into which the *Hystricella* species were previously placed (Mandahl-Barth 1950; Waldén 1983). On the basis of the shell, the main distinguishing feature of *Hystricella* from *Discula* s. lat. is the continuous and detached peristome. On the contrary, in *Discula* s. lat. and *Callina* (Figs 10–12) the peristome is always interrupted, forming only a thickening or a callous along the parietal region but not a real and proper detached lip. Other minor differences can be found in the ornamentation of the shell’s surface. *Hystricella* always possesses round, somewhat spaced tubercles, whereas *Discula* s. lat. and *Callina* shows mainly elongated, sometimes drop-like tubercles arranged in a regular pattern. In *Hystricella* the size of the tubercles can reach large dimensions in comparison to the overall size of the shell and their size can remarkably vary on the same shell. *Actinella* s. lat. and *Caseolus* s. lat. (see Figs 13–16) also possess an interrupted peristome. Most *Caseolus* species have a more or less granulated shell’s surface and, at first glance, could therefore be confused with *Hystricella*. However, a closer look reveals the different peristome and the overall different shape, with the whorls always rounded and usually without any prominent keel. *Serratorotula* (Fig. 17) has an extremely ornamented shell that is easily distinguishable from *Hystricella*. The shell form of *Heterostoma* is very different from that of *Hystricella* and these two genera are therefore easily distinguishable (see Seddon 2008: 125). Some species of the genus *Spirorbula* sometimes have shells with a continuous peristome with detached lips along the parietal side. The very depressed shell shape and different surface ornamentation of *Spirorbula* however, readily distinguishes the two genera (Fig. 18). *Lemniscia* usually possesses a smooth and rather glossy shell without tubercles or papillae (Fig. 19). The shell morphology of *Wollastonia* gen. n. and its differences with regard to Hystricella will be discussed in the section on Wollastonia gen. n. below.

**Figures 9–19.**
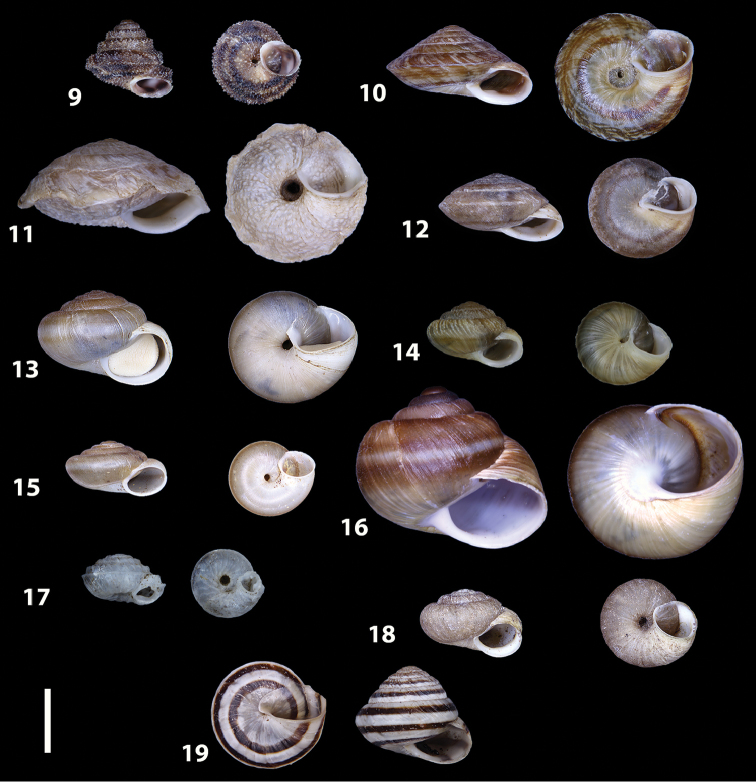
Shells of Portosanctan and Madeiran Geomitridae. **9** *Hystricella bicarinata*, Pico do Castelo, S slope around the Miradouro **10** *Discula discina*, Porto da Morena **11** Discula (Mandahlia) tectiformis, Pico do Baixo **12** *Callina rotula*, Cabeco dos Bades **13** Actinella (Plebecula) littorinella, E of Pico do Castelo **14** Caseolus (C.) innominatus portosanctanus, Ribeira da Areia **15** Caseolus (Helicomela) punctulatus punctulatus, Fonte da Areia **16** Caseolus (Leptostictea) leptosticus, Ponta do Garajau, Madeira **17** *Serratorotula juliformis*, Pico de Ana Ferreira **18** *Spirorbula depauperata*, Pico de Ana Ferreira **19** *Lemniscia michaudi*, Terra Chã. Scale bar: 1 cm.

**Figures 20–30.**
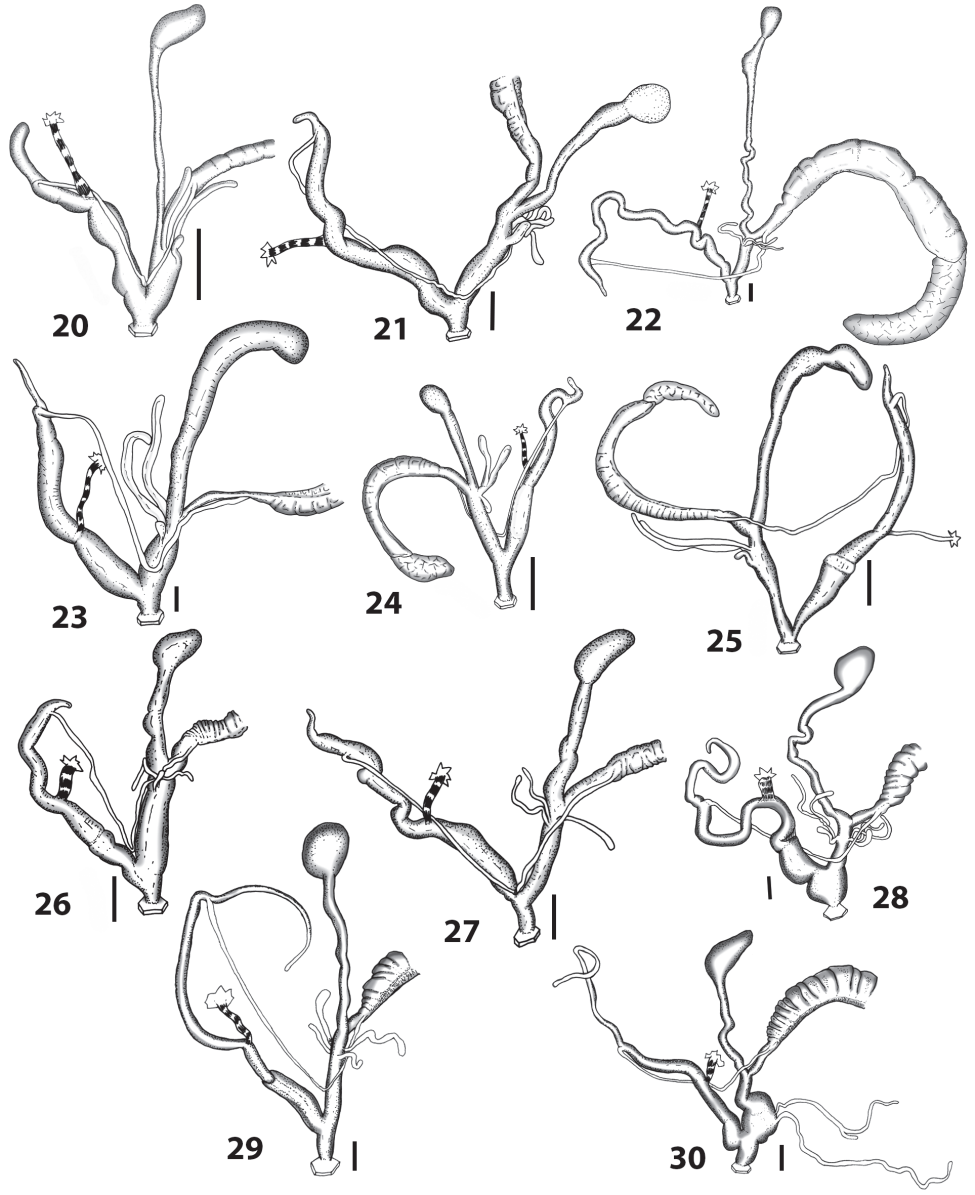
Outer distal genitalia of Portosanctan and Madeiran Geomitridae. 20 *Hystricella bicarinata*, Capela da Graça **21** *Discula polymorpha alleniana*, Porto Moniz, Madeira **22** Discula (Mandahlia) tectiformis, Pico do Baixo **23** *Callina rotula*, Cabeco dos Bades **24** *Serratorotula juliformis*, Pico de Ana Ferreira **25** *Lemniscia michaudi*, Terra Chã **26** Actinella (Plebecula) littorinella E of Pico do Castelo **27** Caseolus (C.) innominatus portosanctanus, Ribeira da Areia **28** Caseolus (Helicomela) punctulatus punctulatus, Fonte da Areia **29** Caseolus (Leptostictea) leptosticus, Ponta do Garajau, Madeira **30** *Spirorbula depauperata*, Pico de Ana Ferreira. Scale bars 1 mm.

With regard to genital morphology, *Hystricella* shows a unique feature that allows the separation of the genus from all native Portosanctan Geomitridae, except from *Wollastonia* gen. n. The main difference is the shape of the penial flagellum which is always short and has a remarkably blunt apex (Fig. 20). *Discula* s. lat. and *Callina*, *Serratorotula*, and *Lemniscia* (Figs 21–25) have a pointed, more or less elongated flagellum. *Hystricella* is easily distinguishable from *Caseolus* s. lat. and *Actinella* s. lat. by virtue of the single vaginal appendages instead of the two that are present in these genera (see Figs 26–29). *Spirorbula* is also easily distinguishable from *Hystricella* because members of this genus possess two extremely long, and sometimes branched, vaginal appendages and a genital atrium with structures on the inner wall consisting of large and partially fringed folds (see Fig. 30).

#### Hystricella bicarinata

Taxon classificationAnimaliaStylommatophoraGeomitridae

(G. B. Sowerby I, 1824)

Figs 31–32 , 33–37 , 38–45 , 46–53 , 54

##### List of synonyms.

1824 *Helix bicarinata* G. B. Sowerby I: 58, pl. 3 fig. 7.

1828 *Helix bicarinata* – Wood: pl. 8 fig. 85.

1831 *Helix duplicata* R. T. Lowe: 58, pl. 6 fig. 30 [unjustified replacement name for *Helix bicarinata* Sowerby I, 1824 [non *Helix C.*[*ochlitoma*] *bicarinata* J. Férussac, 1821, a secondary homonym of *Bulimus bicarinatus* Bruguière, 1792 = *Archachatina bicarinata* (Bruguière, 1792). See Taxonomic Remarks below].

1846 *Helix bicarinata* – L. Pfeiffer: 141, pl. 91 figs 8–11.

1847 *Helix bicarinata* – L. Pfeiffer in L. Pfeiffer 1847–1848: 190.

1854 *Helix bicarinata* – Reeve in Reeve 1851–1854: pl. 142 figs 908–909.

1854 Helix (Ochthephila) bicarinata – Albers: pl. 9 figs 1–4.

1855 Helix (Hystricella) bicarinata – R. T. Lowe: 186.

1867 Helix (Octephila) bicarinata – Paiva: 45.

1878 Helix (Hystricella) bicarinata – Wollaston: 161–163.

1888 *Helix bicarinata* – Tryon in Tryon and [Pilsbry] 1888: 33, pl. 7 fig. 90.

1894 *Geomitra bicarinata* – Pilsbry in Pilsbry 1893–1895: 242.

1931 Geomitra (Actinella) bicarnata – Nobre: fig. 37.

1950 Discula (Hystricella) bicarinata bicarinata – Mandahl-Barth 1950: 31, 55.

1966 Discula (Hystricella) bicarinata – S. G. A. Jaeckel: 53.

1983 Discula (Hystricella) bicarinata bicarinata – Waldén: 267.

2002 *Geomitra bicarinata bicarinata* – Bank et al.: 124.

2008 *Hystricella bicarinata* – Seddon: 79, pl. 29 fig. E, map 178.

2011 *Hystricella bicarinata* – Seddon: e.T6724A12800659.

##### Type material.

**Figures 31–32.**
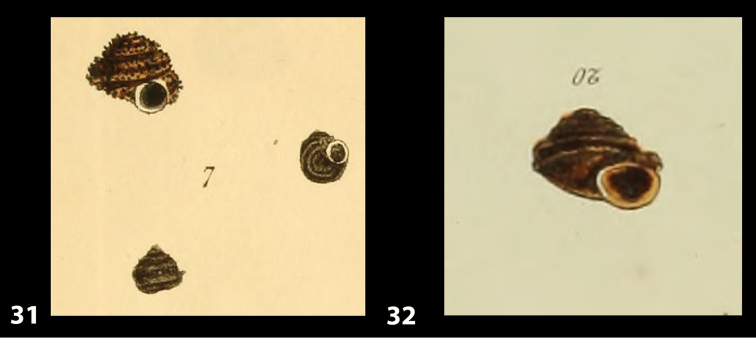
**31** Original figure of *Helix bicarinata* G. B. Sowerby I, 1824 from Sowerby (1824: pl. 3 fig. 7) **32** Original figure of *Helix duplicata* R. T. Lowe, 1831 from R. T. Lowe (1831: pl. 6 fig. 20).

**Figures 33–37.**
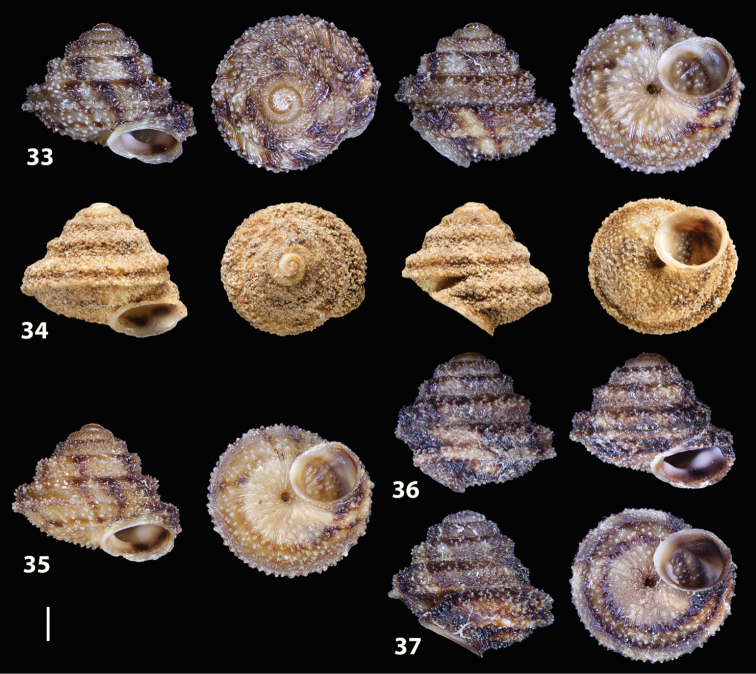
Shells of *Hystricella bicarinata*. **33** neotype of *Hystricella bicarinata*, Pico do Castelo, S slope around the Miradouro, SMF 348936 **34** syntype of *H. duplicata* R. T. Lowe 1831, NHM 1948.7.8.34; Shells of *Hystricella bicarinata* **35** Capela da Graça **36** Pico do Castelo, S slope around the Miradouro **37** Pico da Gandaia. Scale bar 1 mm.

Despite intensive research in multiple museum collections (NHM, NMW, MMUE, ANSP, NHC, NMS, OUMNH, RAM, SMF) that could have held the type material of the taxon, no such material could be traced and therefore we deem it reasonable to assume that the type material is lost. To stabilise the present interpretation of *Helix bicarinata* G. B. Sowerby I, 1824 and to clarify its taxonomic status we designate a neotype here, which is deposited in the collection of the SMF under the No. SMF 348936 (see Fig. 33). The original figures of *Helix bicarinata* G. B. Sowerby I, 1824 from Sowerby (1824: pl. 3 fig. 7) 32 and *Helix duplicata* R. T. Lowe, 1831 from R. T. Lowe (1831: pl. 6 fig. 20) are depicted in Figs 31–32. The neotype (Fig. 33) is consistent with the figure in Sowerby (1824: pl. 3 fig. 7) and the original description (see below), especially with regard to size, shape and the presence of two keels on the body whorl. The taxon was originally described from Porto Santo without more detailed locality data, which is consistent with origin of the specimen selected as neotype.

##### Locus typicus.

Pico do Castelo, S slope around the Miradouro, under stones in pine wood, 33°04'42"N/16°20'00"W, 270 m (through the designation of the neotype).

##### Further material examined.

All from Porto Santo, ANSP H 20185/13, NW slope of Pico Branco, 33°05'50"N/16°18'57"W to 33°05'52"N/16°18'49"W, 150–200 m, leg. J. & C. Hemmen, Jan. 8 1981; ANSP H 11776/2, NW slope of Pico de Juliana, 33°05'34"N/16°19'25"W, up to 450 m, leg. J. & C. Hemmen, Jan. 6 1981; CKG/12, Pico de Juliana, under stones, 33°05'31"N/16°19'20"W, 365 m, leg. K. Groh, Jun. 23 1983; ANSP H 11788/11, CMN/3, SW slope of Pico do Juliana, 33°05'30"N/16°19'23"W, 360 m, leg. J. Gerber, K. Groh & J. Hemmen, Aug. 13 1985; CFW 11149/1, Pico Juliana, old quarry on the southern slope, 33°05'28"N/16°19'17"W, 330 m, leg. F. Walther & E. M. Gryl, Apr. 3 2017; ANSP H 11769/3, Ribeiro das Esmoitadas (E Camacha), 33°05'27"N/16°20'17"W, 130 m, leg. J. Gerber, K. Groh & J. Hemmen, Aug. 13 1985; CKG/2, SW of Pico de Juliana, 33°05'14"N/16°19'32"W, 360 m, leg. K. & C. Groh, Oct. 27 1980; CFW 11158/10, Pico do Facho, NW slope, 33°05'12"N, 16°19'38"W, 370 m, leg. F. Walther, Apr. 3 2017; ANSP H 20186/1, NE slope of Pico do Facho, 33°05'07"N/16°19'23"W, 430–450 m, leg. K. Groh & J. Hemmen, Jun. 28 1983; CWDM/6, CMN/ 3, ridge between Pico do Facho and Pico da Gandaia, stone walls, 33°05'04"N/16°19'21"W, 460 m, leg. W. De Mattia & J. Macor, May 25 2015; CWDM/15, N slope of Pico do Facho in *Tamarix* wood, under stones, 33°05'04"N/16°19'25"W, 475 m, leg. W. De Mattia & J. Macor, May 25 2015; CKG/44, CMN/6, W of Pico do Facho, under stones, 33°05'04"N/16°19'33"W, 450 m, leg. K. Groh, Jun. 29 1983; ANSP H 11778/c. 40, SW slope of Pico do Facho, 33°05'03"N/16°19'31"W, 460–480 m, leg. K. Groh & J. Hemmen, Jun. 28 1983; CKG/24, CMN/44, upper Ribeira Formosa between Covau and Serra de Fora, under stones, 33°05'02"N/16°19'14"W, 140 m, leg. K. Groh, Jun. 19 1983; ANSP H 11775/13, W slope of Pico do Facho, 33°05'02"N/16°19'38"W, leg. J. & C. Hemmen, Jan. 6 1981; ANSP H 11834/19 [sub *H. echinulata*], summit of Pico da Gandaia, 33°05'01"N/16°19'14"W, 440 m, leg. J. & C. Hemmen, Jan. 6 1981; CWDM/11, CMN/7, Pico da Gandaia, southern rocky cliffs close to the top, under stones, 33°05'01"N/16°19'14"W, 440 m, leg. W. De Mattia & J. Macor, May 25 2015; ANSP H 11779/24, SW slope of Pico do Facho, 33°04'56"N/16°19'39"W to 33°05'02"N/16°19'30"W, 300–450 m, leg. K. Groh & J. Hemmen, Jul. 1 1983; CWDM/15, CMN/3, N and E slopes and summit of Pico do Castelo, under stones in pine wood, 33°04'55"N/16°20'07"W, 350 m, leg. W. De Mattia & J. Macor, May 22 2015; CWDM/25, CMN/5, S slope of Pico do Facho, along path at the S border of the pine wood, under stones, 33°04'52"N/16°19'26"W, 355 m, leg. W. De Mattia & J. Macor, May 17 2015; CKG/8, Pico do Castelo, S slope around the Miradouro, under stones in pine wood, 33°04'51"N/16°20'03"W, 420 m, leg. K. & C. Groh, Oct. 27 1980; CWDM/22, CMN/4, Ribeira da Areia, serpentine 240 m NNW the quarry, under stones, 33°04'51"N/16°18'41"W, 140 m, leg. W. De Mattia & J. Macor, May 21 2014; ANSP H 11787/c. 30, approx. 1 km NE Capela da Graça, 33°04'49"N/16°19'04"W, 250 m, leg. J. & C. Hemmen, Jan. 4 1981; CWDM/12, CMN/4, lake shore S of Ribeira da Serra de Dentro, under stones and in stone walls, 33°04'48"N/16°18'31"W, 70 m, leg. W. De Mattia & J. Macor, May 22 2015; ANSP H 20187/1, NW slope of Pico do Concelho, 33°04'44"N/16°18'04"W to 33°04'43"N/16°18'01"W, 250–300 m, leg. K. Groh & J. Hemmen, Jul. 29 1983; CKG/8, CMN/8, Pico do Concelho, NW of the top, under stones, 33°04'43"N/16°17'59"W, 260 m, leg. K. Groh, Jun. 29 1983; ANSP H 11835/12 [sub *H. echinulata*], NW slope of Pico do Concelho, 33°04'43"N/16°18'01"W, approx. 280 m, leg. J. & C. Hemmen, Jan. 6 1981; ANSP H 12780/32, NW slope of Pico do Concelho, 33°04'43"N/16°18'01"W, 250–300 m, leg. K. Groh & J. Hemmen, Jun. 6 1983; CWDM/15, CMN/2, summit of Pico do Concelho, under stones, 33°04'42"N/16°18'01"W, 280 m, leg. W. De Mattia & J. Macor, May 18 2015; CWDM/18,CMN/4, Pico do Castelo, S slope around the Miradouro, under stones in pine wood, 33°04'42"N/16°20'00"W, 270 m, leg. W. De Mattia & J. Macor, Feb. 12 2012; CMN/1, Pico do Castelo, S slope around the Miradouro, under stones in pine wood, N slope, 33°04'42"N/16°20'01"W to 33°04'51"N/16°20'03"W, 300–430 m, leg. K. Groh Jul. 1 1983; ANSP H 11783/24, Pico do Castelo, 33°04'42"N/16°20'01"W to 33°04'51"N/16°20'03"W, 350–430 m, leg. K. Groh & J. Hemmen, Jul. 1 1983; CWDM/8, CMN/4, path from Capela da Graça to Pico do Castelo along the Levada, under stones, 33°04'38"N/16°19'48"W, 165 m, leg. W. De Mattia & J. Macor, May 17 2015; CKG/3, CMN/68, Capela da Graça up to Pico de Facho-Motos de Fora, under stones, 33°04'38"N/16°19'33"W, 200 m, leg. K. Groh, Jun. 23 1983; ANSP H 11785/9 spms, Ribeiro do Formoso, 33°04'36"N/16°19'04"W, 130 m, leg. J. Gerber, K. Groh & J. Hemmen, Aug. 13 1985; ANSP H 11777/12, approx. 1 km S Serra de Dentro, 33°04'33"N/16°18'25"W, 160 m, leg. J. & C. Hemmen, Jan. 8 1981; ANSP H 11782/c. 30, slope between the SE slope of Pico do Castelo and Capela da Graça, 33°04'32"N/16°19'38"W, 160 m, leg. J. & C. Hemmen, Jan. 4 1981; CFW 11161/7, Casinhas, N of Capela da Graça, 33°04'27"N/16°19'27"W, 150 m, leg. F. Walther, Apr. 4 2017; CKG/11, CMN/1, Pico do Baixo, 33°04'26"N/16°19'44"W to 33°04'46"N/16°19'44"W, 100–250 m, leg. K. Groh, Jul. 11, 1983; CWDM/23, Engula, under stones, 33°04'19"N/16°19'01"W, 150 m, leg. W. De Mattia & J. Macor, May 18 2015; CFW 11159/>10, E slope of Rocha de Nossa Senhora, 33°04'19"N/16°19'01"W, 150 m, leg. F. Walther, Apr. 3 2017 ANSP H 11771/18, N of Portela (E of Vila Baleira), 33°04'07"N/16°19'17"W, 120 m, leg. J. & C. Hemmen, Jan. 4 1981; CKG/2, CMN/1, Capela da Graça up to Pico do Facho-Motos de Fora, N slope, 33°04'38"N/16°19'33"W to 33°05'04"N/16°19'33"W, approx. 200–450 m, leg. J. Gerber, K. Groh & J. Hemmen, Jun. 28 1985; ZMH 120610/2, foot of Pico do Castelo, c. 33°04'37"N/16°19'49"W, 170 m, ex coll. W. Fauer, leg. J. & C. Hemmen, Jul. 11 1983; ZMH 110165/68, slopes of Pico do Facho, under stones; c. 33°04'59"N/16°19'25"W, 430 m a.s.l., leg. E. Clauss, Sep. 22 1992; ZMH 24288/2, Madeira Archipelago, without exact locality data, ex coll. Altonaer Museum; ZMH, 24289/1, Porto Santo, without exact locality data, ex coll. Museum Klagenfurt; ZMH 24290/3, Porto Santo, without exact locality data, ex coll. Altonaer Museum, ex coll. O. Semper, ex coll. Dohrn.

##### Original description.

From Sowerby (1824): testa subglobosa, spira breviuscula, subconica; anfractibus quinque quadratis, mediane carinis duabus, superiore obtusiuscula: apertura integra, rotunda, peristomate distincto: umbilico parvo. Axis ^3^∕_16_, diam. ¼ unc.

##### Diagnostic features of the shell.

Shell as in the genus description. The main diagnostic feature is the presence of two well-developed keels along the penultimate and body whorl. The lower keel is usually slightly stronger and more evident than the upper one along both whorls. The overall shape of the shell of *Hystricella bicarinata* is always conical and scalariform by virtue of a “shoulder” that lets the contour of the whorls appear markedly angular. The sutures are deep and well-marked (see Figs 33–37).

##### Measurements.

D 4.9 ± 0.2 mm (range 4.4–5.3 mm); H 3.8 ± 0.2 mm (range 3.4–4.2 mm); FW 2.4 ± 0.2 mm; PA 53.5 ± 2.4°; DU 0.4 ± 0.09 mm; NT 21 ± 6; NW 5.6 ± 0.1 (range 5.1–5.75) (*n* = 40). Ratio D/H 1.3; ratio FW/H 0.6.

##### Body.

Body as in the genus description.

##### Genital anatomy.

**Figures 38–45.**
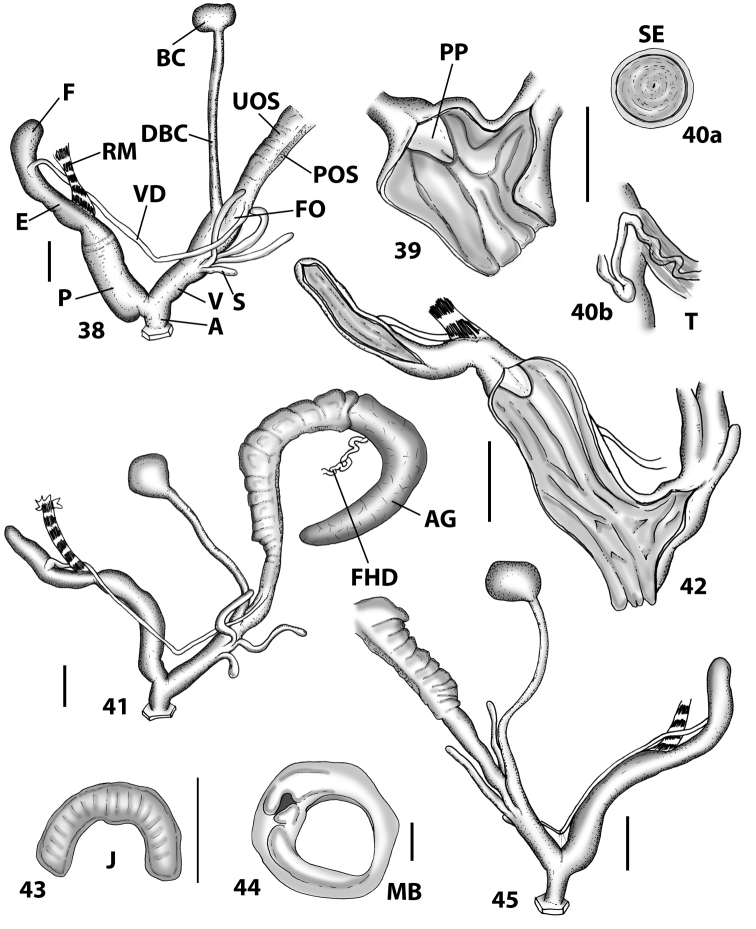
Genitalia and anatomy of *Hystricella bicarinata*. Pico do Castelo, S slope around the Miradouro: **38** whole genitalia excluding part of OSD, AG and gonads **39** ornamentation of the inner walls of the distal penis, the vagina and the genital atrium **40** transverse section of penial papilla. Pico da Gandaia: **41** whole genitalia excluding gonads **42** ornamentation of the inner walls of the flagellum, the penial complex, the vagina and the genital atrium. Ribeira de Serra de Dentro: **43** jaw. Pico da Gandaia: **44** mantle border. Ribeira de Serra de Dentro: **45** whole genitalia excluding part of OSD, AG and gonads. Scale bars 1 mm.

**Figures 46–53.**
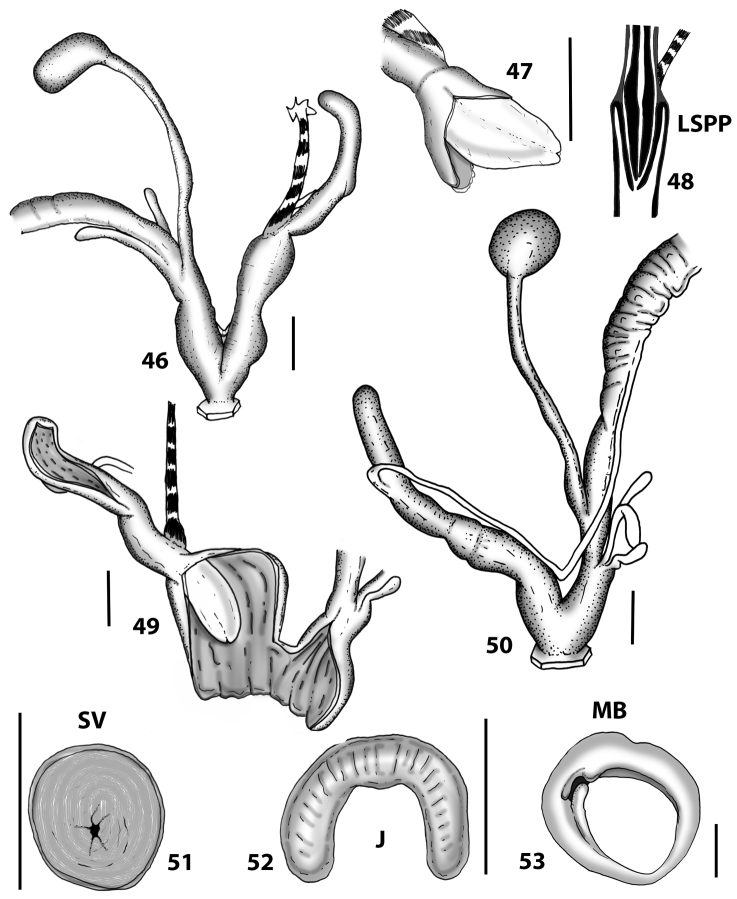
Genitalia and anatomy of *Hystricella bicarinata*. Capela de Graça: **46** whole genitalia excluding part of OSD, AG and gonads **47** penial papilla **48** longitudinal section of the penial papilla **49** ornamentation of the inner walls of the flagellum, the penial complex, the vagina and the genital atrium. Ridge between Pico do Facho and Pico da Gandaia: **50** whole genitalia excluding part of OSD, AG and gonads. NE slopes of Pico do Castelo: **51** section of vagina. Capela da Graça: **52** jaw. S slopes Pico do Facho: **53** mantle border. Scale bars 1 mm.

As in the genus description. The albumen gland is long and connected to an equally long sperm-oviduct. The prostatic part extends into a thin vas deferens that is twice as long as the sperm-oviduct. The free oviduct is somewhat variable in length, but is usually three to four times shorter than the vagina. The duct of the bursa copulatrix is usually wide, slightly shorter than the penis, opening out into a roundish, small bursa copulatrix. One tuft of digitiform glands consisting of two, more rarely three never branching glands. The inner ornamentation of the vagina consists of very wide, low, and smooth pleats running longitudinally as far as the genital atrium. The penial flagellum is short, remarkably cylindrical and with a blunt apex. It is usually as long as the epiphallus. Its internal walls are completely smooth. The epiphallus is short and its internal walls are smooth. The penis lacks any muscular or glandular sheath. It is thick-walled and approximately four times longer than the flagellum. It is cylindrical and sometimes slightly swollen in its distal part. The inner walls of the penis are usually smooth or with very smooth, little elevated and spaced pleats which run longitudinally and reach the genital atrium (see Figs 38–53).

##### Distribution.

**Figure 54.**
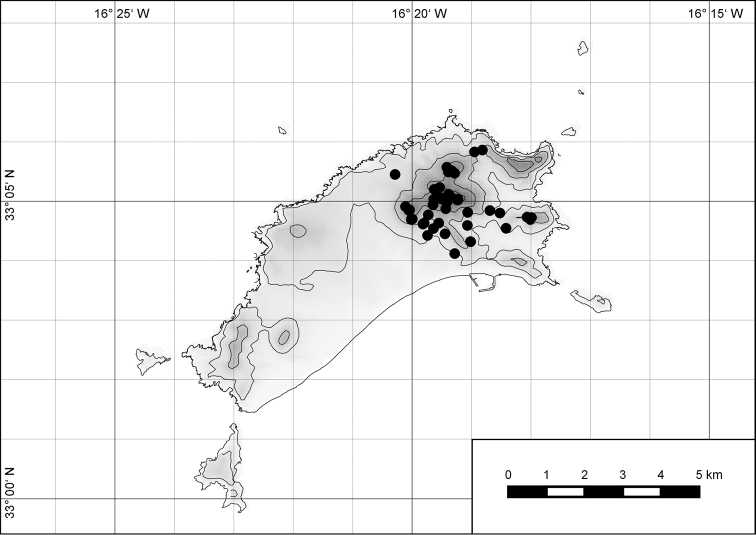
Distribution of *Hystricella bicarinata*.

*Hystricella bicarinata* is endemic to the island of Porto Santo (Madeiran Archipelago, Portugal) (Fig. 54). The species is restricted to the eastern, mountainous part of the island and is present only in the central section of this area. It is commonly found on the slopes of Pico do Castelo, Pico do Facho, Pico de Juliana, Pico da Gandaia, Rocha de Nossa Senhora, and the western slopes of Pico do Concelho. It is also commonly found at lower elevations of the Serra de Dentro, Barranco Branco, and Lombo de Paredes. It is not present on the small islets surrounding the main island, namely Ilhéu de Cima, Ilhéu de Baixo, and Ilhéu de Ferro. Subfossil representatives of *Hystricella bicarinata* are not known. Seddon (2008: 181) depicts the distribution of *H. bicarinata*. The four southernmost points indicate the presence of the species also for the southern ridge of eastern Porto Santo. We here infer that these localities are based upon misidentifications with other species, probably some representatives of *Wollastonia*, i.e., *W. jessicae* sp. n. or *W. klausgrohi* sp. n.

##### Ecology.

*Hystricella bicarinata* is commonly found under volcanic rocks scattered on grassland in open fields that are more or less sloping. It has also been found under stones in pine woods (Pico do Castelo) or in cracks and crevices on rocky walls (Pico da Gandaia) or terraced areas (southern slopes of Pico do Castelo). The specimens aestivate on the lower surfaces of stones or rocks, frequently forming large clusters of 40 to 50 individuals attached one to another. Under a single stone of roughly 60 × 40 cm approximately 200 individuals were observed (southern slopes of Pico do Facho).

##### Comparison and comments.

The presence of two keels along the body whorl easily distinguishes *H. bicarinata* from *H. echinulata*. Where both taxa come into contact in the northern to northeastern part of Porto Santo (Fig. 4) intermediate populations exist however, suggesting a possible hybrid zone (see below).

At first glance, and due to the small dimensions, *H. bicarinata* can be confused with the fossil species *H. aucta* and *H. microcarinata* sp. n. (see Fig. 74). However, a closer analysis reveals major differences in many features of the shell. *Hystricella aucta* has a more solid shell with two, almost equally developed keels along the body whorl. The penultimate and the third whorl have a single strong upper keel only. The tubercles in *H. aucta* are much smaller and finer than in *H. bicarinata* (see Figs 71–73).

*Hystricella microcarinata* sp. n. resembles *H. bicarinata* with regard to dimensions and overall shape; however, the weak keels along the penultimate and body whorl and the well-developed and rather elevated growth lines allow the separation of the two species. Moreover, the tubercles in *H. microcarinata* sp. n. are somewhat smaller and more scattered.

*Hystricella bicarinata* can also resemble some of the species included in the newly described genus *Wollastonia* gen. n. Species such as *W. turricula*, *W. leackociana* and *W. oxytropis* differ in the very fine granulation present on the shell’s surface. These species do not possess evident tubercles but only small granules that are remarkably smaller in dimension than the tubercles of *H. bicarinata*. Moreover, the overall shape of all three above-mentioned species is different from that of *H. bicarinata* (see e.g. Figs 100, 119, 130). Other (sub-) species assigned to *Wollastonia* gen. n. such as *W. vermetiformis*, *W. jessicae jessicae* sp. n., *W. jessicae monticola* ssp. n., and *W. klausgrohi* sp. n. (see Figs 118–120, 147–149, 159–161, 168–170) are somewhat similar to and could be confused with *H. bicarinata*. These taxa can be readily distinguished, however, by their more scalariform shape, somewhat stronger keels, and the sparser arrangement of the tubercles. Moreover, the distributional ranges of *Hystricella* and *Wollastonia* gen. n. do not significantly overlap and molecular data strongly supports differentiation at the genus-level (thus also at species level).

##### Nomenclatural and taxonomic remarks.

We report below a statement sent to us by Ruud Bank referring to the erroneously introduced name *Helix duplicata* R. T. Lowe, 1831. “The name *Helix* [*Cochlitoma*] *bicarinata* A. Férussac, 1821 does not exist! Férussac simply reclassified *Bulimus bicarinatus* Bruguière, 1792 under Helix (Cochlitoma). Thus, *Helix bicarinata* G. B. Sowerby I, 1824 was at that time (and only for a very short period of time) a secondary homonym of *Helix bicarinata* (Bruguière, 1792). *Helix duplicata* R. T. Lowe, 1831 was introduced to replace the name of Sowerby due to homonymy with the Férussac name, but as stated above, Férussac did not introduce such a name, it was simply a generic change for *Bulimus bicarinatus* Bruguière. In my opinion, the name *duplicata* cannot be considered a nomen novum. It would have been only a nomen novum when R. T. Lowe mentioned Bruguière as the author of the senior taxon – what he didn’t.

Mandahl-Barth (1950), Jaeckel (1966) and Waldén (1983) placed this taxon in *Discula* and considered *Hystricella* as a subgenus. Bank et al. (2002) considered the species to belong to a distinct genus (under the name *Geomitra*). Only recently Seddon (2008) appointed this species to the genus *Hystricella* and emphasised anatomical differences with the genus *Discula*.

##### Status and conservation.

The opinion of Seddon (2011a) “[*Hystricella bicarinata*] has a total extent of occurrence of 10 km^2^ but is present over the eastern part of the island and in these sites it is abundant” has been confirmed by our field researches. For this reason, we think that the assessment as Near Threatened (NT) is appropriate at present.

### Putative hybrid populations of *H. bicarinata* and *H. echinulata*

**Material examined.** All from Porto Santo, CWDM/8, Porto de Pedregal, under stones. 33°06'14"N/16°19'18"W, 20 m, leg. W. De Mattia & J. Macor, May 2014 and May 2015; CWDM/22, CKG/3, CMN/8, SMF 348933/4, NMW Z.2016.013.00005/1 and NMW Z.2016.013.00006/19, Cebecos dos Bades at confluence of two temporary creeks, under stones, 33°06'09"N/16°19'25"W, 34 m, leg. W. De Mattia & J. Macor, May 19 2015; CFW/11147/>10, Ribeira do Pedregal, lower part of the valley near confluence with the tributaries, 33°06'08"N/16°19'32"W, 50 m, leg. F. Walther & E.M. Gryl, Apr. 2 2017; CFW 11150/>10, Ribeiro do Pedregal, upper part, downstream of the abandoned houses, 33°05'57"N/16°19'32"W, 120 m, leg. F. Walther, Apr. 2 2017; CWDM/15, Pico Branco, Faja Pequena at the beginning of the path to Terra Chã, under stones, 33°05'50"N/16°18'44"W, 180 m, leg. W. De Mattia & J. Macor, May 2014; CWDM/10, CKG/1, CMN/5, Pedregal de Dentro near abandoned houses, under stones, 33°05'46"N/16°19'27"W, 195 m, leg. W. De Mattia & J. Macor, May 2015; CWDM/16, Pico de Cabrita, northern slope close to the road, under stones, 33°05'42"N/16°19'23"W, 240 m, leg. W. De Mattia & J. Macor, May 2014; CWDM/11, CMN/5, path to Pico Branco, terraced S slope of Pico Branco, under stones. 33°05'35"N/16°18'25"W, 270 m, leg. W. De Mattia & J. Macor, May 2015.

**Figures 55–70.**
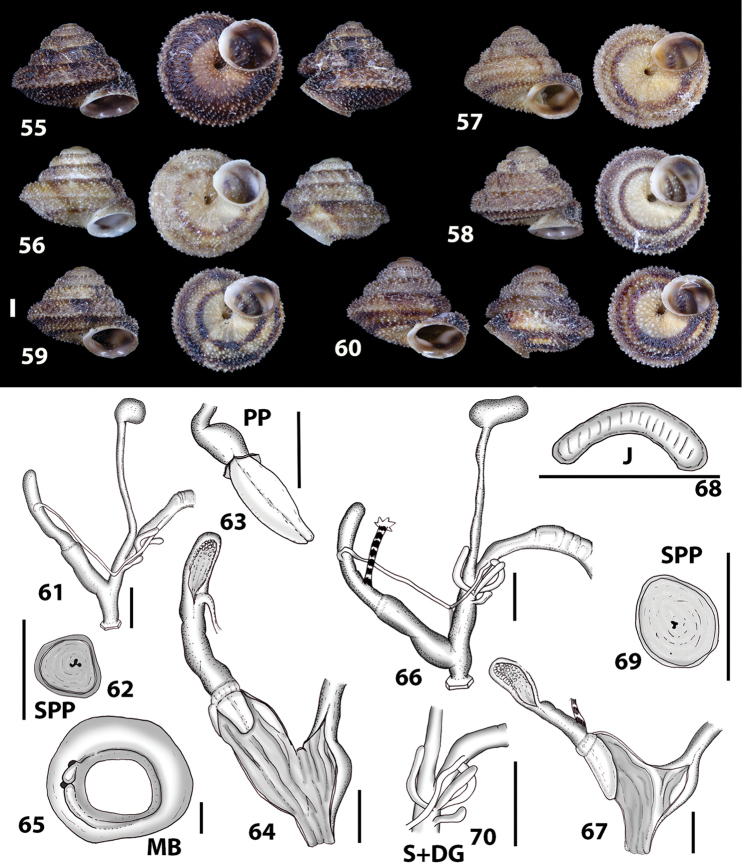
Shells, anatomy and genitalia of the putative hybrid populations of *H. bicarinata* and *H. echinulata*. Shells: **55** Pedregal de Dentro **56** Ribeira Formosa **57** Pico de Cabrita **58–59** Cabecos dos Bades **60** Pedregal de Fora. Genitalia and anatomy. Cabecos dos Bades: **61** whole genitalia excluding part of OSD, AG and gonads **62** section of penial papilla **63** penial papilla **64** inner walls of atrium and penis **65** mantle border. Pedregal de Dentro: **66** whole genitalia excluding part of OSD, AG and gonads **67** inner walls of atrium and penis **68** jaw **69** section of penial papilla **70** vaginal glands and vaginal appendage. Scale bars 1 mm.

**Comments.** The genetic analyses revealed an area in the northeastern part of Porto Santo where both, haplotypes assigned to *H. bicarinata* and *H. echinulata* are present (Fig. 5). Some of the specimens are morphologically closer to *H. echinulata* with regard to the shell, but possess a *H. bicarinata* mitochondrial haplotype or vice versa. Some specimens may also be conchologically intermediate. Investigation of the genital system showed that much of the genital anatomy is, in fact, identical with that of either *H. bicarinata* or *H. echinulata*. However, numerous small papillae that cover the inner walls of the tip of the penial flagellum were observed in several specimens that are lacking in *H. bicarinata* or *H. echinulata* (Figs 55–70). Sometimes these papillae can slightly extend distally, towards the vas deferens opening, but never exceed to more than one third of the total flagellum length. Populations that exhibit these features usually occur at lower altitudes compared to *H. echinulata* or *H. bicarinata*, from almost sea level (Porto de Pedregal) to 240 m a.s.l. across the southern slope of Pico Branco and are limited to the northeastern part of the island, where they are distributed along the southern slopes of the Pico Branco massif, Lombo Celado, Cabecos dos Bades with Porto de Pedregal, Pedregal de Dentro, and the northern slopes of Pico de Cabrita. Because of the presence of mitochondrial haplotypes from both *H. bicarinata* and *H. echinulata*, these populations are here tentatively interpreted as hybrid populations. Further investigations that allow an estimation of gene flow among these populations will, however, be necessary to decide on their definite status. In case that these populations really are of hybrid origin, the specimens with papillae in the penial flagellum may represent an example of transgressive segregation.

### 

#### † Hystricella aucta

Taxon classificationAnimaliaStylommatophoraGeomitridae

(Wollaston, 1878) stat. n.

Figs 71–73 , 75

##### List of synonyms.

1867 Helix (Octephila) vermetiformis var. α minor Paiva: 48 [non *Helix minor* mult. auct.].

1878 Helix (Hystricella) bicarinata var. ß aucta Wollaston: 162–163.

1950 Discula (Hystricella) bicarinata aucta – Mandahl-Barth: 31, 55.

1983 Discula (Hystricella) bicarinata aucta – Waldén: 267.

2002 *Geomitra bicarinata aucta* – Bank et al.: 124.

2006 *Discula bicarinata aucta* – Cameron et al.: 31.

2008 *Hystricella bicarinata aucta* – Seddon: 79.

2009 *Hystricella bicarinata aucta* – Groh et al.: 21 fig. 25.

##### Type material.

**Figures 71–74.**
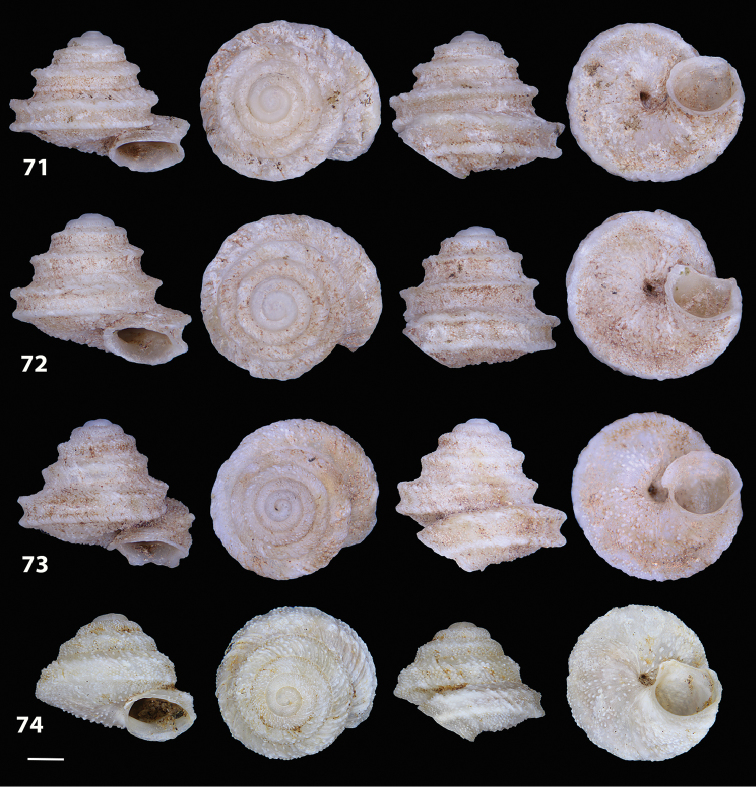
Shells of *Hystricella aucta*. **71** neotype, SMF 348937 from Barbinha **72** Barbinha **73** Ponta da Canaveira **74** *Hystricella microcarinata* sp. n., holotype, SMF 348924. Scale bar: 1 mm.

Despite intensive research in multiple museum collections (NHM, NMW, MMUE, ANSP, NHC, NMS, RAM, SMF) that could have held the type material of the taxon, no such material could be traced and therefore we deem it reasonable to assume that the type material is lost. To stabilise the present interpretation of Helix (Hystricella) bicarinata var. ß aucta Wollaston, 1878 and to clarify its taxonomic status we designate a neotype here, which is deposited in the collection of the SMF, under the No. SMF 348937 (see Fig. 71). The neotype is consistent with the original description (see below), especially with regard to size, shape and the presence and development of the two keels on the body whorl. The taxon was originally described from Porto Santo, which is consistent with the origin of the specimen selected as neotype.

##### Loci typici.

[*aucta*] Barbinha, Quaternary slope deposits, "grey layer", 33°04'04"N/16°17'49"W, 8 m, (through the designation of the neotype); [var. minor]: … rara ad Zimbral d'Aréa.

##### Further material examined.

All from Porto Santo, CKG/1, S slope of Pico do Concelho, Quaternary slope deposit, 33°04'35"N/16°18'03"W, 200–230 m, leg. K. & C. Groh, Jun. 29 1980; CKG/2, Barbinha, Quaternary slope deposits, "grey layer", 33°04'04"N/16°17'49"W, 8 m, leg. K. & C. Groh & J. & C. Hemmen, Jul. 4 1983; ANSP H 11789/9 [sub *H. bicarinata*], Barbinha, Quaternary slope deposits, "red layer", 33°04'04"N/16°17'49"W, 8 m, leg. J. Gerber, K. Groh & J. Hemmen, Aug. 12 1985. CKG/1, first bay S of Barbinha, Quaternary slope deposit, 33°03'51"N/16°17'46"W, 18 m, leg. K. & C. Groh & J. & C. Hemmen, Jul. 5 1983. CGK/3, Ponta da Canaveira, Quaternary aeolinites, 33°02'30"N/16°23'35"W, 50 m, Jun. 24 1983, leg. K. Groh & J. Hemmen; CGK/3, S of Ponta da Canaveira, Quaternary aeolinites, 33°02'30"N 16°23'34"W, 55 m, Oct. 26 1980, leg. K. Groh & J. Hemmen; CKG/2, Vale do Touro, Quaternary slope deposit cut by water erosion in the banks of the valley, 33°03'48"N/16°19'17"W, 15 m, leg. K. & C. Groh, Aug. 16 1980; CWDM/14, S slope of Vale do Touro hill, 50 m W of the oil tanks, fossils beds in mixed gravel and mud deposits, 33°03'47"N/16°19'26"W, 15 m, leg. W. De Mattia & J. Macor, May 2015; CFW 12172/1 fragm. [cf. *aucta*], Ponta da Galé, E end of tunnel, lower level [of slope deposits], coarse, red coloured gravel with large stones, 33°03'44"N/16°17'45"W, 30 m, leg. F. Walther, Apr. 4 2017; CFW 12173/16, CKG/3, S of Ponta da Canaveira, (sub-)fossil [slope-]deposits, coarse, red coloured gravel, 33°02'25"N/16°23'41"W, 30 m, leg. F. Walther, Apr. 2 2017; CFW 12175/8, E of Vila Baleira, end of Vale do Touro, (sub-)fossil [slope-] deposits, coarse, black gravel, 33°03'48"N/16°19'15"W, 20 m, leg. F. Walther, Apr. 5 2017; CFW 12174/13, CKG/2, E of Vila Baleira, S slope of the hill above Vale do Touro, W of the oil tanks, [(sub-)fossil slope-deposits of] red gravel, 33°03'47"N/16°19'26"W, 25 m, leg. F. Walther, Apr. 5 2017.

##### Original descriptions.

[*aucta*]: From Wollaston 1878: There is however an appreciably larger form of this species [*bicarinata*] (cited in the present catalogue as the 'var. ß. *Aucta*’) to which the subfossil examples might perhaps be better referred, – in which the upper (or medial) keel is a trifle more horizontal and prominent, and the shell is full 3 lines (instead of only approximately 2½) across its broadest part – which was found in Porto Santo by Mr. Watson, and which I have received from him as the 'recent state of the *H. vermetiformis*, Lowe.' the 'var. ß. *Aucta*' of the *H. bicarinata* in its very much larger size, and in its volutions (the ultimate one of which is not quite so deflected at the aperture) being 7 in number, instead of only 6 or 6½; [var. minor]: From Paiva 1867: Variet. adest α *minor*, testa minore.

##### Redescription of the shell.

Shell small for the genus, with 5.7 regularly increasing whorls, the protoconch with 1.9 whorls. The form of the shell is conical, the concave teleoconch whorls, with two prominent keels situated at the upper ⅐, respectively ⅖ of the total height of the last whorl. The last whorl measures 62%, the penultimate whorl 19% of the total shell height. The lower 60% of the body whorl beneath the second keel are rather straight, only a little convex in the middle. The suture is simple, only slightly sunken beneath the second keel of the preceding whorl. The aperture, which is inclined to the vertical axis of the shell in an angle of 49° and is descending in the last 5% of the last whorl in an angle of 38° to the horizontal axis, has an elliptical-ovate form, its width is 42% of the total shell width and its height 27% of the total shell height. It has an only slightly reflected lip, which is completely detached from the body whorl. The eccentric umbilicus, which is approximately 12% as wide as the shell, is in the upper whorls needle stitch-like. The protoconch is smooth. The teleoconch is equipped with few oblique radial ribs, seven in the penultimate quadrant of the body whorl and is additionally covered by numerous coarse tubercles. The number of tubercles in the standard quadrate of the base is 31; the tubercles are of approximately equal size. There are no traces of colouration (Figs 71–73).

##### Measurements.

D 5.5 ± 0.5 mm (range 5.1–6.4 mm); H 4.6 ± 0.4 mm (range 4.1–5.3 mm); FW 2.8 ± 0.2 mm; PA 51 ± 2.1; DU 0.5 ± 0.03 mm; NT 18 ± 6; NW 5.5 ± 0.4 (*n* = 22). Ratio D/H 1.2; ratio FW/H 0.6.

##### Distribution.

**Figure 75.**
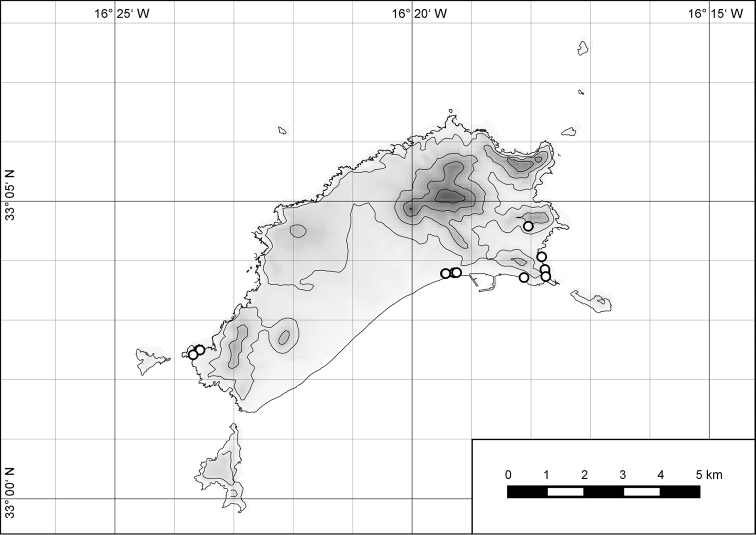
Distribution of *Hystricella aucta*.

*Hystricella aucta* is known only from the southeastern coast of Porto Santo: from the hill immediately east of Vila Baleira (mud and slope deposits at Vale do Touro) to the mud deposits and aeolinites along the southeastern coast (Barbinha, Zimbral da Areia, and Porto dos Frades) (Fig. 75).

##### Comparison and comments.

Because of its small size, the species can only be confused with the similar sized recent *H. bicarinata* which, however, is somewhat larger on average, has less developed keels, especially the upper one is less prominent and has coarser and denser tubercles on its shell surface, a narrower umbilicus and a more distinctly descending aperture. The smaller *H. microcarinata* sp. n., which has a much more rounded globular form, bears less prominent keels on the whorls, has finer and more densely arranged tubercles, has a much narrower umbilicus and a different shape as well as differently positioned angles of the aperture. The similar sized *Wollastonia beckmanni* sp. n., which has a much flatter shape, has no keels on the whorls, a much wider umbilicus, much more and finer tubercles and differently positioned angles of the aperture.

##### Nomenclatural and taxonomic remarks.

Considered by Wollaston (1878) as the fossil variation of *H. bicarinata*. We here consider it as a distinct species because of the shell differences. Like *H. bicarinata*, *H. aucta* has previously (Mandahl-Barth 1950, Waldén 1983, Bank et al. 2002) been listed as belonging to *Discula* and later on to *Geomitra*.

##### Status and conservation.

Extinct before the islands’ scientific exploration in the 19^th^ century, possibly already before human settlement.

#### † Hystricella microcarinata

Taxon classificationAnimaliaStylommatophoraGeomitridae

De Mattia & Groh sp. n.

http://zoobank.org/EE848F87-CEBC-4430-9D7F-4D00D2EE2FF9

Figs 74 , 76

##### Type material.

SMF 348924, holotype, from loc. typ. (locus typicus), leg. W. De Mattia & J. Macor, May 2016.

##### Locus typicus.

Porto Santo, E of Vila Baleira, S slope of the hill above Vale do Touro, 50 m W of the oil tanks, excavated Quaternary mixed gravel, 33°03'47"N/16°19'26"W, 24 m.

##### Diagnosis.

*Hystricella* species with two keels on the body whorl, the upper less developed than the lower one; growth lines rather coarse, giving the impression of an irregular ribbing; tubercles on the base of the shell rather scattered and not as prominent as in *H. bicarinata*.

##### Description of the shell of the holotype.

Shell very small for the genus, with 5⅓ regularly increasing whorls, the protoconch with 1.6 whorls. The form of the shell is rounded conical, the convex teleoconch whorls exhibit two flat keels that become more and more expressed; the upper keel is much less developed than the lower one; keels on the body whorl located in its upper half. The last whorl measures 69%, the penultimate whorl 15% of the total shell height. The lower half of the body whorl is, beneath the distinctly angled periphery, in frontal view nearly straight, only very slightly convex. The suture between the whorls is simple, not sunken. The aperture, which is inclined to the vertical axis of the shell in an angle of 50° and is descending in the last 5% of the last whorl in an angle of 36° to the horizontal axis, has an oblique-ovate form, its width is 43% of the shell width, its height 34% of the shell height. It has a slightly reflected lip, which is completely detached from the body whorl. The eccentric umbilicus, which is approximately 11% as wide as the shell, is shaped in the upper whorls like a pinhole. The protoconch is smooth. The teleoconch exhibits a number of oblique radial ribs, 16 in the penultimate quadrant of the body whorl and is additionally covered by numerous rough tubercles. The number of tubercles in the standard-quadrate of the base is 43. There are no traces of colouration (Fig. 74).

##### Measurements.

D 4.1; H 4.2 mm; FW 2.7 mm; PA 49°; DU 0.3 mm; NT 12; NW 4.8 (*n* = 1). Ratio D/H 0.9; ratio FW/H 0.6.

##### Distribution.

**Figure 76.**
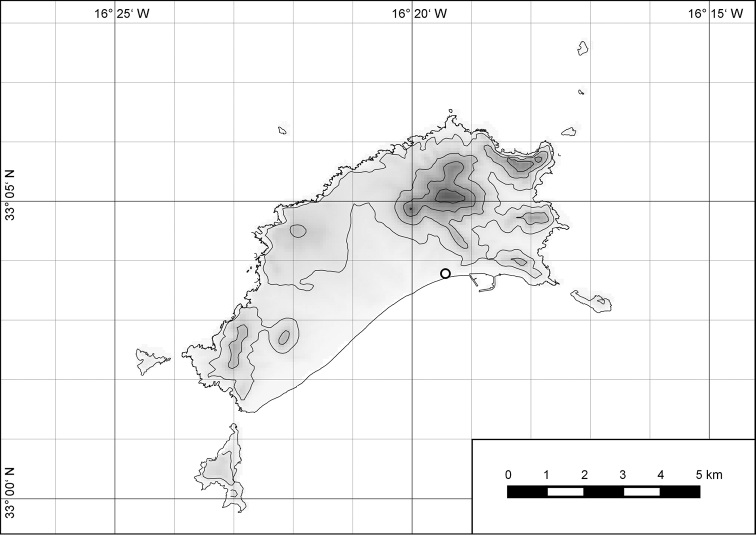
Distribution of *Hystricella microcarinata* sp. n.

The species is only known from the type locality where it appears to be extremely rare (Fig. 76). Its close resemblance to other (sub-) fossil *Hystricella* taxa may have led to misidentifications during previous collecting and sorting, thus the distribution area of the species may possibly become larger after further investigations.

##### Etymology.

The name is a combination of the Greek name for small (μικρός = mikrós) and the Latin name for keeled (carinatus) and alludes to the small size of the shell with a keel at its periphery.

##### Comparison and comments.

Being a (sub-) fossil species, only the shell features can be taken into account for comparisons. The globose shape is similar to that of some forms of *H. echinulata*; nevertheless the overall dimensions and surface sculpture of the shell clearly distinguish *H. microcarinata* sp. n. Because of its small size, the new species can also be confused with small specimens of *H. aucta* Wollaston, 1878 which, however, possesses two well-developed keels, has a wider umbilicus, a different shape and differently positioned angles of the aperture, or with *Wollastonia beckmanni* gen. et sp. n., which has a much flatter spire, has no keels on the whorls, a much wider umbilicus and also a different shape and differently positioned angles of the aperture.

##### Taxonomic remarks.

The generic affiliation of this species is based exclusively on the shape of the shell and the presence of keels on the body whorl. A similar arrangement of the tubercles and keels can be found also in some *Wollastonia* gen. n. species that will be described below.

##### Status and conservation.

Extinct before the islands’ scientific exploration in the 19^th^ century, possibly already before human settlement.

#### Hystricella echinulata

Taxon classificationAnimaliaStylommatophoraGeomitridae

(R. T. Lowe, 1831)

Figs 77–78 , 79–82 , 83–93 , 94

##### List of synonyms.

1831 *Helix echinulata* R. T. Lowe: 57, pl. 6 fig. 19.

1846 *Helix echinulata* – L. Pfeiffer: 140, pl. 91 figs 1–4.

1847 *Helix echinulata* – L. Pfeiffer in L. Pfeiffer 1847–1848: 190.

1854 *Helix echinulata* – Reeve in Reeve 1851–1854: pl. 142 figs 9–10.

1854 *Helix echinulata* – Albers: 36, pl. 9 figs 1–4.

*1855 Helix* (*Hystricella*) *echinulata* – R. T. Lowe: 186.

1867 Helix (Octephila) bicarinata var.α echinulata – Paiva: 45.

1878 Helix (Hystricella) echinulata – Wollaston: 160–161.

1950 Discula (Hystricella) echinulata – Mandahl-Barth: 31, 55.

1983 Discula (Hystricella) echinulata – Waldén: 267.

2002 *Geomitra echinulata* – Bank et al.: 124.

2008 *Hystricella echinulata* – Seddon: 79, pl. 29 fig. D (and cf. E [under *H. bicarinata*]), map 178.

2011 *Hystricella echinulata* – Seddon: e.T6727A12801253.

##### Type material.

**Figures 77–78.**
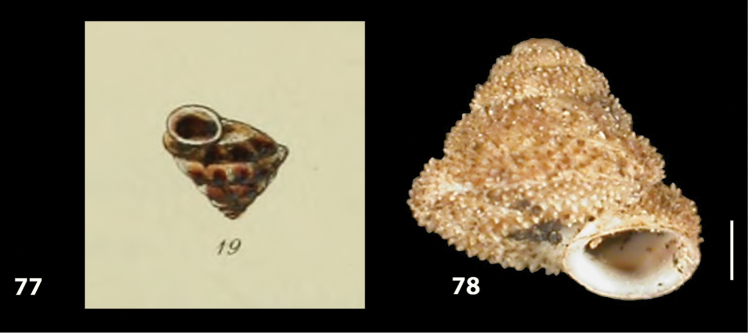
**77** original figure of *Helix echinulata* R. T. Lowe, 1831 from R. T. Lowe (1831: pl. 6 fig. 19) and **78** lectotype of *Helix echinulata* R. T. Lowe, 1831, NMH 1968.586 ex coll. R. T. Lowe. Scale bar: 1 mm.

NHM 1968.586, lectotype (herewith designated) of *Helix echinulata* R. T. Lowe, 1831 ex coll. R. T. Lowe. For the original figure of *Helix echinulata* R. T. Lowe, 1831 from R. T. Lowe (1831: pl. 6 fig. 19) and the lectotype of *H. echinulata* R. T. Lowe, 1831, W = 4.9 mm, Phot. B. Faria, DRAM, see Figs 77 and 78.

##### Further material examined.

All from Porto Santo, ANSP H 11829/1, Ribeiro do Pedregal, 33°06'10"N/16°19'25"W, 35 m, leg. K. Groh & J. Hemmen, Jul. 11 1983; ANSP H 11828/16, CKG/5, Pico do Ninho do Guincho, 33°06'07"N/16°18'51"W, 100 m, leg. K. Groh & J. Hemmen, Jul. 11 1983; CKG/1, Ribeiro do Golfeiras E Camacha, 33°05'47"N/16°19'33"W, 170 m, leg. K. & C. Groh, Oct. 25 1980; ANSP H 11831/3, CKG/1, CMN/1, SW slope of Lombo Branco, under stones, 33°05'46"N/16°18'46"W to 33°05'38"N/16°18'13"W, 150–400 m, leg. K. Groh & J. Hemmen, Jul. 8 1983; ANSP H 11830/4, Terra Chã, top, 33°05'46"N/16°18'14"W, 360 m, leg. J. Gerber, K. Groh & J. Hemmen, Aug. 14 1985; CFW 11162/5, Pico Branco, Terra Chã (350 m E of Pico Branco), 33°05'39"N/16°17'58"W, 340 m, leg. F. Walther, Mar. 31 2017; CWDM/19, CMN/5, Terra Chã, near the "Casa Forestal", under stones in pine wood. 33°05"39"N/16°20'00"W, 340 m, leg. W. De Mattia & J. Macor, May 2014; CWDM/6, Terra Chã-Pico Branco ridge, under stones, 33°05'38"N/16°18'01"W, 335 m, leg. W. De Mattia & J. Macor, May 2015; ANSP H 11833/3, summit of Pico Branco, 33°05'38"N/16°18'14"W, 450 m, leg. K. Groh & J. Hemmen, Jul. 8 1983; ANSP H 11836/15 [sub *H. leacockiana*], Pico Branco, 33°05'38"N/16°18'14"W, above 350 m, leg. J. Gerber, K. Groh & J. Hemmen, Aug. 14 1985; CKG/16, Terra Chã, top, 33°05'36"N/16°18'14"W, 360 m, leg. J. Gerber, K. Groh & J. Hemmen, Aug. 14 1985; CKG/2, Terra Chã, 33°05'31"N/16°18'16"W to 33°05'38"N/16°18'13"W, 320–400 m, leg. K. Groh & J. Hemmen, Jul. 8 1983; CWDM/14, path to Pico Branco, eastern steep part of path to Pico Branco, under stones, 33°05'29"N/16°18'14"W, 320 m, leg. W. De Mattia & J. Macor, May 2014; CFW 11148/<10, SW slope of Pico Branco ca. 250 m SW of the top, 33°05'29"N/16°18'13"W, 310 m, leg. F. Walther & E. M. Gryl, Mar. 31 2017; ANSP H 11826/7, approx. 1 km N Serra de Dentro, 33°05'24"N/16°18'27"W, 165 m, leg. J. & C. Hemmen, Jan. 6 1981; ZMH 24292/1, Madeira Archipelago, without exact locality data, ex coll. Altonaer Museum, ex coll. O. Semper, ex coll. Dohrn; ZMH 24291/1, Porto Santo, without exact locality data, ex coll. Museum Klagenfurt.

##### Locus typicus.

Hab. in monte “Pico Branco” dicto Insulae Portus S^ti^.

##### Original description.

From Lowe 1831: H. testa parvula, conoidea, sub-pyramidata, depressiuscula, supra planulata, perforata, carinata, tota scaberrima, fusca, suprà fasciata: spira pyramidata elevata; sutura distincta, impressa; anfractibus convexis; ultimi carina acuta, distincta, supra marginata sc. sulco expressa vel exarata; omnibus granulis distinctissimis, conferitis, asperrimis scobinatis et quasi echinulatis; umbilicus parvo, sub-spirali, aperto; apertura rotundata; peristomate continuo, circinato, disjuncto, reflexo. Axis 2 lin. Diam. 2½. Anfr. 6.

##### Diagnostic features of the shell.

**Figures 79–82.**
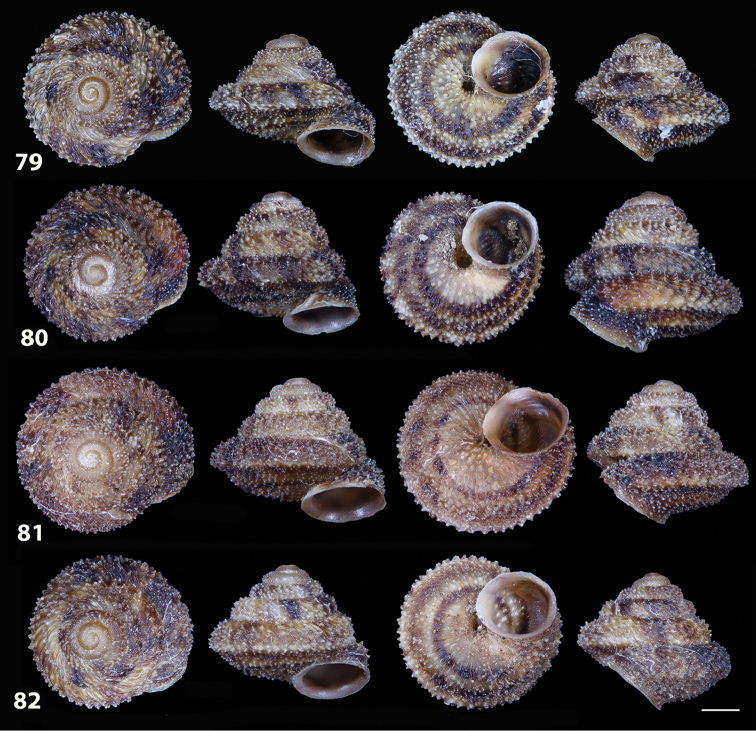
Shells of *Hystricella echinulata*. **79–80** Terra Chã **81** S slope of Pico Banco **82** steep path from Pico Branco to Terra Chã. Scale bar 1 mm.

Shell as in the genus description. The main diagnostic feature is the presence of only one keel along the body whorl. The overall shape of the shell of *H. echinulata* is always conical but not scalariform as in *H. bicarinata*. The whorls are rounded and lack the typical “shoulder”. The sutures are deep, but less deeply marked than in *H. bicarinata* (see Figs 79–82).

##### Measurements.

D 5.2 ± 0.3 mm (range 4.6–5.8 mm); H 4.8 ± 0.5 mm (range 4.3–5.0 mm); FW 2.5 ± 0.2 mm; PA 42.6 ± 8.3; DU 0.5 ± 0.08 mm; NT 23 ± 9; NW 5.6 ± 0.1 (*n* = 40). Ratio D/H 1.1; ratio FW/H 0.5.

##### Body.

Body as in the genus description. The colour of the head and the neck is somewhat darker than in *H. bicarinata*.

##### Genital anatomy.

As in the genus description. Main diagnostic features are as follows. The free oviduct is usually very short and four times shorter than the vagina. The penial flagellum is usually as long as the epiphallus. It has a remarkably cylindrical shape and a blunt apex. Its internal walls are completely smooth, without any papillae. The genital atrium is always elongated. See Figs 83–93.

**Figures 83–93.**
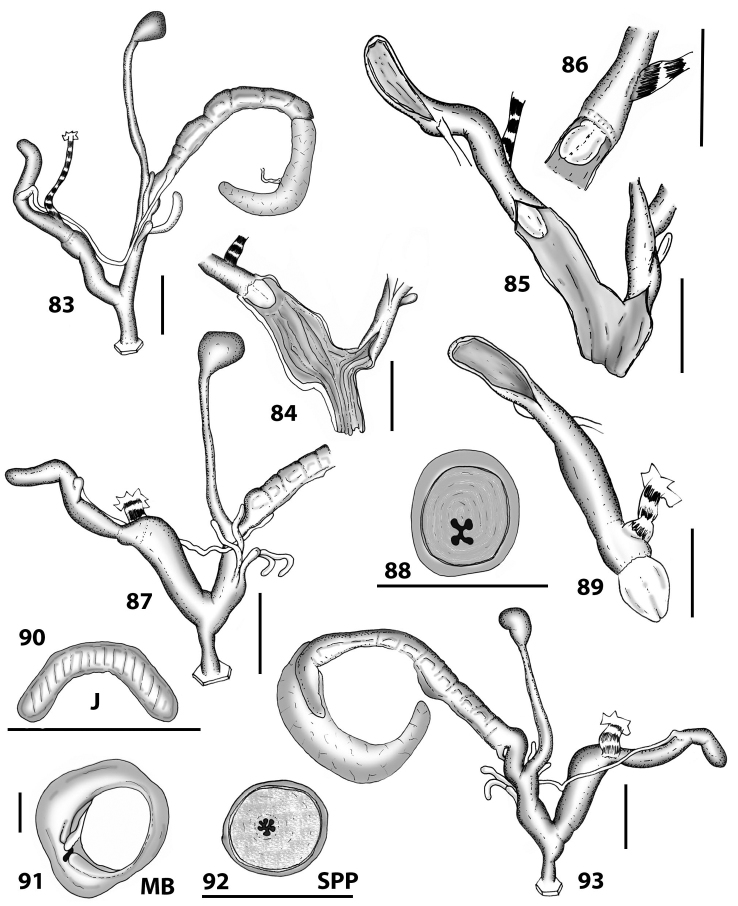
Anatomy and genitalia of *Hystricella echinulata*. Terra Chã: **83** whole genitalia excluding gonads **84** ornamentation of the inner walls of the distal penis, the distal vagina and the genital atrium **85** ornamentation of the inner walls of the flagellum, the penial complex, the vagina and the genital atrium **86** penial papilla **87** whole genitalia excluding part of OSD, AG and gonads **89** penial papilla and ornamentation of the inner walls of the flagellum **91** mantle border **92** section of penial papilla. Faja Pequena: 88 section of vagina **90** jaw **93** whole genitalia excluding gonads. Scale bars 1 mm.

##### Ecology.

*Hystricella echinulata* is recorded from low (20 m a.s.l.) to relatively high altitudes (450 m a.s.l.). Except for the slightly higher altitudinal range, this species shares the same ecology as *H. bicarinata*; it is commonly found under volcanic rocks scattered on grassland in open fields that are usually sloping. It has also been found in cracks and crevices or among scree on steep slopes.

##### Distribution.

**Figure 94.**
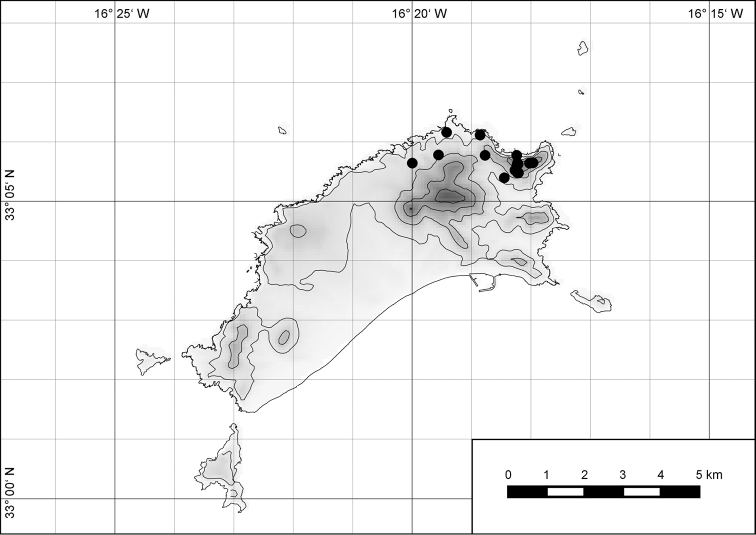
Distribution of *Hystricella echinulata*.

*Hystricella echinulata* is endemic to the island of Porto Santo (Madeiran Archipelago, Portugal). The species is restricted to the northeastern, mountainous part of the island and present only in the Pico Branco-Terra Chã massif area. It is commonly found along Faja Pequena and the ridge and the south-exposed slope eastwards of Terra Chã (Fig. 94). No populations are known from the offshore islets.

##### Comparison and comments.

See under *H. bicarinata* and/or the section on putative hybrids of *H. bicarinata* and *H. echinulata* above.

##### Status and conservation.

According to Seddon (2011b) the species is Least Concern (LC), but in our opinion, the species should be considered as Endangered (EN B1a, b(i, ii, iv), 2a, b(i, ii, iv)) because its area of occupancy and extent of occurrence is less than 8 km^2^ and because it is mostly restricted to the highest elevations in the north-eastern part of Porto Santo and only occurs at scattered and isolated places with suitably humid microclimates. Mayor threats to the species include an observed decline of suitable habitats, extent of occurrence and area of occupancy over the past 30 years, i.e. as the result of longer drought periods which are possibly associated with effects of climate change, an increase of fire incidents, grazing by goats and to a lesser extent also hiking as tourism is increasing on Porto Santo. As most of the known localities are concentrated in the Pico Branco-Terra Chã area in the north-easternmost part of Porto Santo (Fig. 94), we consider that the species is not present at more than five locations.

#### † Hystricella echinoderma

Taxon classificationAnimaliaStylommatophoraGeomitridae

(Wollaston, 1878)

Figs 95 , 96

##### List of synonyms.

1878 Helix (Hystricella) echinoderma Wollaston: 159–160.

1894 *Geomitra echinoderma* – Pilsbry in Pilsbry 1893–1895: 242.

1931 Geomitra (Actinella) echinoderma – Nobre: 88.

1950 Discula (Hystricella) echinoderma – Mandahl-Barth 1950: 31, 55.

1983 Discula (Hystricella) echinoderma – Waldén: 267.

2002 *Geomitra echinoderma* – Bank et al.: 124.

2008 *Hystricella echinoderma* – Seddon: 80.

##### Type material.

NHM 1875.02.02-52, lectotype (herewith designated) from loc. typ.; NHM 1875.02.02-53 to -54, 2 paralectotypes, from loc. typ.

##### Locus typicus.

Portum Sanctum, semifossilis; recens haud observata.

##### Original description.

From Wollaston 1878: T. trochiformis, subtus subplanulata perforata, undiquegranulis magnis obtusis sat dense obsita; spira elevata; anfractibus convexis, subgibbosis, ultimo subtectiformi acute carinato (carina simplici, solum antice gradatim obsolete subduplici); umbilico punctiformi, aperto; apertura subovali-rotundata, labris continuis conjunctis, peristomate simplici, expanso, subrecurvo, tenui, relevato. – Long. axis 2 ½ lin.; diam. 3 ½.

##### Description of the lectotype.

**Figure 95.**
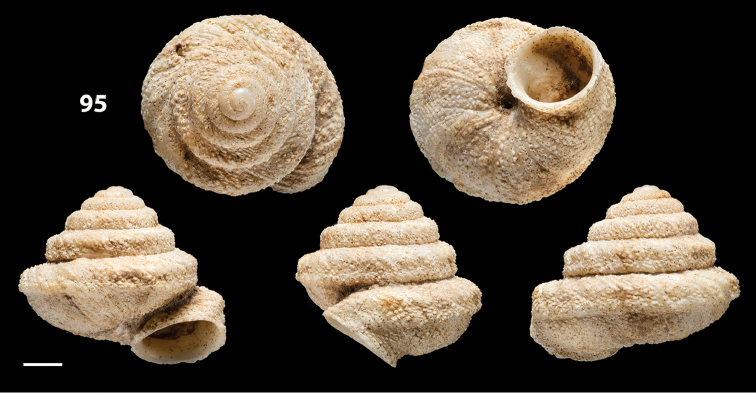
Lectotype of *Hystricella echinoderma*, NHM 1875.02.02-52. Scale bar 1 mm.

Shell large for the genus, with 6¾ regularly increasing whorls, the protoconch with 1.9 whorls. The form of the shell is high conical, the convex to quadrangular teleoconch whorls showing two more and more expressed angulations which form in the last whorl two rounded keels, the upper less developed than the lower one. The last whorl measures 59%, the penultimate whorl 14% of the total shell height. The lower 70% of the body whorl are situated below the peripheral keel which is slightly constricted by a concavity below the periphery in frontal view. The base is convex, slightly straightening towards the umbilicus. The two keels of the body whorl are located in the upper ⅛ and ⅓ of the total height of the body whorl. The suture between the whorls is simple but deeply sunken. The aperture, which is inclined to the vertical axis of the shell in an angle of 48° and descending in the last 5% of the last whorl in an angle of 38° to the horizontal axis, has an oblique-ovate form, its width amounts to 43% of the total shell width, its height to 34% of the total shell height. It has a slightly reflected lip, which is completely detached from the body whorl. The centered umbilicus, which measures 10% of the shell’s total width, is in the last whorls circular, but completely closed deeper inside. The protoconch is smooth, the teleoconch shows a number of oblique radial ribs, 15 in the penultimate quadrant of the body whorl, and is additionally covered by numerous irregularly arranged, elongate, rough tubercles. The number of tubercles in the standard-quadrate of the base is 81. There are no traces of colouration. See Fig. 95.

##### Measurements.

D 7.0 mm; H 5.9 mm; FW 3.5 mm; PA 42.2°; DU 0.5 mm; NT ≈ 35; NW 6.6 (*n* = 1). Ratio D/H 1.2; ratio FW/H 0.5.

##### Distribution.

**Figure 96.**
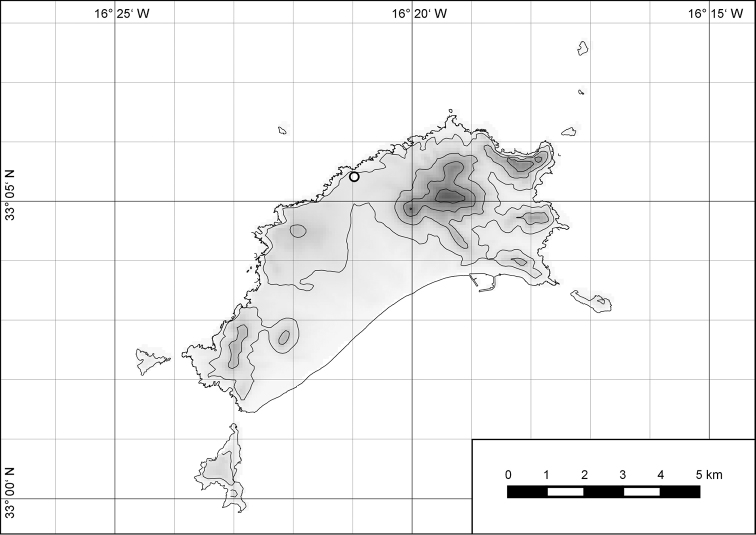
Distribution of *Hystricella echinoderma*.

*Hystricella echinoderma* is only known from Fonte da Areia in the northern part of Porto Santo (Fig. 96).

##### Comparison and comments.

*Hystricella echinoderma* can be confused on first glance with comparatively large-sized species of the genus *Wollastonia* like *W. vermetiformis*, *W. ripkeni* sp. n., and *W. falknerorum* sp. n.; from these it is distinguishable by the lack of one or two sharply pointed keels, respectively, a much rougher sculpture, a non-eccentric umbilicus, and the stepped, high conical form. From the similarly sized *W. subcarinulata* and *W. inexpectata* sp. n. it is separated by a wider or non-eccentric umbilicus, a relatively higher shell, the quadrangular instead of convex rounded whorls, a coarser granulation and the oblique-ovate instead of straight horizontally elliptical aperture. From the similar shaped *H. echinulata* and *H. microcarinata* sp. n. it is easily distinguishable by the much larger size.

##### Taxonomic remarks.

Wollaston (1878) compares *H. echinoderma* with the rather similar *H. echinulata* and calls it a monstrous sister species of that species, citing as examples of pairs of nominal species with a distinct size difference *Helix vermetiformis* and *H. bicarinata*, *H. Lowei* and *H. portosanctana* as well as *H. bowdichiana* and *H. punctulata*.

##### Status and conservation.

Extinct before the islands’ scientific exploration in the 19^th^ century, possibly already before human settlement.

#### Wollastonia gen. n.

Taxon classificationAnimaliaStylommatophoraGeomitridae

http://zoobank.org/46BC6BCC-D9E1-4753-B9A2-062537F0B077

##### Remarks.

The synonymy is the same as for the genus *Hystricella* (see above).

##### Type species.

*Helix* [*Helicella*] *turricula* R. T. Lowe, 1831 – herewith by original designation.

##### Included taxa.

*Wollastonia turricula* (R. T. Lowe, 1831), comb. n., *W. vermetiformis* (R. T. Lowe, 1855), comb. n., *W. ripkeni* De Mattia & Groh, sp. n., *W. falknerorum* Groh, Neiber & De Mattia, sp. n., *W. leacockiana* (Wollaston, 1878), comb. n., *W. beckmanni* De Mattia & Groh, sp. n., *W. jessicae jessicae* De Mattia, Neiber & Groh, sp. n., *W. jessicae monticola* De Mattia, Neiber & Groh, ssp. n., *W. klausgrohi* De Mattia & Neiber, sp. n., *W. oxytropis* (R. T. Lowe, 1831), comb. n., *W. subcarinulata* (Wollaston, 1878), comb. n., *W. inexpectata* De Mattia & Groh, sp. n.

##### Description of the genus.

**Shell.** The shell is dextral and hairless. Its shape can be very variable, from conical, elongated and scalariform with deep sutures to rather flattened, with very shallow sutures. The protoconch is from whitish to completely dark brown with 1.5 to 2.5 whorls. It is almost smooth along the first whorl and shows fine radial striae and extremely small, scattered tubercles along its remaining portion. The teleoconch has from 4.3 to 7.0 rapidly increasing whorls. It is usually dark brown with brick-red and/or dark violet shades in colour. The dark areas of the shells are mottled with more or less light brown to whitish areas, usually placed longitudinally and slightly slanting. In some species, the lighter areas tend to be more evident along the keel. No band pattern is visible along the upper whorls. On the lower part of the last whorl two dark, more or less broad bands may be present. In some specimens, the two bands merge together forming a single wide, dark band. Sometimes the bands are interrupted by yellowish to whitish sections. The area around the umbilicus is usually the lightest in colour. Some species are usually covered entirely with soil or sand grains which probably serve as a camouflage.

The spire is very variable in height, ranging from flattened, almost discoidal to slender and remarkably conical in shape. The number and the extent of the keels are variable from species to species. Some species have one or two distinct keels starting already from the second whorl of the teleoconch, while others have a single, more or less strong keel only along the last whorl. Some species have a lower, distinct principal keel somewhat protruding the body whorl, with an upper secondary and much less evident keel. The whorls can be either convex or flat. In some species, the whorls form a “shoulder”, giving the whorls and angular contour. The sutures are usually deep, even if in some species they may also be rather shallow.

The external surface has from very fine to strong, clearly visible, irregularly spaced, growth lines. Irregularly disposed tubercles are found all over the teleoconch. The dimensions and the arrangement of these tubercles varies considerably from species to species, from large and scattered tubercles to a very dense pattern of small papilla-like tubercles all over the shell. In the species with larger tubercles, these are somewhat denser along the keels of the penultimate and last whorls, giving to the keel(s) the appearance of (a) rough chord(s). The last whorl is usually large, descending near the aperture with a contribution ranging from 40% to 60% of the total shell height. The umbilicus is open but very narrow, either concentric or eccentric, and measures approximately 10% of the maximum shell diameter. The aperture is elliptical with a faint thickening along the columellar portion of the aperture. Sometimes this thickening can also extend as far the parietal side of the aperture. The peristome is continuous, slightly to evidently reflected, with the columellar margin somewhat thicker and more reflected.

The shells of the species belonging to *Wollastonia* gen. n. show a remarkable plasticity, with regard to dimensions, shape and ornamentation. This variability undoubtedly exceeds that of the genus *Hystricella*. Thus, for each taxon, the shell’s features will be described in detail.

**Body.** The head and neck are usually dark grey to grey. The sides and the posterior upper section of the foot are whitish. In some species, the pigmented ommatophoral retractor muscles are visible through the skin of the back of the cephalic area. The foot is white and the sole is longitudinally divided into three areas. The central area is smooth, whereas the two lateral areas are equipped with bands of muscles roughly arranged in a chevron pattern. The mantle border is grey to dark grey with five more or less developed lobes. The ratio of lateral to the dorsal lobes varies from specimen to specimen, also in the same population. In some specimens one of these lobes (either lateral or dorsal) may be totally missing. The walls of the pallial cavity are colourless, without any stripes or spots. A strong pulmonary vein is visible. The jaw is odontognathous and very variable in shape, from almost straight to markedly arched. There are many transversal, smooth ridges, ranging from eight to 25 in number. The right ommatophoral retractor is independent from both penis and vagina.

**Genitalia.** The general arrangement of the genitalia is semi-diaulic monotrematic. A convoluted to almost straight hermaphroditic duct arises from a multi-lobated gonad. The albumen gland is long and thin and connected to a variably long sperm-oviduct consisting of a prostatic and a uterine portion. The prostatic part extends into a thin vas deferens, roughly as long as the sperm-oviduct, and terminating in the penial complex. The distal portion of the uterine part extends into the free oviduct and turns into a vagina along its course at the level of the duct of the bursa copulatrix. The free oviduct can be as long as the vagina or also three to four times longer. The duct of the bursa copulatrix is usually wide, approximately as long as the penis and uniform in diameter. It extends into a variable, oval to roundish bursa copulatrix. In some species, the transition area between the duct and the bursa itself is not very distinctly delimited; the duct more or less abruptly widens and transforms into the bursa. The spermatophore is unknown. One tuft of digitiform glands arises from the proximal part of the vagina. The glands have usually two or three, approximately equally long and very rarely bifurcated branches. A short and thin vaginal appendix arises from the vagina’s wall, immediately distal of the glandular tuft. Very smooth, rather wide, and little elevated, irregularly spaced pleats run longitudinally along the inner surface of the vagina, reaching into the genital atrium as far as the genital orifice. The atrium can be short and wide or long and thin. Its internal walls can either be smooth or with large and soft pleats running longitudinally as far the genital orifice. The penial complex consists of a flagellum, an epiphallus (which extends from the insertion of the vas deferens to the penial retractor muscle) and a penis that inserts into the atrium. The penial flagellum is short, remarkably cylindrical and with a blunt apex. It is usually as long as the epiphallus. In one species, *W. oxytropis*, the flagellum is very short (¼ of the epiphallus) but remarkably pointed. The internal walls of the flagellum can be either completely smooth or, in one species, can have a digitiform, pointed papilla originating from the proximal end of the flagellum and orientated toward the penial papilla. The epiphallus is usually short to moderately long. Its internal walls have a variable number of longitudinal pleats that can be more or less developed and elevated. The retractor muscle is large, strong and is of a variable length. The penis lacks a muscular or glandular sheath. It is thick-walled and approximately four times longer than the flagellum. It is usually cylindrical to sometimes slightly swollen in its distal part. Sometimes, a thin sheath consisting of connective tissue envelops the distal penis, causing a partial and longitudinal minor compression. The inner walls of the penis are smooth or with irregular and spaced pleats, which run longitudinally and reach the genital atrium. The section where the penial papilla is located is usually detectable from the outside by virtue of a fine circular swelling corresponding to the origin of the papilla. The penial papilla is usually small, reaching maximally ⅛ of the total penial length and is conical to subcylindrical in shape. It has smooth external walls with the opening emerging apically. The penial papilla channel is thin and narrow. The inner lumen of the penial papilla is occupied by a spongy and sturdy tissue, which directly connects with the walls of the epiphallus. The longitudinal section of the penial papilla shows that its walls are the continuation of the penial walls that abruptly bend inwards.

**Jaw and radula.** As for the genus *Hystricella*, no notable variability was found among the species of the genus *Wollastonia* gen. n. and the two genera share almost an identical jaw-radular apparatus. The jaw is odontognathous, arched and with rough wrinkles (irregular ribs). The radula ribbon is typical helicoid, it is elongated but not very slender. A central tooth is present, tricuspid, the main cusp (endocone) is rhomboid, pointed; the ectocones are much smaller than the endocone, they are triangular, pointed. There are 19–20 laterals and marginals, which do not distinctly differ from each other. Their shapes rather change gradually from the first laterals towards the marginals. Laterals are bicuspid, with rhomboid or triangular and pointed endocones. The ectocones are much smaller, pointed, and triangular. The endocones of the central and first laterals are approximately of the same size. Both the endocone and the ectocone of the laterals gradually become bifurcated towards the marginal teeth, but the ectocones occasionally might have three cusps as well. The cusps of the marginals are gradually decreasing in size; therefore, the outermost marginals appear serrated. The jaw is variable in shape: from almost straight to markedly arched. There are many transversal and smooth ridges, ranging from 13 to 20. For *Wollastonia* jaws and radulae, see Fig. 98.

##### Distribution.

**Figure 97.**
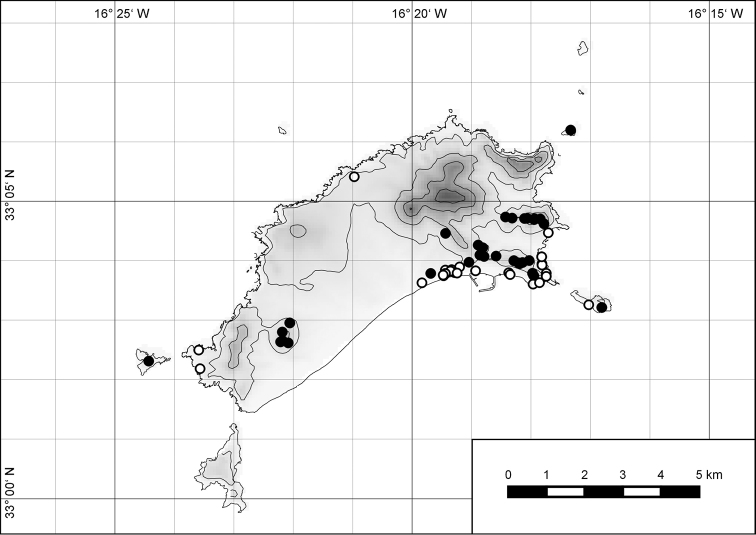
Distribution of the genus *Wollastonia* gen. n. Filled circles refer to recent and open circles to fossil records.

The genus *Wollastonia* is endemic to the island of Porto Santo (Madeiran Archipelago, Portugal) and some surrounding islets (Fig. 97). Along the eastern part of the main island, the genus is restricted to the southern mountainous part. It is patchily distributed from the hilly area east of Vila Baleira towards the east to Portela, Zimbreiro and along the highest southern peaks of Porto Santo, namely Pico do Maçarico and Pico do Baixo as far as Ponta da Galé. It is also present along the eastern coast area of Pico do Concelho. Along the western part of Porto Santo, *Wollastonia* is found on Pico de Ana Ferreira. With regard to the surrounding offshore islet, *Wollastonia* is present on Ilhéu de Cima and Ilhéu de Cenouras at the eastern and Ilhéu de Ferro at the western end of the island. Subfossil representatives of the genus are found mainly in the mud and aeolinite deposits along the southeastern (Vale do Touro, Ponta do Passo, Barbinha, Calhau da Serra de Fora) and northern (Fonte da Areia) coasts. *Wollastonia* may be older than *Hystricella* as suggested by the somewhat deeper divergences in the molecular trees (Fig. 3).

**Figure 98.**
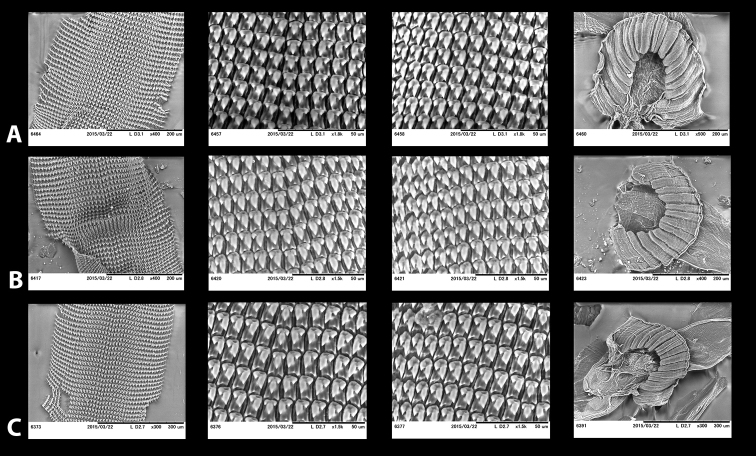
Jaws and radulae *Wollastonia* gen. n. **A** *W. leacockiana* Pico de Ana Ferreira **B** *W. oxytropis* Pico do Concelho **C** *W. jessicae jessicae* sp. n. Vale do Touro.

##### Ecology.

Representatives of the genus *Wollastonia* are commonly found under volcanic rocks scattered on grassland in open fields and more or less steep mountain slopes. Specimens aestivate on the lower surfaces of the rocks, frequently forming clusters of individuals attached to one another.

##### Etymology.

Named to honour the late British malacologist and entomologist Thomas Vernon Wollaston for his indispensable contributions to the taxonomy and nomenclature of the terrestrial snails of the mid-Atlantic islands.

##### Taxonomic remarks.

The genus *Wollastonia* gen. n. is separated here from *Hystricella* both upon molecular and morphological features. Moreover, the analysis of the distribution of its species supports this view.

The phylogenetic analyses recovered three clades, in which morphologically mostly similar species were grouped together and that were supported in at least two of the analyses. The clade including recent species hitherto assigned to *Hystricella* (except *H. bicarinata* and *H. echinulata*), supported in the BI and MP analyses (Figs 3, 5), are here regarded as representing a new genus, *Wollastonia* gen. n., because representatives of the morphologically distinct *Callina* species are interspersed between *Wollastonia* gen. n. and *Hystricella* in the strict sense receiving significant/meaningful support in the BI and MP analyses of the *cox1* data alone and the concatenated mitochondrial and nuclear data. This clade includes the recent species *W. leacockiana* comb. n., *W. turricula* comb. n., and *W. oxytropis* comb. n. as well as three additional recent taxa that will be formally described below: *W. klausgrohi* sp. n., *W. jessicae* sp. n., and *W. jessicae monticola* ssp. n. (Fig. 5). The relationships of most of the taxa within *Wollastonia* gen. n. could, however, not be well resolved (Fig. 5) on the basis of the phylogenetic analyses of the *cox1* data.

Shell size and shape are remarkably more variable in the genus *Wollastonia* as compared to *Hystricella*. Most of the *Wollastonia* gen. n. species have the shell surface covered with small and very dense tubercles that can be compared to a very fine granulation. The typical *Hystricella*’s “spiny” contour (Lat.: hystrix = spiny, deriving form Greek *ὕστριξ* (hústrix = porcupine) is replaced by a smoother one.

The genital morphology of the two genera shows no substantial differences. Nevertheless, some *Wollastonia* gen. n. species present a higher degree of complexity with regard to the inner ornamentation of the atrium and the flagellum. Structures such as more or less developed pleats, fleshy pads and/or fringes are found in *Wollastonia* gen. n. that are never present in *Hystricella*.

The distribution ranges of *Hystricella* and *Wollastonia* gen. n. are separated and not considerably overlapping. *Wollastonia* colonises the southern and southwestern parts of Porto Santo, whereas *Hystricella* is currently found exclusively in the central and northern parts.

Most of the morphological characters that clearly distinguish *Hystricella* from the other native Geomitridae of Porto Santo are also valid for *Wollastonia*. gen. n., e.g. the continuous and detached peristome and the shape of the penial flagellum that is always short and with a remarkably blunt apex (except in *W. oxytropis* stat. n.). For remarks on the differentiating features, see the respective section in the re-description of the genus *Hystricella* above.

#### Wollastonia turricula

Taxon classificationAnimaliaStylommatophoraGeomitridae

(R. T. Lowe, 1831) comb. n.

Figs 99–102 , 103–111 , 112

##### List of synonyms.

1831 *Helix turricula* R. T. Lowe: 58, pl. 6 fig. 21.

1846 *Helix turricula* – L. Pfeiffer: 141, pl. 91 figs 5–7.

1847 *Helix turricula* – L. Pfeiffer in L. Pfeiffer 1847–1848: 190.

1854 *Helix turricula* – Reeve in Reeve 1851–1854: pl. 138 fig. 867.

1854 Helix (Ochthephila) turricula – Albers: 37, pl. 9 figs 11–13.

1855 Helix (Hystricella) turricula – R. T. Lowe: 186.

1867 Helix (Octephila) turricula – Paiva: 47.

1878 Helix (Hystricella) turricula – Wollaston: 163-165.

1878 Helix (Hystricella) turricula var.ß pererosa Wollaston: 165.

1888 *Helix turricula* – Tryon in Tryon and [Pilsbry] 1888: 33, pl. 7 fig. 91.

1894 *Geomitra bicarinata* – Pilsbry in Pilsbry 1893–1895: 242.

1923 *Ochthephila turricula* – Watson: 283–293, pl. 6 figs 1–10.

1931 Geomitra (Actinella) bicarnata – Nobre: 88, fig. 38.

1950 Discula (Hystricella) turricula – Mandahl-Barth: 31, 55.

1950 Discula (Hystricella) turricula pererosa – Mandahl-Barth: 31, 55.

1977 Discula (Hystricella) turricula – Pettitt: 147–150.

1983 Discula (Hystricella) turricula – Waldén: 267.

2002 *Geomitra turricula* – Bank et al.: 124.

2008 *Hystricella turricula* – Seddon: 79, pl. 29 fig. b, map 181.

2009 Hystricella turricula f. pererosa – Groh et al.: 21 fig. 28.

2011 *Hystricella turricula* – Seddon: e.T6723A12800477.

##### Type material.

**Figures 99–102.**
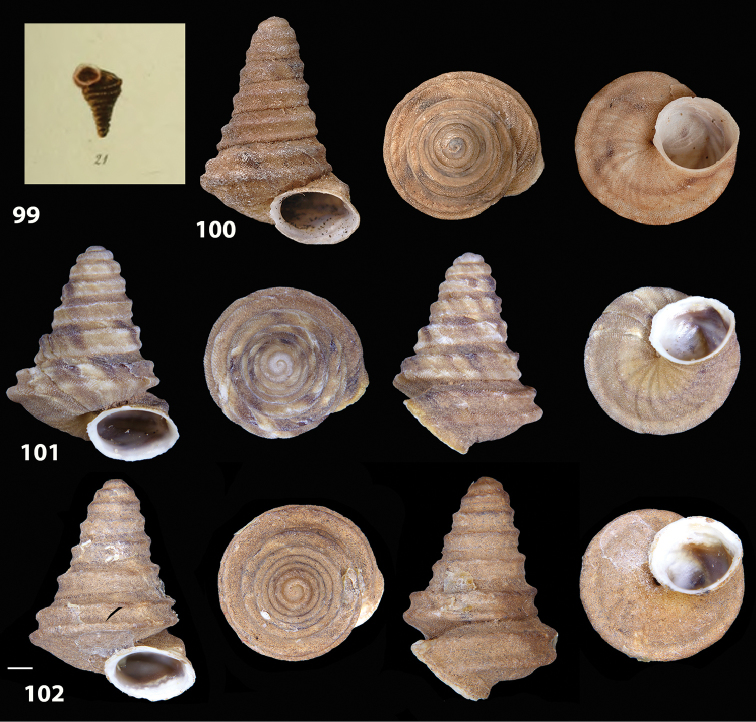
**99** *Wollastonia turricula* original figure as in R. T. Lowe, 1831 (from Lowe 1831: pl. 6 fig. 21). Shell of *Wollastonia turricula* **100** shell of the lectotype from loc. typ., ex coll. R. T. Lowe NMH 1948.7.8.35 **101, 102** shells from Ilhéu de Cima. Scale bar 1 mm.

[*turricula*], NMH 1968.578, lectotype (herewith designated), from loc. typ., ex coll. R. T. Lowe; NMH 1948.7.8.35, 1 paralectotype, from loc. typ., ex coll. R. T. Lowe. The original figure of *Helix turricula* R. T. Lowe, 1831 (from Lowe 1831: pl. 6 fig. 21) is depicted in Fig. 99, the lectotype (Phot. P. Crabb, NHM) in Fig. 100; [*pererosa*], RAM EXEMS-1720-1909-d39-74a, lectotype (herewith designated), from loc. typ., ex coll. Linter, ex coll. T. V. Wollaston; RAM EXEMS-1720-1909-d39-74b, 1 paralectotype, from loc. typ., ex coll. Linter, ex coll. T. V. Wollaston (see Figs 113–115).

##### Loci typici.

[*turricula*], Hab. in Insula quadam «Ilheo de Cima» dicta, juxta Insulam Portum S^tum^; [*pererosa*], ‘Ilheo de Cima’.

##### Further material examined.

All from Porto Santo. Fossil: CKG/10, CWDM/2, ZMH 116090/1 fragm [ex coll. E. Clauss], Quaternary slope deposits at the SW coast of the Ilhéu de Cima, 33°03'15"N/16°17'02"W, 40 m, leg. K. & C. Groh & J. & C. Hemmen, Jun. 26 1983 and leg. J. Gerber, K. Groh & J. Hemmen, Aug. 17 1985. Recent: CKG/9, Ilhéu de Cima, top, under stones, 33°03'13"N/16°16'48"W, approx. 100 m, leg. K. & C. Groh, Oct. 25 1980; CKG/7, CMN/29, ANSP H 11918/42, ZMH 120611/1 [ex coll. W Fauer], Ilhéu de Cima, top, under stones, 33°3'13"N/16°16'48"W, approx. 100 m, leg. K. & C. Groh & J. & C. Hemmen, Jun. 26 1983; ANSP H 11918/17, Ilhéu de Cima, top, under stones, 33°3'13"N/16°16'48"W, approx. 100 m, leg. J. Gerber, K. Groh & J. Hemmen, Aug. 17 1985; ZMH 24296/1, Porto Santo, without exact locality data, ex coll. Altonaer Museum, ex coll. O. Semper, ex coll. Dohrn; ZMH 24297/1, Porto Santo, without exact locality data, ex coll. Museum Klagenfurt; ZMH 24298/1, Porto Santo, without exact locality data, ex coll. Altonaer Museum.

##### Original descriptions.

[*turricula*]: From Lowe 1831: H. testa turrita, pyramidata, sub-cylindrica, bicarinata, perforata, tota minute et confertissime granulata, fusca, fere unicolore, vel supra obsolete fasciata: spira valde elevata, obtusissima; sutura distincta; anfractibus bicarinatis, carinis æqualibus, prominentibus, distinctis, sulco divisis: apertura rotunda; peristomate continuo, circinato, disjuncto, tenui, reflexo. Axis 4 lin. Diam. 3. Anfr. 8–8½; [*pererosa*]: From Wollaston 1878: ... a keel, very largely developed in the ‘α. *pererosa*,’ in the centre of each, causing the basal volution to be strongly bicarinated ….. var. ß. *pererosa*. – Plerumque obscurior, spira breviore, anfractibus in medio multo grossius carinatis (carina altissima), ultimo sensim latiore necnon antice obsolete subtortuoso, fere quasi superimposito, apertura submajore.

##### Redescription of the shell.

The shell is dextral and hairless. It is remarkably elongated and scalariform with deep sutures. The protoconch is from whitish to completely dark brown with 1.5 to 2 whorls. It is finely granulated along the first whorl and shows also fine radial striae and extremely small, scattered tubercles along its remaining portion. The teleoconch has from 6.9 to 7.2 rapidly increasing whorls. It is usually reddish brown, with brick red and/or dark violet shades in colour. The dark areas of the shell are mottled with more or less light brown to whitish areas, usually placed longitudinally and slightly slanting. In some specimens, the lighter areas tend to be more evident along the keels. No band pattern is visible along the upper whorls. On the lower part of the last whorl there is sometimes one dark, indistinct band that is usually very narrow. The area around the umbilicus is usually the lightest in colour. The shell is usually covered by fine debris serving as a camouflage.

The spire is very high and remarkably conical in shape. Two evident keels start already from the second whorl of the teleoconch. The keels gradually strengthen, reaching their maximum extent along the body whorl. The lower and the upper keel are more or less equally developed, the lower only sometimes slightly more pronounced. The lower whorls (especially the fourth and fifth) form a “shoulder” giving the whorls an angular contour. The sutures are deep and well marked.

The external surface does usually not show fine growth lines, except for some strong, more or less regularly spaced ribs along the top and bottom of the body whorl. Irregularly arranged, fine tubercles are present all over the teleoconch, also along the edges of the keels the tubercles remain well-separated not forming a peripheral chord.

The last whorl is not distinctly wider than the penultimate whorl, nevertheless abruptly descending near the aperture. The umbilicus is very narrow, almost closed, and somewhat eccentric. The aperture is elliptical with a faint thickening along the columellar portion of the stoma. Sometimes this thickening can also extend as far as the parietal side of the aperture. The peristome is continuous and detached from the body whorl, reflected, with the columellar margin somewhat thicker and more distinctly reflected (Figs 101–102).

##### Measurements.

D 6.6 ± 0.4 mm (range 6.1–7.0 mm); H 8.8 ± 0.2 mm (range 6.1–9.0 mm); FW 4.3 ± 0.1 mm; PA angle 32.1 ± 2.1°; NT > 100; NW 7.0 ± 0.1 (*n* = 8). Ratio D/H 0.8; ratio FW/H 0.5.

##### Body.

The head and the neck are usually dark grey to grey. The sides and the posterior upper section of the foot are whitish. The foot is white and the sole is longitudinally divided into three areas. The central area is smooth, whereas the two lateral areas are equipped with bands of muscles that are roughly arranged in a chevron pattern. The mantle border is dark grey with five more or less developed lobes. The walls of the pallial cavity are colourless, without any stripes or spots. A strong pulmonary vein is visible. The jaw is odonthognatous and its shape is markedly arched. There are up to 25 smooth transverse ridges. The right ommatophoral retractor is independent from both penis and vagina.

##### Genital anatomy.

The albumen gland is short and as long as the sperm-oviduct. The prostatic part of the sperm-oviduct extends into a vas deferens that is approximately as long as the sperm-oviduct, which is inserting into the penial complex. The free oviduct is two times longer than the vagina. The duct of the bursa copulatrix is rather wide, slightly shorter than the penis, and uniform in diameter. It terminates in a roundish bursa copulatrix. The transition area between the duct and the bursa is very sharply delimited, abruptly widening and turning into the bursa. The spermatophore is unknown. One tuft of digitiform glands arises from the proximal part of the vagina. There are usually two rather wide glands that are approximately equally long and very rarely branched. A short and thin vaginal appendix arises from the wall of the vagina, just distal of the glandular tuft.

Very smooth, rather widely and irregularly spaced and little elevated pleats run longitudinally along the inner surface of the vagina, reaching into the genital atrium as far as the genital orifice. The atrium is rather wide. Its internal walls are equipped with large and soft pleats, running longitudinally towards the genital orifice. The penial flagellum is short, remarkably cylindrical and with a blunt apex. It is usually as long as the epiphallus. Its internal walls are equipped with a digitiform papilla that originates at the proximal end of the flagellum and which is orientated towards the penial papilla. The epiphallus is approximately half as long as the penis. Its internal walls are usually equipped with two to three longitudinal pleats. These pleats are arranged more or less “metameric”.

**Figures 103–111.**
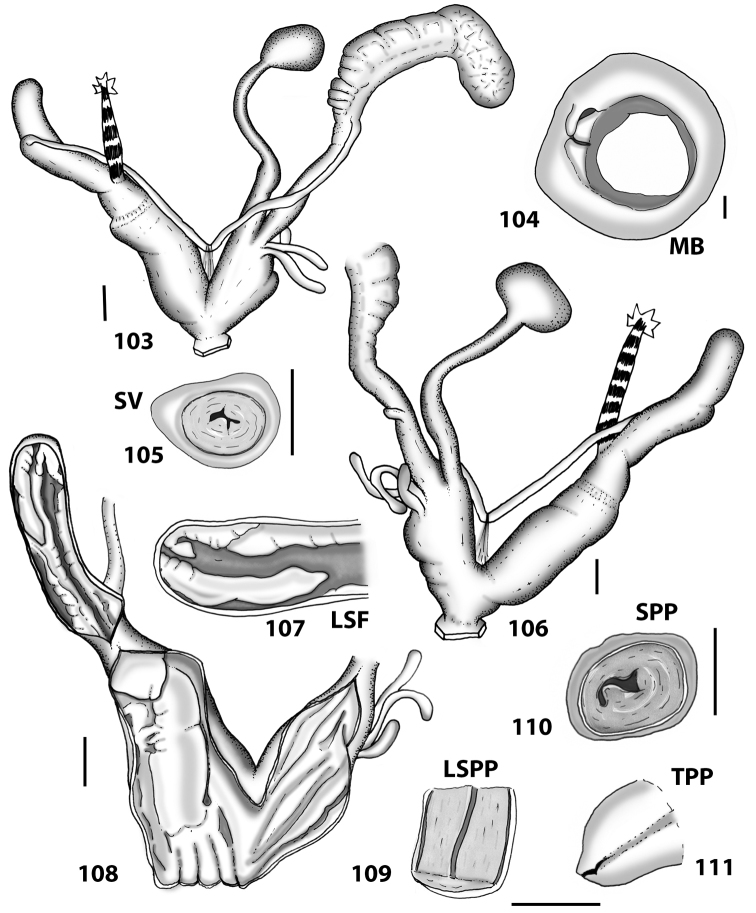
Anatomy and genitalia of *Wollastonia turricula*, Ilheu de Cima. 103 whole genitalia excluding gonads **104** mantle border **105** section of vagina **106** whole genitalia excluding part of OSD, AG and gonads **107** ornamentation of the inner walls of the flagellum **108** ornamentation of the inner walls of the flagellum, the penial complex, the vagina and the genital atrium **109** longitudinal section of penial papilla **110** section of penial papilla **111** tip of penial papilla. Scale bars 1 mm.

The retractor muscle is large, strong and is of variable length. The penis lacks any muscular or glandular sheath. It is thick-walled and approximately four times longer than the flagellum. It is cylindrical and slightly swollen in its proximal portion. The inner walls of the penis are equipped with a large longitudinal, fleshy, and smooth pleat, running from the penial papilla almost as far as the atrium. Some minor, smooth, longitudinal pleats are usually also present. The penial papilla is short but somewhat bulky. Its surface is smooth, with the opening emerging apically; its channel is rather narrow. The inner lumen of the penial papilla is filled with a spongy and sturdy tissue, which directly connects with the walls of the epiphallus. The longitudinal section of the penial papilla shows that its walls are the continuation of the penial walls that abruptly bend inward. See Figs 103–111.

##### Ecology.

*Wollastonia turricula* is found under volcanic rocks scattered on grassland in open fields.

##### Distribution.

**Figures 112–116.**
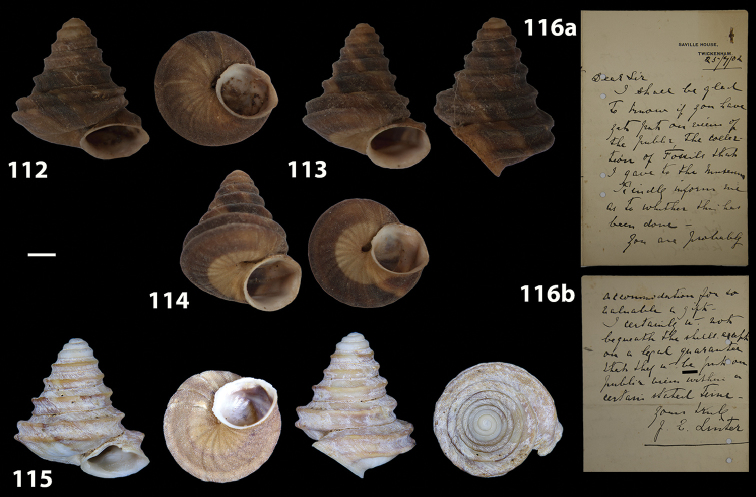
**112–114** shells of Helix turricula var. pererosa RAM EXEMS-1720-1909-d39-74 **115** Ilheu de Cima **116a, 116b** labels from RAM. Scale bar 1 mm.

Restricted to the slopes and plateau of the islet Ilhéu de Cima, off the southeastern coast of Porto Santo. The species occupies an area of less than two square kilometres (Fig. 112). The form pererosa of *W. turricula* is known to occur on the steep slopes at lower elevations of the islet Ilhéu de Cima.

##### Comparison and comments.

*Wollastonia turricula* is easily distinguishable from all the species belonging to either *Hystricella* or *Wollastonia* gen. n. by its peculiarly turreted shell and genital features. The anatomical and genital features of the species, under the name *Ochtephila turricula*, have previously been thoroughly described by Watson (1923), nevertheless Watson’s study limited its observations mostly to the external features of the genital system. Unfortunately, except for some drawings presented by Mandahl-Barth (1950), no additional information on the genital anatomy was published until now. Watson’s (1923) description, despite being thorough and accurate, sometimes gives a wrong impression of what the author observed. On p. 289 the author describes “three small finger-shaped processes” pointing out that “the shortest of the three is usually less than half the length of the others”. He considered all these three processes “homologous with the so called mucous glands found in so many of the Helicidae”. Watson considered also the vaginal appendix as a gland that is probably an apomorphic state of the stylommatophores. He also wrongly considered the “conspicuous hemispherical swelling” on the outer side of the vagina to be “doubtless a degenerate dart-sac”. It is known that the dart sac can assume such a “swollen” appearance, as for example in the genus *Cernuella*, but its inner structure shows a totally different arrangement compared to that found in *W. turricula* (see Giusti et al. 1995: 445). Watson also described the internal ornamentation of the penis as “smooth”, not detecting the main fleshy pleat and its surrounding secondary small pleats. Nevertheless, Watsons’ (1923) description as a whole is thorough and accurate, including also interesting notes about the histology of the tissues. This kind of anatomical description was very rare at the time, where conchological features were still considered as the most important taxonomical characters in land snail systematics.

##### Taxonomic remarks.

After its description, the taxon Helix turricula var. pererosa Wollaston, 1878 was mostly ignored by subsequent authors, except by Mandahl-Barth (1950) who treated it as a subspecies of *W. turricula*. Three syntypes of Helix turricula var. pererosa Wollaston (see Figs 112–114) could be traced at the Exeter Museum (coll n°EXEMS-1720-1909-d39-74). The preservation of the specimens clearly indicates that they were collected alive and not as subfossils. Following a careful morphological evaluation and a comparison with recent and subfossil specimens (Fig. 115), no substantial morphological differences could be detected with regard to the shell morphology, both concerning its micro- and macroscopic features (microsculpture, overall shape, keels, dimensions). Therefore, we treat it here as just a form of *Wollastonia turricula* and propose the name Helix turricula var. pererosa Wollaston, 1878 as a junior synonym of *Wollastonia turricula*. Pettitt (1977) reports on significant changes in the shell shape of *W. turricula* after the construction of the lighthouse on the islet Ilhéu de Cima after 1900. However, that observation may be based on material collected prior to the construction works and therefore may have contained more specimens of the form pererosa because the top of the islet was only easily accessible after the construction of the stairs to the lighthouse and therefore collecting probably was carried out nearer to the coast where the *pererosa* form predominates.

##### Status and conservation.

**Figure 117.**
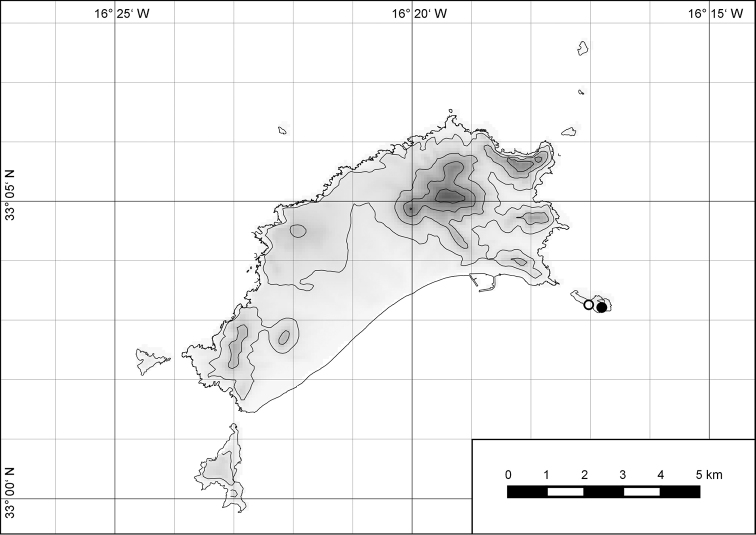
Distribution of *Wollastonia turricula*. Filled circles refer to recent and open circles to fossil records.

According to Seddon (2011e) the species is Vulnerable (VU). In our opinion, however, it should be regarded as Critically Endangered (CR B1a, b(ii, v), 2a, b(ii, v)) because the extent of occurrence and the area of occupancy of the species is less than 1 km^2^ and although Watson (1923) reported the species to “occur in considerable numbers” on Ilheu de Cima, observations made during the 1980s (KG) showed that the species is considerably less frequent than in the past, probably as the result of a decline of habitat quality as a consequence of grazing by goats or other unknown reasons (see also Seddon (2008: 80)). Moreover, living specimens were only reported from the area around the top plateau of Ilheu de Cima, i.e., at a single location (Fig. 117).

#### Wollastonia vermetiformis

Taxon classificationAnimaliaStylommatophoraGeomitridae

(R. T. Lowe, 1855) comb. n.

Figs 118–120

##### List of synonyms.

1855 Helix (Hystricella) vermetiformis R. T. Lowe: 186.

1867 Helix (Octephila) vermetiformis – Paiva: 47–48.

1867 Helix (Octephila) vermetiformis var.α minor Paiva: 48.

1878 Helix (Hystricella) vermetiformis – Wollaston: 163.

1894 *Geomitra vermetiformis* – Pilsbry in Pilsbry 1893–1895: 242.

1931 Geomitra (Actinella) bicarinata var. vermetiformis – Nobre: 87.

1950 Discula (Hystricella) vermetiformis – Mandahl-Barth: 31, 55.

1983 Discula (Hystricella) oxytropis vermetiformis – Waldén: 267.

2002 *Geomitra vermetiformis* – Bank et al.: 124.

2006 *Discula oxytropis* – Cameron et al.: 40 [partim].

2008 *Hystricella oxytropis* – Seddon: pl. 29 fig. C, map 177 [partim; in her “Corrigenda” Seddon (2009) mentions for pl. 29 fig. C in Seddon (2008) that the ”Shell of *Hystricella oxytropis* is normally approx. 8 mm wide, not 5 mm, and does not always have a double keel”].

2009 *Hystricella oxytropis* – Seddon: 80 [partim].

2009 *Hystricella vermetiformis vermetiformis* – Groh et al.: 21, fig. 29.

2017 *Hystricella vermetiformis* – Groh: e.T107396913A107396917.

##### Type material.

**Figures 118–120.**
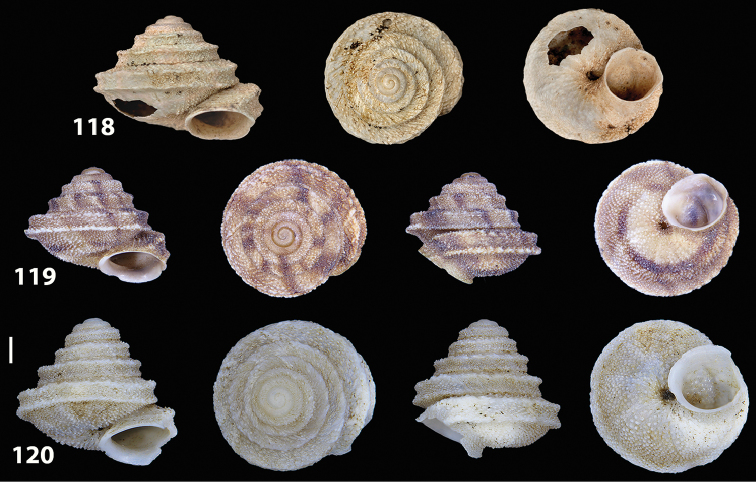
Shells of *Wollastonia vermetiformis*. **118** lectotype of *Helix vermetiformis* R. T. Lowe, 1855, NMH 1968.588 ex coll. Lowe **119** recent specimen from Pico do Baixo **120** subfossil specimen from Porto dos Frades. Scale bar 1 mm.

NHM 1968.588, lectotype (herewith designated), from loc. typ. ex coll. R. T. Lowe. The lectotype is depicted in the Fig. 118.

##### Locus typicus.

[*vermetiformis*] “Hab. fossilis in Portu S^to^” (= Porto Santo, fossil); [*minor*] rara ad Zimbral d'Aréa [= Zimbral da Areia (at the SE coast of Porto Santo, N of Barbinha-Porto dos Frades), 33°04'25"N/16°17'46"W].

##### Additional material.

All from Porto Santo. Fossil: CWDM/4, Barbinha, a little distance south along the old rough road, mud fossil deposits, 33°03'56"N/16°17'49"W, 10 m, leg. W. De Mattia & J. Macor, May 2015; CKG/3, W coast, Ribeiro de Agua, 33°02'11"N/16°23'34"W, 50 m, leg. C. Groh, Jul. 27 1983; CKG/1, approx. 800 m E Vila Baleira, approx. 60 m N of oil tanks at bridge over Vale do Touro, from Quaternary tuffite layers, 33°03'51"N/16°19'20"W, 30 m, leg. K. Groh & J. Hemmen, Jul. 27 1983; CKG/1, Ponta da Canaveira, Quaternary aeolinites, 33°02'30"N/16°23'35"W, 50 m, leg. K. Groh & J. Hemmen, Jun. 24 1983; CKG/10, between Porto dos Frades and Ferreira Grande, approx. 300 m N sand pit, 33°04'28"N/16°17'42"W, 25 m, leg. K. Groh & J. Hemmen, Jun. 24 1983; CKG/1, Barbinha, uppermost light Quaternary aeolinite layer, 33°04'04"N/16°17'49"W, 8 m, leg. K. & C. Groh & J. & C. Hemmen, Jun. 24 1983; ANSP H 11919/21, Barbinha, Quaternary aeolinites, 33°04'04"N/16°17'49"W, 8 m, leg. K. & C. Groh & J. & C. Hemmen, Jul. 7 1983; ZMH 110125/34, coastal slopes at road from harbour to Porto Santo [Vila Baleira], 33°03'48"N/16°19'17"W, c. 10–50 m, leg. E. Clauss, Sep. 22 1992; ZMH 110139/23, coastal slopes at road from harbour to Vila Baleira, 33°03'48"N/16°19'17"W, c. 10–50 m, leg. E. Clauss, Sep. 22 1992; ZMH 110104/5, coastal slopes in the SE to E coast of Porto Santo, 33°03'46"N/16°18'21"W, c. 10–50 m, leg. E. Clauss, Jun. 1 1996; ZMH 24295/1, Madeira archipelago, without exact locality data, ex coll. Altonaer Museum, ex coll. O. Semper, ex coll. Dohrn. Recent: CKG/1, W slope of Pico do Baixo, in a rock crack, 33°03'47"N/16°17'58"W, approx. 125 m, leg. K. Groh & J. Hemmen, Jun. 9 1983; CKG/2, Ilhéu das Cenouras, 33°06'12"N/16°17'20"W, 20 m, leg. K. Groh & J. Hemmen, Jul. 2 1983.

##### Original description.

[*vermetiformis*]: From Lowe 1855: T. anguste umbilicata distincte bicarinata pyramidato-conoidea solidula crassiuscula utrinque granulata; spira elevata anfractui ultimo quasi superimposita, carina inferiore suturae distinctæ superincumbente; anfr. 7–7½ planiusculis conspicue bicarinatis, carina inf. prominente sulco infra exarata, ult. antice valde deflexo; umbil. parvo; apert. ovali-rotundata circinata, labris continuis conjunctis; perist. undique soluto relevato tenui acuto. Diam. major 8½–9, min. 8¼–8¾, alt. 7–8½ mill. Anfr. 7–7½; [var. minor]: From Paiva 1867: Variet. adest α *minor*, testa minore.

##### Diagnosis.

Shell large for the genus, conical, scalariform. Whorls not rounded, vertical. Surface of the shell covered by small and relatively densely set tubercles. Second whorl and body whorl bicarinated, with upper keel weaker than the lower one. Umbilicus narrow. Last whorl descending towards the aperture. Aperture oval with continuous peristome.

##### Redescription of the shell.

Shell medium to large for the genus, with 6½ regularly increasing whorls, the protoconch with 2 whorls. The form of the shell is conical, the convex teleoconch whorls with two sharp keels. The last whorl measures 60%, the penultimate whorl 17% of the total shell height. The lower ⅔ of the body whorl are beneath the lower peripheral keel, which is set off by a relatively deep constriction above and below the keel; outline of body whorl below the lower constriction regularly convex in frontal view. The keels of the body whorl are placed in the upper twelfth and third, respectively, of its total height. The suture between the whorls is simple and slightly sunken. The aperture, which is inclined to the vertical axis of the shell in an angle of 40° and is descending in its last 5% in an angle of 31° to the horizontal axis, has an elliptic form; its width is 42% of the total shell width and its height 26% of the total shell height. It has a slightly reflected, narrow lip, which is completely detached from the body whorl. The eccentric umbilicus, which measures 11% of the shell's total width, is circular in the last whorls and completely closed in earlier whorls. The protoconch is smooth, the teleoconch shows a number of oblique radial ribs, 19 in the penultimate quadrant of the body-whorl and is additionally covered by numerous small, ovate and rough tubercles. The number of tubercles in the standard-quadrate of the base is estimated to approximately 70. There are no traces of colouration in subfossil shells. In recent specimens, the colour pattern of the shell is the same as in *H. bicarinata*. Figs 118–120.

##### Variation of shell.

The spire can be more or less elevated but always letting the shell appear remarkably conical and scalariform in shape. Subfossil and recent material does not show much variation.

##### Measurements.

D 6.3 ± 0.4 mm (range 5.6–7.0 mm); H 6.1 ± 0.6 mm (range 5.8–6.6 mm); FW 3.7 ± 0.2 mm; PA 44.0 ± 3.2°; DU 0.6 ± 0.07 mm; NT 70 ± 16; NW 6.75 ± 0.2 (*n* = 7). Ratio D/H 1.0; ratio FW/H 0.6.

##### Distribution.

**Figure 121.**
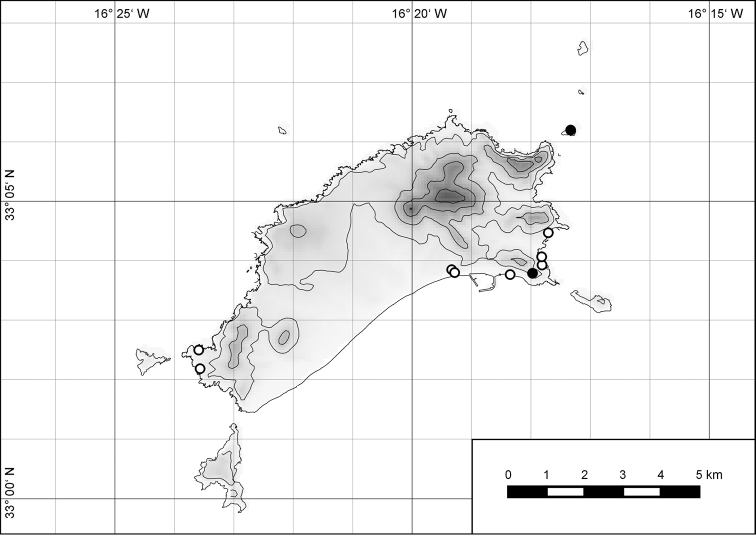
Distribution of *Wollastonia vermetiformis*. Filled circles refer to recent and open circles to fossil records.

Subfossil material is known, except for one location in the westernmost part of the island, exclusively from the southeastern coast of Porto Santo, i.e. the mud deposits and aeolinites along Barbinha, Zimbral da Areia and Porto dos Frades. Recent, living specimens are only known from Pico do Baixo and Ilhéu das Cenouras, collected during the 1980’s. Other recent material is kept at the NMWC 80.202, acc. 55.158 (ex coll. Melvill-Tomlin, ex coll. T. V. Wollaston) and NMWC 2004.045.00055 (ex coll. Coles) (see Seddon 2008: pl. 29 fig. C), but without exact locality data (just: Porto Santo). The distribution is shown in Fig. 121.

##### Ecology.

Living specimens were collected in the 1980s on slopes under stones covered with lichens and in a rock crack. More field research is necessary for a better understanding of the ecology and actual distribution of this species.

##### Comparison and comments.

*Wollastonia vermetiformis* is superficially similar to *H. echinoderma*, *W. falknerorum* sp. n. and *W. ripkeni* sp. n. From the first two it differs in the development of the two keels on the body whorl, and from the second also in the much coarser shell sculpture, the more pronounced upper keel, and the narrower aperture. From the quite similar *H. ripkeni* sp. n. it differs in the lower last whorl, the oblique ovate aperture, the coarser granulation, and finer ribbing. From similar-sized *W. subcarinulata* and *W. inexpectata* sp. n. it can be separated by the coarser granulation and the presence of two keels. The last known recent specimens were collected during the 1980s. Unfortunately, this material was not stored in ethanol. Therefore, an anatomical and molecular investigation was not possible. Recent intensive field research (on the main island Porto Santo and its outlying islets, D. Teixeira pers. comm. 2016) failed to find any living specimen of *W. vermetiformis* on the Pico do Baixo. The species may possibly have gone extinct in the 1980s on the main island.

##### Status and conservation.

Only three recent specimens of the species collected in the 1980s (KG) from two isolated localities (Porto Santo, W slope of Pico do Baixo, in a rock crack and Ilhéu das Cenouras; Fig. 121) are known. Despite intensive recent field research, the species has not been refound and is probably extinct on the main island of Porto Santo. Although recent data are missing for Ilhéu das Cenouras, we cannot exclude the existence of extant living populations on that small offshore islet. We regard the species as Critically Endangered (CR B1a, b(i, ii, iv), 2a, b(i, ii, iv)) (see also Groh 2017) because the extent of occurrence and the area of occupancy of the species is less than 1 km^2^ and because the species is arguably extinct at one of the two known locations (Fig. 121).

#### † Wollastonia ripkeni

Taxon classificationAnimaliaStylommatophoraGeomitridae

De Mattia & Groh sp. n.

http://zoobank.org/A2E91669-0659-447D-9EB3-2F02174D4E87

Figs 122–124 , 125

##### Type material.

SMF 348929, holotype, from loc. typ., leg. W. De Mattia & J. Macor, May 2015, NMWC Z.2016.013.00008, 2 PT (paratypes), NHMW 112144/1 PT, CKG, 2 PT, CWDM, 11 PT, from loc. typ., leg. W. De Mattia & J. Macor, May 2015.

##### Locus typicus.

Porto Santo, excavated mud walls behind the cart speedway E of the new harbour of Porto Santo, 33°03'48"N/16°18'22"W, 30 m.

##### Diagnosis.

Shell large for the genus, solid and conical. Whorls not rounded, not vertical but somewhat slanting. Surface of the shell covered by small, and relatively densely set tubercles. Body whorl bicarinated, with upper keel weaker than the lower one. Remaining teleoconch whorls only with the upper keel visible. Umbilicus narrow. Last whorl descending toward the aperture. Aperture oval with continuous peristome.

##### Description of the holotype.

**Figures 122–124.**
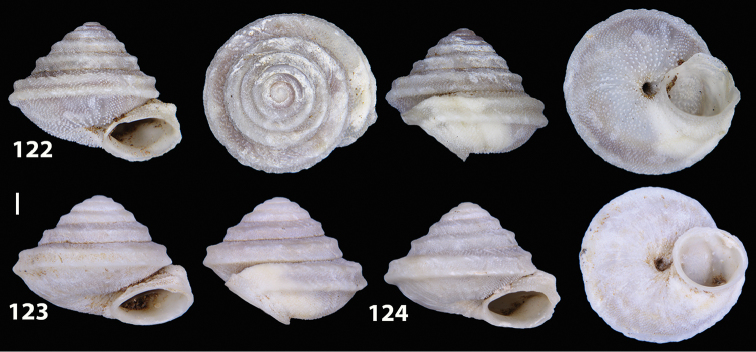
Shells of *Wollastonia ripkeni* sp. n. **122** holotype, SMF 348929 **123, 124** paratypes from the loc. typ. Scale bar 1 mm.

Shell large for the genus, with 6⅔ regularly growing whorls, the protoconch with 1.85 whorls. The form of the shell is conical, the convex teleoconch whorls showing two rounded keels, the upper one less developed than the lower one. The last whorl measures 66%, the penultimate whorl 12% of the total shell height. The lower ¾ of the body whorl are beneath the lower peripheral keel, which is set off by a constriction above and below the keel; outline of body whorl below the lower constriction regularly convex in frontal view. The two keels of the body whorl are placed in the upper ^1^∕_11_ and ¼ of its total height. The suture between the whorls is simple, not sunken. The aperture, which is inclined to the vertical axis of the shell in an angle of 50° and is descending in the last 5% of the last whorl in an angle of 35° to the horizontal axis, has an elliptic form; its width is 46%, of the total shell width, its height 31% of the total shell height. It has a slightly reflected lip, which is completely detached from the body whorl. The eccentric umbilicus, which measures 10% of the total shell width, is in the last whorls circular and completely closed in the earlier whorls. The protoconch is smooth, the teleoconch shows a number of oblique radial ribs, 13 in the penultimate quadrant of the body-whorl and is additionally covered by numerous rounded tubercles. The number of tubercles in the standard-quadrate of the base is 89. There are no traces of colouration (Figs 122–124).

##### Variation of the paratypes.

Although the shell size is slightly variable, its shape is remarkably stable in all specimens.

##### Measurements.

D 8.0 ± 0.4 mm (range 7.2–8.6 mm); H 6.2 ± 0.4 mm (range 5.4–6.8 mm); FW 4.1 ± 0.2 mm; PA 42 ± 4.1°; DU 0.9 ± 0.08 mm; NT 89 ± 23; NW 6.7 ± 0.2 (*n* = 16). Ratio D/H 1.3; ratio FW/H 0.7.

##### Distribution.

**Figure 125.**
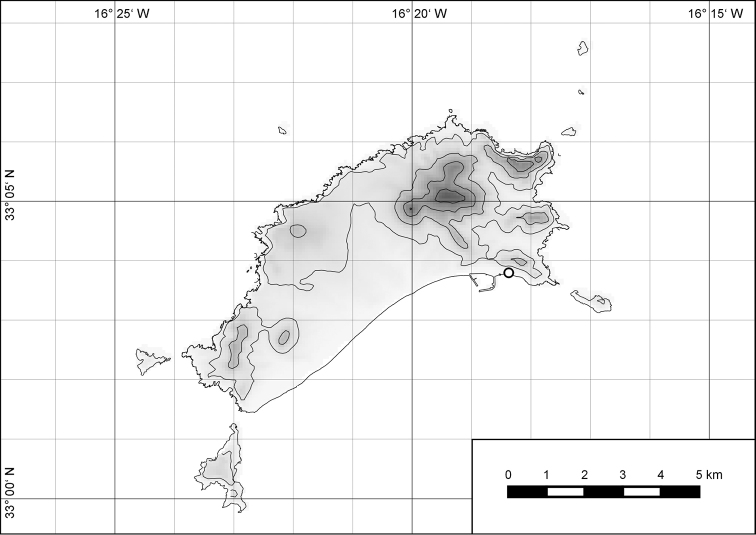
Distribution of *Wollastonia ripkeni* sp. n.

Known only from the locus typicus. The species was hitherto found only along a 50 m stretch of mud deposits close to the cart speedway. Additional deposits in the vicinity could not be checked for the presence of the species because of difficult accessibility of the steep slopes and associated risks of stone fall and landslides. The currently known distribution is shown in Fig. 125.

##### Etymology.

Named for the Dutch malacologist Theodor (“Theo”) J. E. Ripken from Delft, The Netherlands, to honour his valuable contributions to the malacofauna of the Macaronesian Islands.

##### Comparison and comments.

*Wollastonia ripkeni* sp. n. can be confused on first glance with other large-sized species such as *H. echinoderma*, *W. falknerorum* sp. n. or *W. vermetiformis*. From the first it is separated by the development of two distinct keels, from the second by a much finer sculpture, a narrower umbilicus and narrower aperture. From the quite similar *H. vermetiformis* it differs by a much more solid shell, a much higher last whorl, a larger aperture, finer granulation and coarser ribbing of the body whorl. From similar sized *W. subcarinulata* and *W. inexpectata* sp. n. it is separated by a coarser granulation and the presence of two keels.

##### Taxonomic remarks.

*Wollastonia ripkeni* sp. n. is included in the genus *Wollastonia* because it is similar to *W. oxytropis* in size and surface sculpture, considerably differing from *Hystricella* s. str. where the tubercles are bigger and less densely set. We are well aware that, due to the lack of anatomical and molecular data, the generic placement of this subfossil species is exclusively based upon shell features, with all the taxonomic limits that this might implicate.

##### Status and conservation.

Extinct before the islands’ scientific exploration in the 19^th^ century, possibly already before human settlement.

#### † Wollastonia falknerorum

Taxon classificationAnimaliaStylommatophoraGeomitridae

Groh, Neiber & De Mattia sp. n.

http://zoobank.org/644C8413-0AF0-41E8-B286-2E7C413FA043

Figs 126–128 , 129

##### Type material.

All from Porto Santo, holotype, SMF 348925, from loc. typ., leg. W. De Mattia & J. Macor, May 24 2015; SMF 348926/2 PT, NMW.Z.2016.013.00007/2 PT, NHMW 112142/1 PT, CKG/2 PT, CWDM/22 PT, from loc. typ., leg. W. De Mattia & J. Macor, May 24 2015; NMG No. 78-14764/2 PT [as *H. vermetiformis*, det. H. Waldén], Prainha & Serra de Fora, 33°03'57"N/16°17'49"W, 20 m, leg. A. De Noronha, before 1978; NMG No. 86-17153/4 PT [as *H. vermetiformis*, det. H. Waldén], Penedo, 33°03'38"N/16°19'50"W, 10 m, leg. J. J. De Sousa, before 1986; CWDM/9 PT, Barbinha, slightly S towards the tunnel, Quaternary mixed gravel slope deposit, 33°3'56"N/16°17'49"W, 20 m, leg. W. De Mattia & J. Macor, May 24 2015; CKG/9 PT, Vale do Touro, 800 m E of Vila Baleira, 60 m NW of oil tanks, Quaternary slope deposit from the 3^rd^ of 11 layers of tuffites with approx. 20% of stones, 33°03'49"N/16°19'21"W, 12–14 m, Jun. 18 1983, leg. K. & C. Groh; CJG/3 PT, Vale do Touro, 800 m E of Vila Baleira, 60 m NW of oiltanks, Quaternary slope deposit from the 3^rd^ of 11 layers of tuffites with approx. 20% of stones, 33°03'50"N/16°19'21"W, 12–14 m, leg. J. Gerber, K. Groh & J. Hemmen, Aug. 16 1985; CKG/3 PT + 2 juv. (no PT), Pico dos Maçarico, opposite to Porto de Abrigo, leg. K.-H. Beckmann, Jan. 17 1998; CFW 12179/1 PT, Ponta da Galé, W end of tunnel, 300 m towards port, 33°03'36"N/16°17'58"W, 25 m, leg. F. Walther, Apr. 1 2017; CFW 12178/10 PT + 4 juv. (no PT) + fragm. (no PT), Ponta da Galé, W end of tunnel, 100 m towards port, 33°03'38"N/16°17'51"W, 35 m, leg. F. Walther & E. M. Gryl, Apr. 1 2017; CFW 12177/13 PT + 6 juv. (no PT) + 1 fragm. (no PT), CKG/3 PT, Ponta da Galé, E end of tunnel, lower level [of slope deposits], coarse, red coloured gravel with large stones, 33°03'47"N/16°17'45"W, 30 m, leg. F. Walther, Apr. 1 2017; CFW 12180/34 PT + 11 juv. (no PT) + fragms (no PT), CKG/5 PT, E of Vila Baleira, end of Vale do Touro, (sub-)fossil [slope-]deposits, coarse, black gravel, 33°03'48"N/16°19'15"W, 20 m, leg. F. Walther, Apr. 5 2017; CFW 12181/21 PT + 4 juv. (no PT) + fragms (no PT), E of Vila Baleira, S-slope of the hill above Vale do Touro, W of the oil tanks, [(sub-)fossil slope-deposits of] red gravel, 33°03'47"N/16°19'26"W, 25 m, leg. F. Walther, Apr. 5 2017; ANSP H 11854/9 PT [partim, together with 7 *H. subcarinulata* and 2 *Discula cheiranticola*], Barbinha, 33°04'04"N/16°17'49"W, 8 m, leg. J. & C. Hemmen & K. & C. Groh, 1983.

##### Locus typicus.

Porto Santo, Pico do Baixo, E entrance of the tunnel, Quaternary mud deposit, 33°3'44"N/16°17'45"W, 20 m.

##### Diagnosis.

*Wollastonia* species with two keels on the body whorl, the upper keel only very weakly developed; suture overlapping; granulation very fine; ribbing of the body whorl rather coarse.

**Figures 126–128.**
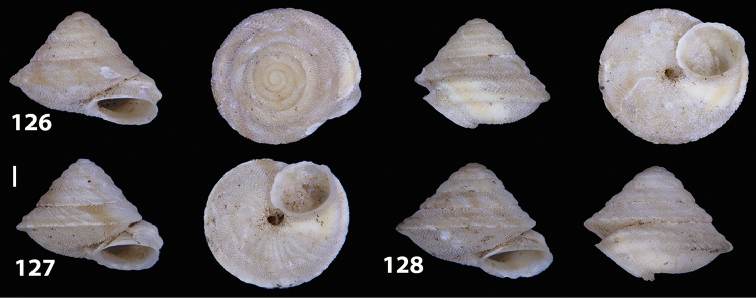
Shells of *Wollastonia falknerorum* sp. n. **126** holotype, SMF 348925127 **127, 128** paratypes from the loc. typ. Scale bar 1 mm.

##### Description of the holotype.

Shell large for the genus, with 6.5 regularly increasing whorls, the protoconch with 2.1 whorls. The form of the shell is conical, the convex teleoconch whorls show an upper, rounded angulation and a lower distinctly developed keel. The last whorl measures 60%, the penultimate whorl 10%, of the total shell height. The lower ¾ of the body whorl are beneath the keel, which is set off by a constriction above and below; shell below the lower constriction regularly convex in frontal view. The angulation of the body whorl is placed in the upper seventh of its total height and the keel in the upper fourth of its total height. The suture between the whorls is simple, but slightly overlapping the previous whorl. The aperture, which is inclined to the vertical axis of the shell in an angle of 54° and is descending in the last 5% of the last whorl in an angle of 42° to the horizontal axis, has an elliptic form, which is slightly narrowed towards the columella. Its width is 45%, of the total shell width and its height 31% of the total shell height. It has a slightly reflected lip, which is completely detached from the body whorl. The eccentric umbilicus, which measures 14% of the shell's total width, is in the last whorls circular and completely closed in earlier whorls. The protoconch is smooth, the teleoconch shows a number of oblique radial ribs, 9 in the penultimate quadrant of the body whorl and is additionally covered by numerous small, rounded tubercles. The number of tubercles in the standard-quadrate of the base is 135. There are traces of colouration in form of large blotches along the last two whorls (see Fig. 126).

##### Variation of the paratypes.

The shell shape is quite stable in all examined specimens (Figs 127–128).

##### Measurements.

D 8.4 ± 0.1 mm (range 8.2–8.5 mm); H 7.0 ± 0.3 mm (range 6.8–7.4 mm); FW 4.3 ± 0.2 mm; PA 42 ± 5.3°; DU 0.9 ± 0.09 mm; NT 68 ± 18; NW 6.5 ± 0.2 (*n* = 20). Ratio D/H 1.2; ratio FW/H 0.6.

##### Distribution.

**Figure 129.**
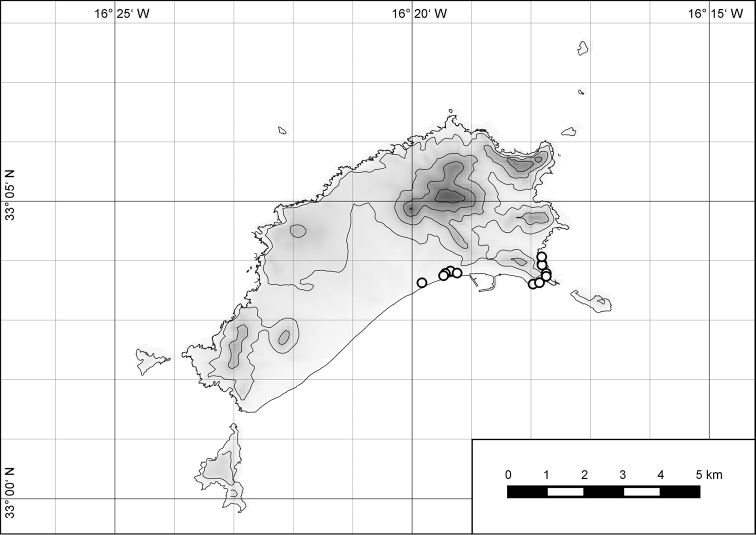
Distribution of *Wollastonia falknerorum* sp. n.

*Wollastonia falknerorum* sp. n. is found in the Quaternary mud and slope deposits along the southeastern coast of Porto Santo. Its range extends from the hills east of Vila Baleira to Barbinha, S of Porto dos Frades. The distribution is shown in Fig. 129.

##### Etymology.

Named for the German malacologists Margrit & Gerhard Falkner, Hörlkofen, to honour their highly valuable contributions to European malacology.

##### Comparison and comments.

*Wollastonia falknerorum* sp. n. can be confused on first glance with other comparatively large-sized geomitrid species like, *H. echinoderma*, *W. ripkeni* sp. n. and *W. vermetiformis*. From the first it is distinguishable by the presence of two distinct keels and from all three by a much finer sculpture. From the most similar *W. vermetiformis* and *W. ripkeni* sp. n. it is differentiated by the weakly developed upper keel, an overlapping suture, a much finer granulation and coarser ribbing of the body whorl. From similar-sized *W. subcarinulata* and *W. inexpectata* sp. n. it is separated by the two keels and the differently developed suture.

##### Taxonomic remarks.

*Wollastonia falknerorum* sp. n., as with other subfossil *Wollastonia* species, is included in this genus because it is similar to *W. oxytropis* in shell shape, size and surface sculpture, which differ considerably from *Hystricella* species. We are well-aware that, due to the lack of anatomical and molecular data, the generic position of this subfossil species is exclusively based upon shell features, with all the taxonomic limits that this might implicate.

##### Status and conservation.

Extinct before the islands’ scientific exploration in the 19^th^ century, possibly already before human settlement.

#### Wollastonia leacockiana

Taxon classificationAnimaliaStylommatophoraGeomitridae

(Wollaston, 1878) comb. n.

Figs 130–132 , 133–141 , 142

##### List of synonyms.

1878 Helix (Hystricella) leacockiana Wollaston: 165–167.

1894 *Geomitra leacockiana* – Pilsbry in Pilsbry 1893–1895: 34.

1931 Geomitra (Actinella) bicarinata var. leacockiana – Nobre: 87.

1950 Discula (Hystricella) leacockiana – Mandahl-Barth: 31, 55.

1983 Discula (Hystricella) leacockiana – Waldén: 20: 267.

2002 *Geomitra leacockiana* – Bank et al.: 124.

2008 *Hystricella leacockiana* – Seddon: 79–80, pl. 29 fig. A, map 179.

2011 *Hystricella leacockiana* – Seddon: e.T6720A12799605.

##### Type material.

NHM 1875.12.31.137-d, lectotype (herewith designated), from loc. typ., ex coll. T. V. Wollaston; NHM 1875.12.31.137-a to -c and -e, 4 paralectotypes, from loc. typ., ex coll. T. V. Wollaston.

##### Further material examined.

All from Porto Santo, CWDM/14, CMN/3, Pico de Ana Ferreira, northern slopes near the ‘lava columns’, under stones scattered in open grassy fields, 33°02'57"N/16°22'04"W, 125 m, leg. W. De Mattia & J. Macor, May 2014; CKG/32, CMN/9, Pico de Ana Ferreira, top of the hill, under stones scattered in open grassy fields, 33°02'39"N/16°22'11"W, 230 m, leg. K. & C. Groh & J. & C. Hemmen, Jul. 6 1983; CKG/20, CMN/10, E slope of Pico de Ana Ferreira, 33°02'37"N/16°22'05"W, 100–200 m, leg. K. & C. Groh, Oct. 26 1980; CKG/1, Ilhéu de Ferro, east side of the island plateau, under stones scattered in open grassy fields, 33°02'19"N/16°24'26"W, 75 m, leg. K. & C. Groh & J. & C. Hemmen, Jul. 2 1983; CFW 11151/<10, N slope of Pico de Ana Ferreira, near the basalt columns, 33°02'57"N/16°22'03"W, 125 m a.s.l., leg. F. Walther & E. M. Gryl, Mar. 30 2017; CFW 11152/<10 spms, Pico de Ana Ferreira, summit, 33°02'38"N/16°22'13"W, 230 m, leg. F. Walther, Apr. 4 2017; ANSP H 11838/c. 40, NW slope of the Pico de Ana Ferreira, 33°02'48"N/16°22'11"W, 190 m, leg. K. & C. Groh & J. & C. Hemmen, Jul. 6 1983; ANSP H 11770/ c. 30 [sub *H. bicarinata*], E slope of Pico de Ana Ferreira, 33°02'37"N/16°22'05"W, 100–200 m, leg. J. & C. Hemmen, Jan. 5 1981.

##### Locus typicus.

Portum Sanctum; in monte ‘Pico d’Anna Ferreira’ dicto sat copiose reperta. Necnon in statu semifossili (cum exemplaribus recentibus vix omnino congruens) parcissime occurrit.

##### Original description.

From Wollaston, 1878: T. trochiformis, subtus planata perforata, undique granulis obtusis densissime obsita, pallide brunneo-subflavescens sed fasciis (præsertim subtus) nebulisque irregiularibus (præsertim supra) rufo-brunneis hinc inde suffuse marmorata; spira sat elevata; anfractibus convexis, bicarinatis, ultimi (subtectiformis) carina exteriore acutissima valde exstanti, interior obtusa rotundata recedente rarius obsoleta; umbilico punctiformi; apertura subovali-rotundata, labris continuis conjunctis, peristomate simplici expanso subrecurvo tenui relevato. – Long. axis 1⅔ lin.; diam. 2½.

##### Redescription of the shell.

**Figures 130–132.**
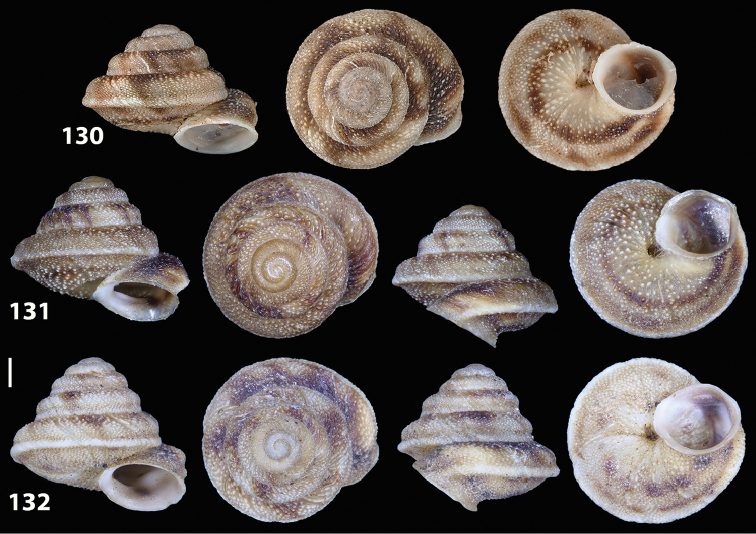
Shells of *Wollastonia leacockiana*. **130** lectotype of *Helix leacockiana* Wollaston, 1878, NMH 1875.12.31.137d, ex coll. Wollaston **131** Pico de Ana Ferreira **132** Ilheu de Ferro. Scale bar 1 mm.

The shell is dextral, hairless and it is usually conical and scalariform. The protoconch is from whitish to brown with 1.6 to 1.9 whorls. It is almost smooth along the first half whorl and shows fine radial striae and extremely small, scattered tubercles along its remaining portion. The teleoconch has from 3.9 to 4.4 rapidly increasing whorls. It is variable in colour, from very light brown to brown. The background colour is mottled with brownish to dark-reddish areas, irregularly arranged along the whorls. No band pattern is visible along the upper whorls. On the lower part of the last whorl two more or less indistinct, dark bands are visible. The peripheral band is usually the thinnest and often very indistinct. The spire is usually pyramidal and slightly scalariform. Along the last and partially along the penultimate whorl one evident keel is present. This keel extends along the lower part of the whorls and is usually lighter in colour, sometimes whitish. The external upper surface has very fine but clearly visible, irregularly spaced, growth lines. Irregularly disposed tubercles are present all over the teleoconch. The dimensions of the tubercles tend to slightly increase from the first towards the last whorl, but keeping approximately the same density. The tubercles are somewhat denser along the keel, letting the keel appear like a rough chord. The tubercles usually also concentrate somewhat along the growth lines. On the lower part, the tubercles are much denser along the periphery and gradually become scarcer towards the umbilicus. The last whorl is relatively large and descending near the aperture. The umbilicus is open but very narrow, concentric and measures approximately 10% of the maximum shell diameter. The aperture is elliptical with a quite strong thickening along the inner side of the last whorl. The peristome is continuous, slightly reflected with the columellar margin being somewhat thicker (see Figs 130–132).

##### Measurements.

D 5.3 ± 0.2 mm (range 5.0–5.6 mm); H 4.0 ± 0.3 mm (range 3.7–4.8 mm); FW 2.6 ± 0.2 mm (range 2.4–2.6); PA 58.8 ± 2.1° (range 55.0–60.0°); DU 0.5 ± 0.1 mm (range 0.4–0.6); NT 49 ± 5 (range 44–56); NW 5.4 ± 0.1 (range 5.3–5.5) (*n* = 40). Ratio D/H 1.3; ratio FW/H 0.6.

##### Body.

The head and neck are usually light grey, as is the posterior upper section of the foot. The foot is white and the sole is longitudinally divided into three areas. The central area is smooth, whereas the two lateral areas are equipped with bands of muscles roughly arranged in a chevron pattern. The mantle border is light grey, with five more or less developed lobes. The walls of the pallial cavity are colourless, without any stripes or spots. A strong pulmonary vein is visible. The jaw is odonthognatous and its shape is arched. There are 8 to 10 smooth transverse ridges. The right ommatophoral retractor is independent from both penis and vagina.

##### Genital anatomy.

**Figures 133–141.**
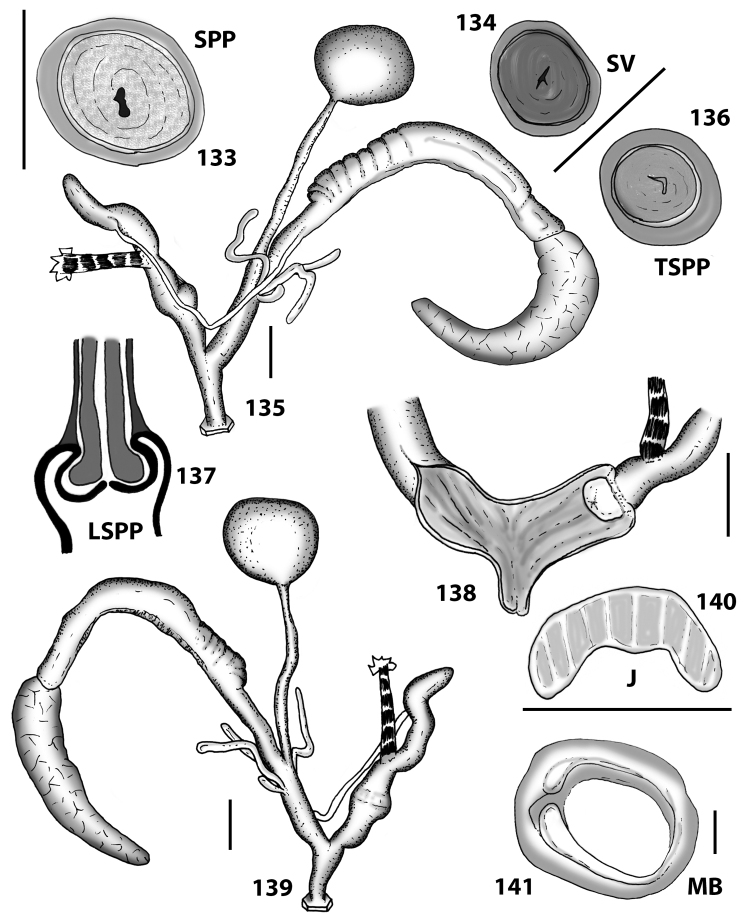
Genitalia and anatomy of *Wollastonia leacockiana*. Pico de Ana Ferreira, N slopes: **133** section of penial papilla **134** section of vagina **135** whole genitalia excluding gonads **136** tip of the penial papilla **137** longitudinal section of penial papilla **138** ornamentation of the inner walls of the distal penis, the distal vagina and the genital atrium **139** whole genitalia excluding gonads **140** jaw **141** mantle border. Scale bars 1 mm.

The albumen gland is long and it is connected to an approximately equally long sperm-oviduct. The thin vas deferens is approximately as long as the sperm-oviduct. The free oviduct is shorter than the vagina. The duct of the bursa copulatrix is usually thin, approximately as long as the penis and uniform in diameter. The bursa copulatrix is roundish. The transition area between the duct and the bursa itself is very sharply delimited, with the duct abruptly widening and turning into the bursa. The spermatophore is unknown. One tuft of a digitiform glands arises from the proximal part of the vagina. There are two or three glands that are equally long and never branched. A short and thin vaginal appendix arises from the vagina’s wall, just distal of the glandular tuft. The inner surface of the vagina is smooth. The atrium is usually long (¾ of the vagina) and relatively thin. Its internal walls are also smooth. The penial flagellum is short, remarkably cylindrical and with a blunt apex. It is usually as long as the epiphallus and its internal walls are completely smooth. The internal walls of the epiphallus are completely smooth as well. The retractor muscle is moderately long, strong and is of variable length. The penis lacks any muscular or glandular sheath. It is thick-walled and approximately three to four times longer than the flagellum. It is usually cylindrical, sometimes slightly swollen and partially folded up. The inner walls of the penis are smooth. The penial papilla is small and bulky. It has smooth external walls with the opening emerging apically but somewhat curving inwards. The channel of the penial papilla is thin and narrow and its cross section is ‘half-moon’-shaped. The inner lumen of the penial papilla is filled with a spongy and sturdy tissue, which directly connects with the walls of the epiphallus. As all the previous species, the longitudinal section of the penial papilla shows that its walls are the continuation of the penial walls that abruptly bend inward (see Figs 133–141).

##### Ecology.

*Wollastonia leacockiana* is commonly found under volcanic rocks and boulders scattered on grassland in open fields that are more or less sloping. The specimens aestivate on the lower surfaces of the rocks, frequently forming clusters of individuals attached one to another.

##### Distribution.

**Figure 142.**
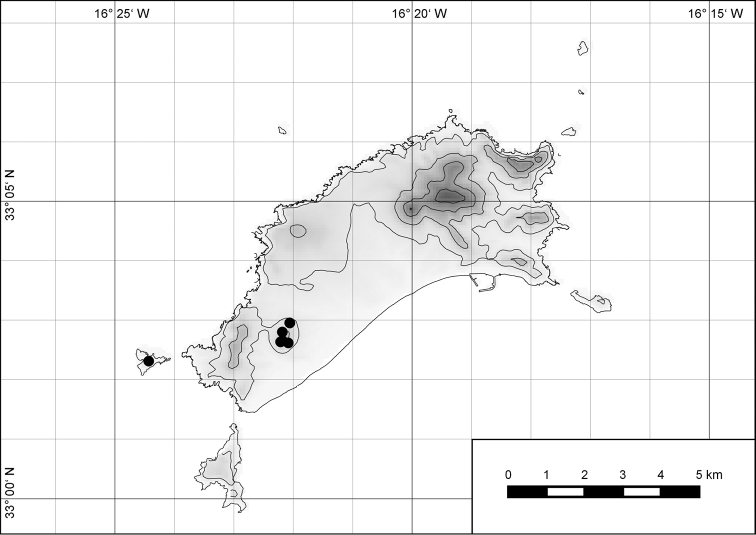
Distribution of *Wollastonia leacockiana*.

*Wollastonia leacockiana* is restricted to Pico de Ana Ferreira and Cabeco da Ponta at the western end of Porto Santo, with records also from Ilhéu de Ferro (CGK), although surveys are required to confirm if the species is still extant on that islet. Seddon (2008: 79, 182) reported the species from a spot at the eastern end of Porto Santo (Ponta do Passo area?). Despite intensive recent field research (WDM 2012, 2014, 2015) the species has not been confirmed in this area. Seddon’s (2008) record could represent a misidentification of a different species (cf. *W. klausgrohi* sp. n.). The currently known distribution is shown in Fig. 142.

##### Taxonomic remarks.

As reported by Seddon (2008: 79), Wollaston (1878: 161) separated *W. leacockiana* from *H. bicarinata* on the basis of its shell sculpture. This study confirmed Wollaston’s (1878) opinion, as do our molecular analyses and the investigation of the anatomy of the species.

##### Status and conservation.

The species is currently not significantly impacted by the construction of touristic/residential facilities in its distributional range; only in the Cabeco da Ponta area some new buildings were recently constructed. Recent surveys (WDM, 2012, 2014, 2015) showed that the species is currently widely distributed and common within its range. According to Seddon (2011c) the species is Vulnerable (VU D2), an assessment we regard as appropriate here because of the small range of the species and the potential threat of future construction works in the area. Further research is required to confirm the distribution of the species.

#### † Wollastonia beckmanni

Taxon classificationAnimaliaStylommatophoraGeomitridae

De Mattia & Groh sp. n.

http://zoobank.org/E0A0F760-57E6-4629-B09B-5FCCA78CCB13

Figs 143–145 , 146

##### Type material.

All from Porto Santo, SMF 348927, holotype, from loc. typ., leg. K. & C. Groh, June 6 1983; SMF 348928/5 PT, MMF 24956/1 PT, MMF 46281/1 PT, MMF 46282/1 PT, NHMW 112143/1 PT, CKG/62 PT, NMWZ 2016.013.00009/5 PT, CJG/5 PT, from loc. typ., leg. K. & C. Groh, June 6 1983; CWDM/12 PT, E of Vila Baleira, S slope of the hill above Vale do Touro, 50 m W of the oil tanks, excavated Quaternary mixed gravel, 33°03'47"N/16°19'26"W, 24 m, leg. W. De Mattia & J. Macor, May 24 2015; CFW 12176/5 PT, E of Vila Baleira, S slope of the hill above Vale do Touro, W of the oil tanks, [(sub-)fossil slope-deposits of] red gravel, 33°37'52"N/16°19'26"W, 25 m, leg. F. Walther, Apr. 5 2017; ANSP H 11843/2 PT [sub *H. leacockiana*], Vale do Touro, 33°03'46"N/16°19'29"W, 15 m a, leg. J. Gerber, K. Groh & J. Hemmen, Aug. 16 1985.

##### Locus typicus.

Porto Santo, south coast, ditch near the harbour built in 1985 at Vila Baleira, lens of quaternary calcareous aeolinite within a marine sandy beach terrace (nowadays beyond the quay of the harbour), 33°03'50"N/16°18'56"W, 0–1.5 m.

##### Diagnosis.

Small *Wollastonia* species with a very depressed body whorl that is finely granulated; umbilicus very wide in relation to maximum diameter, rather eccentric.

##### Description of the shell of the holotype.

**Figures 143–145.**
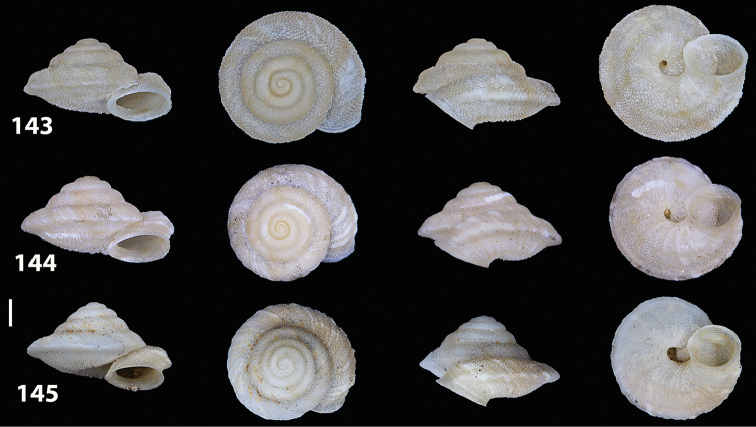
Shells of *Wollastonia beckmanni* sp. n. **143** holotype, SMF 348927 **144** paratype from the loc. typ. **145** Vale do Touro. Scale bar 1 mm.

Shell small for the genus, with 5.15 rapidly increasing whorls, the protoconch measures 1.5 whorls. The form of the shell is flat conical, the convex teleoconch whorls show a strongly keeled periphery. The last whorl measures 65%, the penultimate whorl 14% of the total shell height. The lower half of the body whorl is slightly concave beneath the keel in frontal view near the periphery and otherwise regularly convex. The suture between the whorls is simple and slightly impressed. The aperture, which is inclined to the vertical axis of the shell in an angle of 58° and descending in the last 5% of the last whorl in an angle of 43° to the horizontal axis, has an elongate-ovate form, its width measures 42% of the total shell width, its height 27% of the total shell height. It has a distinctly reflected lip, which is completely detached from the body whorl. The very eccentric umbilicus, which measures 17% of the total shell width, is in the upper whorls narrowly perspective. The protoconch is smooth, the teleoconch shows a low number of oblique radial ribs, nine in the penultimate quadrant of the body whorl and is additionally covered by many fine tubercles. The number of tubercles in the standard quadrate of the base is 102; the tubercles become coarser towards the centre of the base and finer towards the periphery. There are no traces of colouration (see Fig. 143).

##### Variation in paratypes.

The size varies slightly, and the density of the tubercles in the standard quadrate of the base may vary by ± 10%. Exceptionally, the umbilicus may be narrower and the angles of the aperture to the vertical axis and the descending of the last part of the body whorl to the vertical axis also slightly vary (see Measurements section below). Nevertheless, the overall shape and sculpture of all paratypes is very close to that of the holotype. In a few shells traces of a brownish colouration are perceptible, however there are no traces of a banding pattern (see Figs 144–145).

##### Measurements.

D 5.3 ± 0.2 mm (range 5.0–5.6 mm); H 3.3 ± 0.2 mm (range 3.2–3.5 mm); FW 2.2 ± 0.1 mm (range 2.1–2.3 mm); PA 72.0 ± 3.0° (range 66.0–76.0°); DU 0.4 ± 0.05 mm (range 0.3–0.4 mm); NW 6.2 ± 0.2 (range 6.0–6.4) (*n* = 20). Ratio D/H 1.6; ratio FW/H 0.7.

##### Distribution.

**Figure 146.**
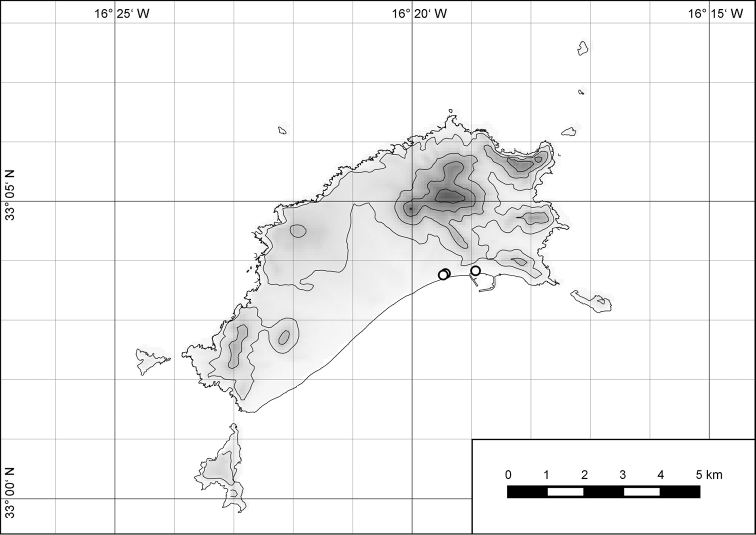
Distribution of *Wollastonia beckmanni* sp. n.

*Wollastonia beckmanni* sp. n. is known only from the southeastern coast of Porto Santo, from the hill immediately east of Vila Baleira (mud deposits at Vale do Touro) to the aeolinite deposits behind the new harbour. See map in Fig. 146.

##### Etymology.

Named after the late German entrepreneur and self-taught malacologist Dr. Karl-Heinz Beckmann (1948–2007) to honour his valuable contributions to the malacofauna of different islands and archipelagos in the Mediterranean and the Atlantic.

##### Comparison and comments.

Because of its small size the new species can only be confused with small *H. aucta* Wollaston, 1878, which have, however, a higher and more distinctly stepped spire, have two well-developed keels and much coarser and more sparsely set tubercles on the shell surface. *Hystricella microcarinata* sp. n. has a much higher, rounded conical form, two keels on the body whorl, coarser and less densely set tubercles, a much narrower umbilicus, and a different shape and differently positioned angles of the aperture.

##### Taxonomic remarks.

As with other subfossil *Wollastonia* species, *W. beckmanni* sp. n. is included in this genus because it considerably differs from *Hystricella* and is similar to *W. oxytropis* in surface sculpture and overall shell shape.

##### Status and conservation.

Extinct before the islands’ scientific exploration in the 19^th^ century, possibly already before human settlement.

#### Wollastonia jessicae jessicae

Taxon classificationAnimaliaStylommatophoraGeomitridae

De Mattia, Neiber & Groh sp. n.

http://zoobank.org/ADE24AD8-A30A-4171-992B-C7671B10902A

Figs 147–149

##### Type material.

NMWC Z.2016.013.00001, holotype, from loc. typ., leg. W. De Mattia & J. Macor, May 25 2015, NMWC Z.2016.013.00002/4 PT, SMF 348934/4 PT, NHMW 112140/3 PT, CKG/3 PT, CMN/7 PT, CWDM/23 PT, from loc. typ., leg. W. De Mattia & J. Macor, May 24 2015; FW 11154/10 PT, CMN/10 PT, ZMH 131207, 10 PT, hill E of Ribeira Santo Antonio and W of Vale do Touro, upper edge of the S slope, 33°03'47"N/16°19'41"W, 40 m, leg. F. Walther, Apr. 5 2017; CKG/5 PT, CJG/2 PT, Vale do Touro, under stones, 33°03'51"N/16°19'26"W, 45 m, leg. J. Gerber, K. Groh & J. Hemmen, Aug. 16 1985. Fossil: CKG/2 PT, Vale do Touro, Quaternary slope deposits, 33°03'47"N/16°19'26"W, 25 m, leg. J. Gerber, K. Groh & J. Hemmen, Aug. 16 1985. GNM 72-13.386/1 PT, Portela, 500 m SW, in the road cutting, Quaternary muds, 33°03'54"N/16°19'12"W, 95 m, leg. H. W. Waldén, Feb. 13 1972; ANSP H 11920/7 PT [with 8 spms of *H. aucta*], Vale do Touro, Quaternary slope deposits, 33°03'47"N/16°19'26"W, 25 m, leg. J. Gerber, K. Groh & J. Hemmen, Aug. 16 1985; ANSP H 11773/2 PT [with 3 *Caseolus compactus*], Vale do Touro, under stones, 33°03'51"N/16°19'26"W, 45 m, leg. J. Gerber, K. Groh & J. Hemmen, Aug. 16 1985.

##### Locus typicus.

Upper edge of the S slope of hill E of Ribeira Santo Antonio and W of Vale do Touro, under stones in grassland, 33°03'47"N/16°19'41"W, 40 m.

##### Diagnosis.

Small *Wollastonia* species, with a conical shell and two keels on the body whorl, the lower more distinct than the upper; granulation relatively fine and scattered; internal walls of penis with one to three well-developed, irregular pleats.

##### Etymology.

Named after the wife of the first author, Mrs. Jessica Macor from Trieste, Italy, as a token of gratitude for her assistance and companionship during the collecting activities.

##### Description of shell.

**Figures 147–149.**
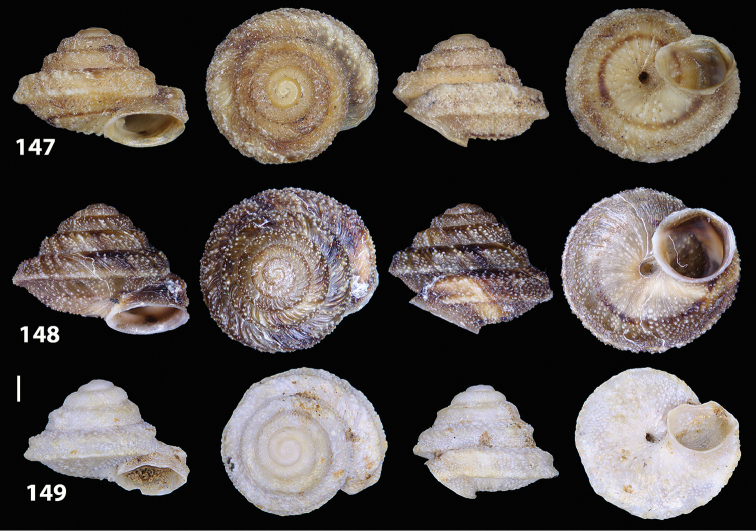
Shells of *Wollastonia jessicae jessicae* sp. n. **147** holotype, NMWC Z.2016.013.00001 **148** paratype from the loc. typ. **149** subfossil specimen from Vale do Touro. Scale bars 1 mm.

The shell is dextral and hairless. Its shape is scalariform, with deep sutures and rather flattened whorls. The protoconch is from yellowish to dark brown with 1.5 to 2.5 whorls. It is almost smooth along the first whorl and shows fine radial striae in its remaining whorls. The teleoconch has from 3.3 to 3.8 rapidly increasing whorls. It is usually dark brown with brick red and/or dark violet shades in colour, but also yellowish specimens with light reddish shades are found. The darker areas of the shell are mottled with more or less light brown to whitish areas, usually placed longitudinally and slightly slanting. The body whorl has two well-developed keels. The lower one is stronger and more distinct than the upper one. Both keels are usually whitish and contrasting from the rest of the body whorl. No banding pattern is visible along the upper whorls. On the lower part of the last whorl one principal, but rather narrow, dark (reddish to dark brown) band is usually present. A second light and indistinct band is sometimes present just below the keel. The area around the umbilicus is usually the lightest in colour. The external surface has strong, clearly visible, irregularly spaced, growth lines. Tubercles are present all over the teleoconch. They are usually large and rather widely scattered, whitish in colour and arranged somewhat obliquely, following the course of the growth lines. The larger tubercles are somewhat denser along the keel(s) of the last whorls, letting the keel(s) appear like a rough chord. The last whorl is usually large, with a contribution of 60% of the total shell height and descending near the aperture. The umbilicus is open but very narrow, either concentric or eccentric, and it measures approximately 10% of the maximum shell diameter. The aperture is elliptical with a faint thickening along the columellar portion of the stoma. Sometimes this thickening can also extend as far the parietal side of the aperture. The peristome is continuous and reflected (see Figs 147–149).

##### Measurements.

D 4.8 ± 0.3 mm (range 4.4–5.2 mm); H 3.3 ± 0.3 mm (range 3.0–3.9 mm); FW 2.3 ± 0.1 mm (range 2.2–2.4 mm); PA 58.2 ± 7.4° (range 50.0–68.0°); DU 0.7 ± 0.05 mm (range 0.6–0.8 mm); NT 23 ± 12 (range 9–37); NW 5.6 ± 0.4 (range 5.0–5.9) (*n* = 40). Ratio D/H 1.5; ratio FW/H 0.7.

##### Body.

The head and the neck are usually grey. The sides and the posterior upper section of the foot are whitish. The pigmented ommatophoral retractor muscles are visible through the skin of the back of the cephalic area. The foot is white and the sole is longitudinally divided into three areas. The central area is smooth, whereas the two lateral areas are equipped with bands of muscles that are roughly arranged in a chevron pattern. The mantle border is grey to dark grey, with five more or less developed lobes. The ratio of the lateral to the dorsal lobes varies from specimen to specimen, also in the same population. In some specimens, one of these lobes (either lateral or dorsal) may be totally missing. The walls of the pallial cavity are colourless, without any stripes or spots. A strong pulmonary vein is visible. The jaw is odonthognatous and is very variable in shape, ranging from almost straight to markedly arched. There are many smooth transverse ridges, ranging from 18 to 22 in number. The right ommatophoral retractor is independent from both penis and vagina.

##### Genital anatomy.

**Figures 150–157.**
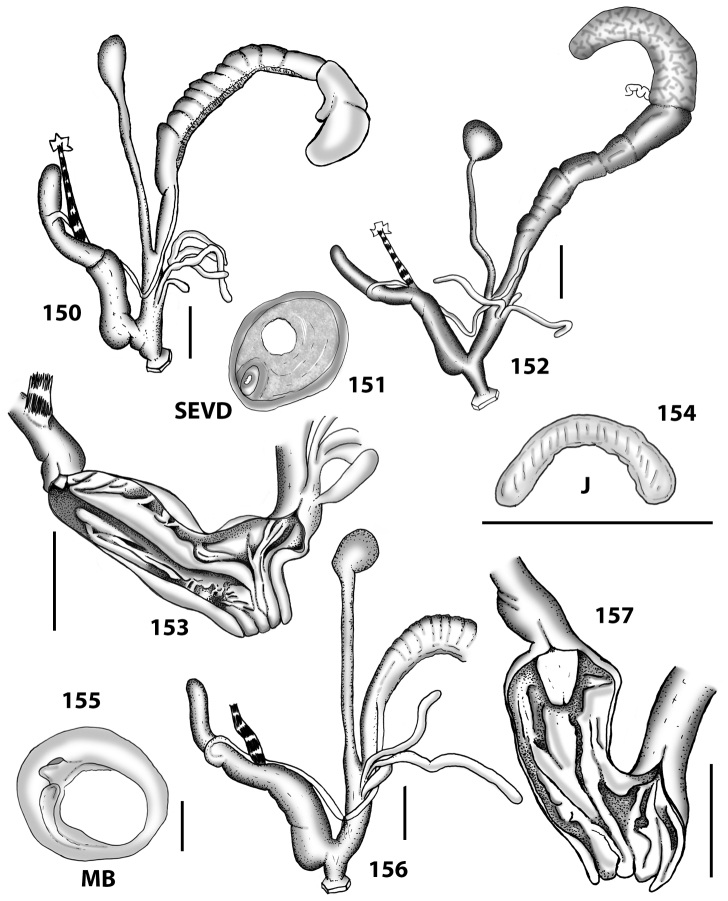
Genitalia and anatomy of *Wollastonia jessicae jessicae* sp. n. S slope of hill E of Ribeira Santo Antonio and W of Vale do Touro: **150** whole genitalia excluding gonads **151** section of proximal penis at the intersection with vas deferens **152** whole genitalia excluding gonads **153** ornamentation of the inner walls of the distal penis, the distal vagina and the genital atrium **154** jaw **155** mantle border **156** whole genitalia excluding part of OSD, AG and gonads **157** ornamentation of the inner walls of the distal penis, the distal vagina and the genital atrium. Scale bars 1 mm.

The general arrangement of the genitalia is semi-diaulic monotrematic. A convoluted to almost straight hermaphrodite duct arises from a multi-lobated gonad. The albumen gland is long and thin and is connected to an approximately twice as long sperm-oviduct that consists of a prostatic and a uterine portion. The prostatic part extends into a thin vas deferens, which is approximately as long as the sperm-oviduct and terminates in the penial complex. The distal portion of the uterine part extends into the free oviduct, turning into a vagina at the level of the duct of the bursa copulatrix. The free oviduct can be as long as, or slightly shorter than the vagina. The duct of the bursa copulatrix is usually thin, approximately as long as the penis and uniform in diameter. It ends into a variable, oval to roundish, bursa copulatrix. The transition area between the duct and the bursa itself is usually sharply delimited, with the duct abruptly widening and turning into the bursa. The spermatophore is unknown. One tuft of digitiform glands arises from the proximal part of the vagina. There are usually two glands that are approximately equally long and very rarely branched. A short and thin vaginal appendix arises from the vagina’s wall, just distal of the glandular tuft. Very smooth, rather wide and little elevated, irregularly spaced pleats run longitudinally along the inner surface of the vagina, reaching into the genital atrium as far as the genital orifice. The atrium is usually long and thin. Its internal walls have large and soft pleats running longitudinally as far the genital orifice. The penial complex consists of a flagellum, an epiphallus (which extends from the insertion of the vas deferens to the penial retractor muscle) and a penis, which inserts into the atrium. The penial flagellum is short, remarkably cylindrical and with a blunt apex. It is usually as long as the epiphallus. Its internal walls are completely smooth. The epiphallus is usually short and its internal walls present 2 to 4 longitudinal, smooth pleats that are only slightly elevated. The retractor muscle is not very large, but strong, usually as long as the flagellum + epiphallus together. The penis lacks any muscular or glandular sheath. It is thick-walled and approximately three times longer than the flagellum. It is usually slightly swollen in its distal part near the genital atrium. The inner walls of the penis usually have one to three irregular, spaced pleats, which run longitudinally and reach the genital atrium. These pleats can be connected by small “bridge-like” pleats. In some specimens, the section where the penial papilla is located is detectable from the outside by virtue of a fine circular swelling corresponding to the origin of the papilla itself. The penial papilla is usually very variable in dimensions (measuring from 10% to 50% of the total penial length) and conical in shape. It has smooth external walls, with the opening emerging apically. The channel of the penial papilla is thin and narrow. The inner lumen of the penial papilla is occupied by a spongy and sturdy tissue, which directly connects with the walls of the epiphallus. The longitudinal section of the penial papilla shows that its walls are the continuation of the penial walls that abruptly bend inward (see Figs 150–157).

##### Distribution.

**Figure 158.**
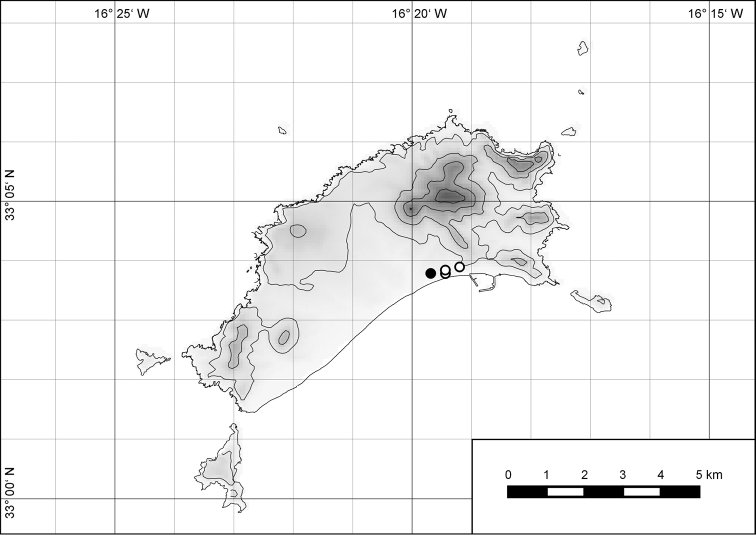
Distribution of *Wollastonia jessicae jessicae* sp. n. Filled circles refer to recent and open circles to fossil records.

*Wollastonia jessicae jessicae* sp. n. is found only along the southern slope of a hill east of Ribeira Santo Antonio and west of Vale do Touro, just east of the town of Vila Baleira, along the road leading to the new harbour. The distribution is shown in Fig. 158.

##### Ecology.

*Wollastonia jessicae jessicae* sp. n. is commonly found under volcanic rocks scattered on grassland in open fields that are more or less sloping. The specimens aestivate on the lower surfaces of the rocks, frequently forming clusters of individuals attached one to another.

##### Comparison and comments.

At first glance *W. jessicae jessicae* sp. n. can be confused with *H. bicarinata* because of the overall similarity of their shell’s shape (i.e. granulated surface with the last whorl with two keels). This is probably the reason why the new species has been overlooked until now. Nevertheless, a closer look reveals differences such as the much coarser granulation on the surface of *H. bicarinata*.

*W. jessicae jessicae* sp. n. (as for *W. jessicae monticola* sp. n. described in the following section) always shows a finer granulation, having smaller and more scattered tubercles. With regard to genital anatomy, *W. jessicae jessicae* sp. n. has internal walls of the penis with one to three irregular and well-spaced strong pleats, which run longitudinally and reach the genital atrium, whereas *H. bicarinata* has at least some large and smooth folds. Our phylogenetic analysis clearly show that the two species are not closely related, i.e. they were placed in two well-separated clades. This further corroborates, aside from morphology, its status as a distinct species.

##### Status and conservation.

The subspecies has a very limited distribution area of less than 1 km^2^ (Fig. 158) close to a village and population size is probably rather low. Habitat quality is inferred to be declining and potential and ongoing threats to the subspecies include, in our opinion, urbanization, tourism, goat grazing and quarrying. Therefore, the species is considered here to be Critically Endangered (CR B1a, b(iii), 2a, b(iii)).

#### Wollastonia jessicae monticola

Taxon classificationAnimaliaStylommatophoraGeomitridae

De Mattia, Neiber & Groh ssp. n.

http://zoobank.org/98F7562E-7017-4CAA-8DDA-91EA11C3DB54

Figs 159–161 , 162–166 , 167

##### Type material.

NMWC Z.2016.013.00010, holotype, from loc. typ., leg. W. De Mattia & J. Macor, May 21 2015, NMWC Z.2016.013.00011/4 PT, SMF 348931/4 PT, NHMW 1121341/3 PT, CKG/3 PT, CMN/3 PT, CWDM/6 PT, from loc. typ., leg. W. De Mattia & J. Macor, May 21 2015; FW 11155/5 PT, CMN/5 PT, ZMH 131208/4 PT, 200 m SW of the Zimbreiro near the road turn serpentine, 33°04'16"N/16°18'53"W, 100 m, leg. F. Walther, Mar. 31 2017; FW 11157/>10 PT, ridge between Zimbreiro and the quarry, 33°04'13"N/16°18'49"W, 110 m, leg. F. Walther, Apr. 3 2017.

##### Locus typicus.

Porto Santo, Zimbreiro, 200 m SW of the village, near the road turn serpentine, 33°04'16"N/16°18'53"W, 85 m.

##### Diagnosis.

Subspecies of *Wollastonia jessicae* with two keels on the body whorl, the lower distinct, the upper one very weakly developed to lacking; granulation relatively fine and scattered; internal walls of penis smooth, without pleats or prominent folds.

##### Etymology.

Named for the restriction of its habitat to the mountainous region (Lat. *mons*, *montis* = mountain(s) and -*cola* = inhabitor) of the island of Porto Santo.

**Figures 159–161.**
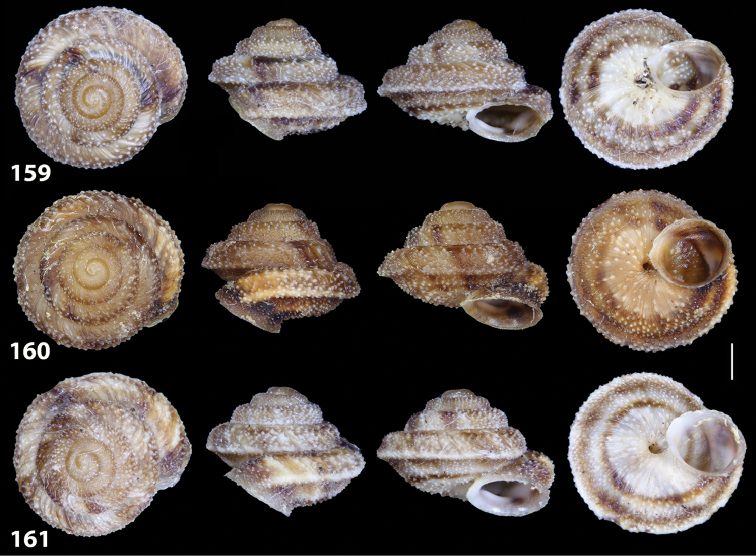
Shells of *Wollastonia jessicae monticola* ssp. n **159** holotype, NMWC Z.2016.013.00010 **160, 161** paratypes from the loc. typ. Scale bar 1 mm.

**Shell.** Similar to the nominate subspecies, except for the ornamentation of the body whorl. In *W. jessicae monticola* ssp. n. the upper keel is missing and the lower keel is somewhat less strongly developed and less evident compared to *W. jessicae jessicae* sp. n. (see Figs 159–161).

##### Measurements.

D 4.6 ± 0.2 mm (range 4.2–4.9 mm); H 3.1 ± 0.1 mm (range 2.8–3.6 mm); FW 2.0 ± 0.1 mm (range 1.9–2.3 mm); PA 58,6 ± 6.1° (range 51.3–65.7°); DU 0.7 ± 0.04 mm (range 0.6–0.8 mm); NT 25 ± 14 (range 8–39); NW 5.5 ± 0.3 (range 4.9–5.9) (*n* = 30). Ratio D/H 1.5; ratio FW/H 0.6.

##### Body.

The overall body colouration (i.e. head, neck and sides) of *W. jessicae monticola* ssp. n. tends to be somewhat darker than in the nominate subspecies.

##### Genital anatomy.

**Figures 162–166.**
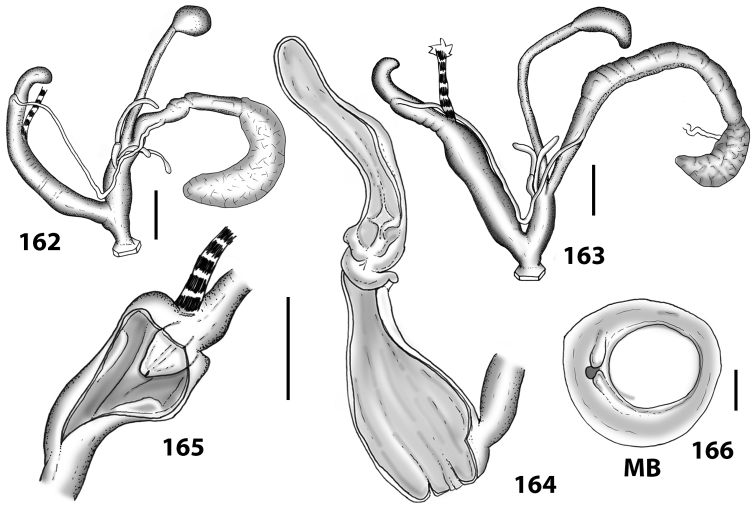
Genitalia and anatomy of *Wollastonia jessicae monticola* ssp. n. Zimbreiro, 200 m SW of the village: **162, 163** whole genitalia excluding gonads **164** ornamentation of the inner walls of the distal penis, the distal vagina and the genital atrium **165** penial papilla and inner ornamentation of proximal penis **166** mantle border. Scale bars 1 mm.

The distal genitalia of *W. jessicae monticola* ssp. n. are similar to those of the nominate subspecies, except for the inner ornamentation of the genital atrium and penis that is, contrary to *W. jessicae jessicae* sp. n., completely smooth, without any pleats or folds. Minor differences can also be found in the length of the vaginal digitiform glands that are, on average, longer in *W. jessicae monticola* ssp. n. (see Figs 162–166).

##### Ecology.

*W. jessicae monticola* ssp. n. lives in a sloping hollow, in a relative humid spot along a temporary creek. The new taxon has been found exclusively under small volcanic stones in shady places under small shrubs. Its ecology differs somewhat from all the other *Hystricella* and *Wollastonia* species on Porto Santo, which are usually found under stones in open, dry grassland areas or on exposed rocky cliffs. *W. jessicae monticola* sp. n. has been found syntopically with *Callina bulverii*.

##### Distribution.

**Figure 167.**
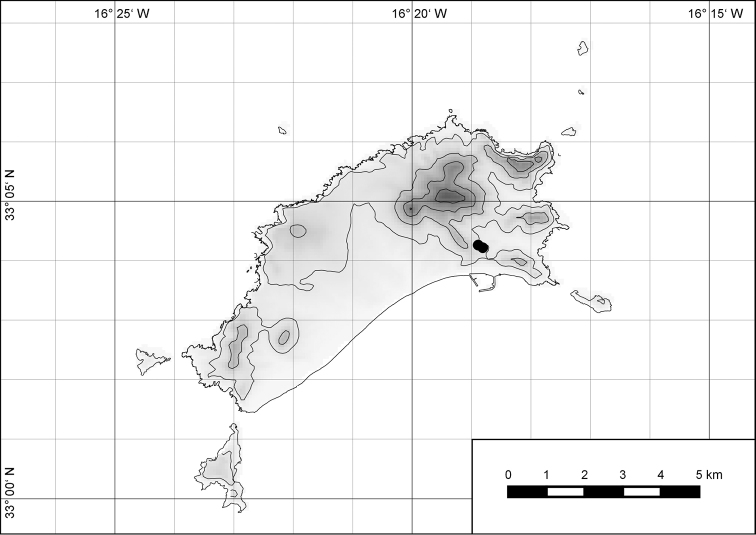
Distribution of *Wollastonia jessicae monticola* ssp. n.

*W. jessicae monticola* ssp. n. is only known from the area in the vicinity of the *locus typicus*. Despite extensive field research in the area, the subspecies seems to occupy an area not larger than 100 m^2^ (see Fig. 167 for the distribution map).

##### Comparison and comments.

The new taxon differs from the nominate subspecies *H. jessicae jessicae* sp. n. by the almost complete lack of the upper keel on the body whorl and by the smooth internal genital atrium and penis. There is also a significant distributional gap between the two conspecific taxa and the habitats and altitudinal ranges also differ. In the phylogenetic analyses, *W. jessicae monticola* ssp. n. represents the sister group of *W. jessicae jessicae* inside the *W. jessicae* s. lat. clade, but divergence is rather low which speaks for a relatively recent separation and may be taken as justification for the subspecific status of the two taxa.

##### Status and conservation.

The subspecies has a very limited distribution area of less than 1 km^2^ (Fig. 167) close to a village and population size is probably rather low. Habitat quality is inferred to be declining and potential and ongoing threats to the species include, in our opinion, urbanization, tourism, goat grazing and quarrying. Therefore, the subspecies is considered here to be Critically Endangered (CR B1a, b(iii), 2a, b(iii)).

#### Wollastonia klausgrohi

Taxon classificationAnimaliaStylommatophoraGeomitridae

De Mattia & Neiber sp. n.

http://zoobank.org/D4494EC4-F56E-4E01-BD51-FA18D1BEEB58

Figs 168–170 , 171–181 , 182

##### Type material.

NMWC Z.2016.013.00003, holotype, from loc. typ., leg. W. De Mattia & J. Macor, May 27 2015; NMWC Z.2016.013.00004/4 PT, SMF 348932/4 PT, NHMW 112139/3 PT, CKG/3 PT, CWDM/8 PT, CMN/4 PT, from loc. typ., leg. W. De Mattia & J. Macor, May 24 2015; FW 11156/25 PT, CMN/10PT, ZMH 131209/10 PT, Casa Velhas, next to the W edge of the old quarry, 33°04'06"N/16°18'51"W, 125 m, leg. F. Walther, Apr. 3 2017; CGK/3 PT, Portela, 33°03'58"N/16°19'03"W, 146 m, leg. K. & C. Groh, Oct. 25 1980; ANSP H 11784/8 PT, approx. 200 m SE of Casas Velhas, 33°04'04"N/16°18'47"W, 120 m, leg. J. & C. Hemmen & K. & C. Groh, Jul. 8 1983; ANSP H 11772/14 PT, approx. 50 m above Capela da Graça, 33°04'28"N 16°19'26"W, 165 m, leg. J. & C. Hemmen, Jan. 4 1981.

##### Locus typicus.

Casa Velhas, next to the W edge of the old quarry, under stones, 33°04'06"N/16°18'52"W, 124 m.

##### Diagnosis.

Small *Wollastonia* species, with two well-developed keels on the body whorl, the upper slightly less distinct than the lower; shell relatively dark coloured (in comparison with *W. jessicae*); internal walls of vagina, penis and genital atrium smooth, without folds.

##### Description of shell.

**Figures 168–170.**
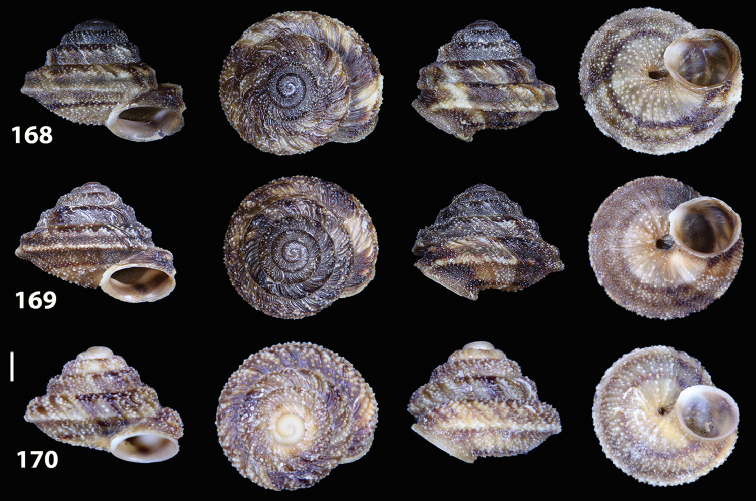
Shells of *Wollastonia klausgrohi* sp. n. **168** holotype, NMWC Z.2016.013.00003 **169, 170** paratypes from the loc. typ. Scale bar 1 mm.

The shell is dextral and hairless. Its shape is conical and markedly scalariform, with deep sutures. The protoconch is completely dark brown with 1.5 to 2.5 whorls. It has fine radial striae starting from the first protoconch whorl; very few and scattered small tubercles may also be present on its last portion. The teleoconch has from 3.4 to 3.9 rapidly increasing whorls. It is dark brown, sometimes with dark violet shades. The dark areas of the shells are mottled with more or less light brown to whitish areas, usually arranged longitudinally and slightly slanting. In most specimens, the lighter areas tend to be more evident along the keel of the body whorl. No band pattern is visible along the upper whorls. On the lower part of the last whorl two dark bands are present. The inner band is usually thinner but more evident, whereas the outer band is usually weaker but broader. The area around the umbilicus is the lightest in colour. Some specimens clearly have two evident keels starting already from the second whorl of the teleoconch, with the upper keel being more distinct than the lower one. In other specimens only the upper keel is visible. The body whorl is equipped with two keels, with the lower, principal keel somewhat protruding and the upper much less distinct. The whorls are flat and form a ‘shoulder’ giving them an angular contour. The external surface has strong, clearly visible, irregularly spaced, growth lines. Irregularly disposed tubercles are present all over the teleoconch. The tubercles are usually small and scattered, usually whitish in colour. The tubercles are somewhat denser along the keels of the penultimate and last whorl, letting the keel(s) appear like a rough, whitish chord. The last whorl is rather large, contributing 60% to the total shell height and descending near the aperture. The umbilicus is open but very narrow, eccentric, and measures approximately 10% of the maximum shell diameter. The aperture is elliptical, with a faint thickening along its columellar portion. This thickening also extends as far as the parietal side of the aperture. The peristome is continuous and distinctly reflected (see Figs 168–170).

##### Measurements.

D 5.3 ± 0.2 mm (range 5.1–5.5 mm); H 4.0 ± 0.2 mm (range 3.8–4.2 mm); FW 2.5 ± 0.1 mm (range 2.4–2.6 mm); PA 58.0 ± 3.4° (range 52.8–60.4°); DU 0.5 ± 0.07 mm (range 0.4–0.6 mm); NT 57 ± 17 (range 46–77); NW 5.6 ± 0.1 (range 5.5–5.7) (*n* = 25). Ratio D/H 1.3; ratio FW/H 0.6.

##### Body.

The head and neck are usually dark grey. The sides and the posterior upper section of the foot are grey. The foot is light grey and the sole is longitudinally divided into three areas. The central area is smooth, whereas the two lateral areas are equipped with bands of muscles that are roughly arranged in a chevron pattern. The mantle border is dark grey, with five more or less developed lobes. The ratio of the lateral and the dorsal lobes varies from specimen to specimen, also in the same population. In some specimens, one of these lobes (either lateral or dorsal) may be totally missing. The walls of the pallial cavity are colourless, without any stripes or spots. A strong pulmonary vein is visible. The jaw is odonthognatous and is very variable in shape, from almost straight to markedly arched. There are many smooth transverse ridges, ranging from 18 to 22 in number. The right ommatophoral retractor is independent from both penis and vagina.

##### Genitalia.

**Figures 171–181.**
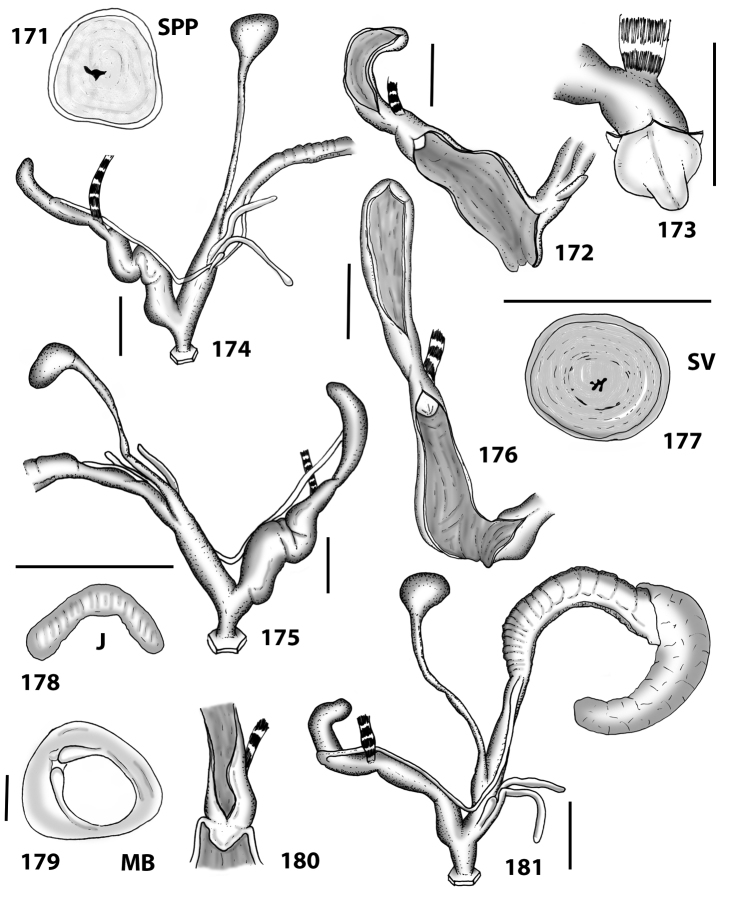
Genitalia and anatomy of *Wollastonia klausgrohi* sp. n. Casa Velhas, next to the W edge of the old quarry: **171** section of penial papilla **172** ornamentation of the inner walls of the flagellum, the penial complex, the vagina and the genital atrium **173** penial papilla **174, 175** whole genitalia excluding part of OSD, AG and gonads **176** ornamentation of the inner walls of the flagellum, the penial complex, the vagina and the genital atrium **177** section of vagina **178** jaw **179** mantle border **180** penial papilla **181** whole genitalia excluding gonads. Scale bars: 1 mm.

The albumen gland is long and connected to an approximately equally long sperm-oviduct that consists of a prostatic and a uterine portion. The prostatic part extends into a thin vas deferens that is approximately as long as the sperm-oviduct and inserts into the penial complex. The distal portion of the uterine part extends into the free oviduct, turning into a vagina at the level of the duct of the bursa copulatrix. The free oviduct is variable in length and can be as long as the vagina or also three times longer. The duct of the bursa copulatrix is usually thin, approximately as long as the penis and uniform in diameter. It terminates into a roundish bursa copulatrix. The transition area between the duct and the bursa is very sharply delimited, with the duct abruptly widening and turning into the bursa. The spermatophore is unknown. One tuft of digitiform glands arises from the proximal part of the vagina. There are usually two glands that are approximately equally long and are never branched. A short and thin vaginal appendix arises from the wall of the vagina, just distal of the glandular tuft. The inner surface of the vagina is almost smooth. The atrium is usually moderately long and thin. Its internal walls are smooth. The penial complex consists of a flagellum, an epiphallus (which extends from insertion of the vas deferens to penial retractor muscle) and a penis that inserts into the atrium. The penial flagellum is short, remarkably cylindrical and with a blunt apex. It is usually as long as the epiphallus. Its internal walls are completely smooth. The epiphallus is usually short. Its internal walls are also completely smooth. The retractor muscle is moderately large, strong and variable in length. The penis lacks any muscular or glandular sheath. It is thick-walled and approximately three to four times longer than the flagellum. It is usually cylindrical but sometimes slightly swollen and partially folded up. Sometimes, a thin sheath made of light connective tissue envelopes the distal folded part of the penis. The inner walls of the penis are smooth. The penial papilla is usually small, sometimes swollen at its base, reaching 1/8 to 1/10 of the total length of the penis and is conical in shape. It has smooth external walls, with the opening emerging apically. The channel of the penial papilla is thin and narrow, with somewhat fringed internal walls. The inner lumen of the penial papilla is occupied by a spongy and sturdy tissue, which directly connects with the walls of the epiphallus. As all the previous species, the longitudinal section of the penial papilla shows that its walls are the continuation of the penial walls that abruptly bend inward (see Figs 171–181).

##### Distribution.

**Figure 182.**
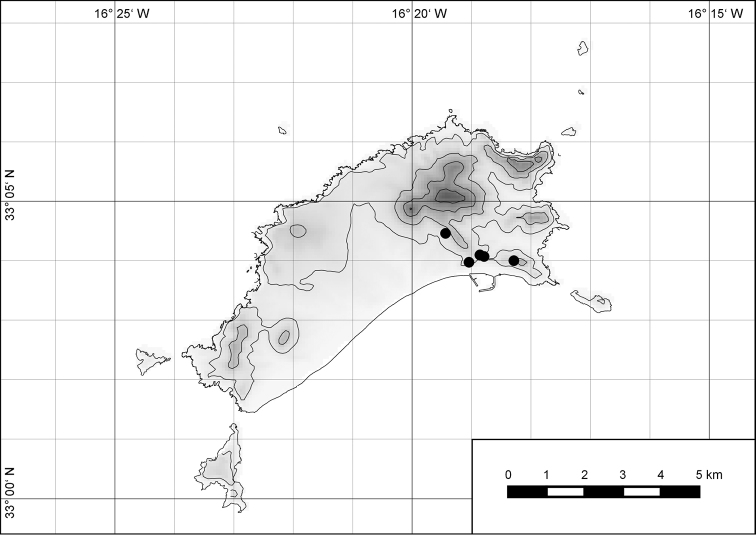
Distribution of *Wollastonia klausgrohi* sp. n.

*Wollastonia klausgrohi* sp. n. is found along the Casa Velhas area, just W of an abandoned quarry. The W tip of the quarry (currently not exploited at this part) is located only 10 meters from the type locality. Casas Velhas is located along the road n° 233 in direction of Zimbreiro, 340 m ENE from the Miradouro de Portela, E of Vila Baleira. The species has also been collected at Portela, ca. 300 m WSW of the loc. typ., Capela da Graça and 200 m SE of the type locality. The species seems to occupy less than 2,000 m^2^. For the distribution see Fig. 182.

##### Ecology.

*Wollastonia klausgrohi* sp. n. is commonly found under volcanic rocks of a low stone wall built in an open field in a sloping grassland. The specimens directly aestivate on the lower surfaces of the rocks, frequently forming clusters of individuals attached one to another.

##### Etymology.

Named for our co-author, the German malacologist Klaus Groh from Bad Dürkheim to honour his contributions to the malacology of continental snails on the Mid-Atlantic islands.

##### Comparison and comments.

**Figures 183–187.**
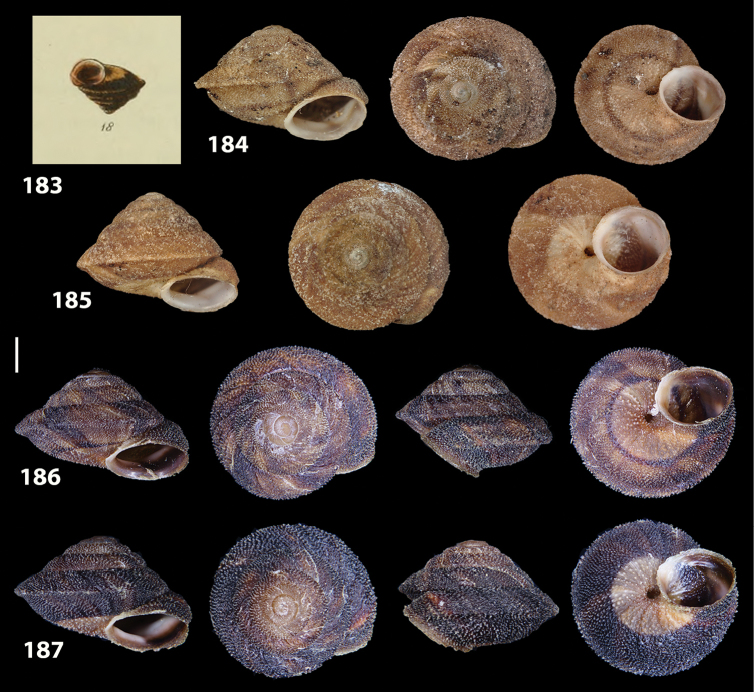
Shells of *Wollastonia oxytropis*. **183** original figure of *Helix oxytropis* R. T. Lowe, 1831 from Lowe (1831: pl. 6 fig. 18) **184** lectotype of *Helix oxytropis* R. T. Lowe, 1831, NMH 1968.546 ex coll. Lowe **185** paralectotype **186, 187** recent specimens from Pico do Concelho. Scale bar 1 mm.

**Figures 188–194.**
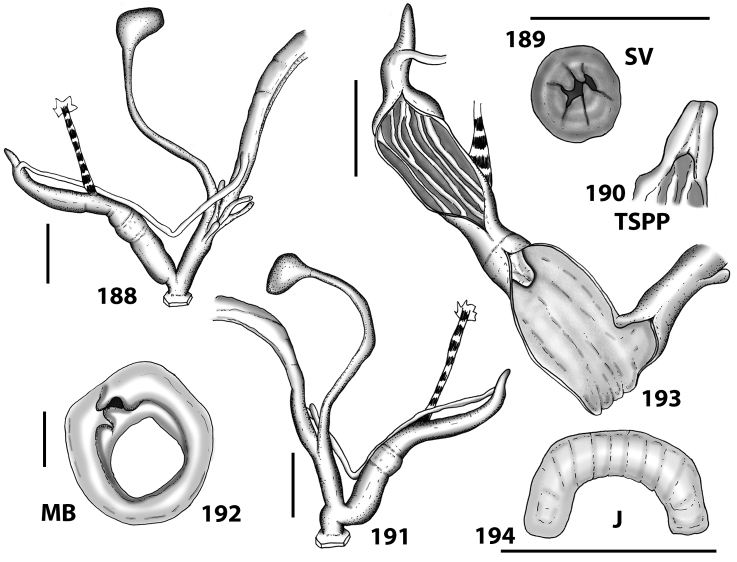
Genitalia and anatomy of *Wollastonia oxytropis* from Pico do Concelho: **188** whole genitalia excluding part of OSD, AG and gonads. Pico do Concelho: **189** section of vagina **190** tip of penial papilla **191** whole genitalia excluding part of OSD, AG and gonads **192** mantle border **193** ornamentation of the inner walls of the flagellum, the penial complex, the vagina and the genital atrium **194** jaw. Scale bars 1 mm.

*Wollastonia klausgrohi* sp. n. has a shell similar to *W. jessicae jessicae* sp. n. but differs from that species by a last whorl that is always bicarinate, with the upper keel well-visible. The overall colour of the shell is constantly darker in *W. klausgrohi* sp. n. More important differences are found in the genital anatomy. The internal walls of the penis, atrium and vagina of *W. klausgrohi* sp. n. are completely smooth, without any pleating or folding. It also resembles *H. bicarinata*, which, however, always has a wider and shorter atrium and a shorter vagina. Molecular investigations revealed that *W. klausgrohi* sp. n. is rather closely related to the morphologically very distinct *W. oxytropis*, whereas *W. jessicae* sp. n. is closely related to *W. leacockiana* that occurs in the western part of Porto Santo. Altough *W. klausgrohi* sp. n. was not recovered as a monophyletic group in the phylogenetic analyses (Fig. 5), statistical support values are rather low, so that monophyly can also not be excluded. We assume that the resolution power of the used molecular markers is insufficient to separate *W. klausgrohi* sp. n. and *W. oxytropis*. However, both taxa can easily distinguished by size, shell shape, ornamentation and genital anatomy (Figs 168–181, 183–194) and therefore are separated as distinct species here. Their distribution areas partly overlap, so that we can currently not exclude mitochondrial introgression as an explanation for the observed pattern in the phylogenetic tree in Fig. 5. Another explanation could be incomplete lineage sorting/ancestral polymorphism assuming a relatively recent divergence of the two taxa. An approach using population genetic markers could help to resolve these issues, such an approach is, however, beyond the scope of the present investigation.

##### Status and conservation.

Because of the very restricted distribution area, the low number of known subpopulations (Fig. 182) and the probably very small population size as well as potential threats through tourism, goat grazing and especially ongoing quarrying, the species is regarded as Critically Endangered (CR B1a, b(iii), 2a, b(iii)) here.

#### Wollastonia oxytropis

Taxon classificationAnimaliaStylommatophoraGeomitridae

(R. T. Lowe, 1831) comb. n.

Figs 183–187 , 188–194 , 195

##### List of synonyms.

1831 *Helix oxytropis* R. T. Lowe: 57, pl. 6 fig. 18.

1846 *Helix oxytropis* – L. Pfeiffer: 142, pl. 91 figs 12–13.

1847 *Helix oxytropis* – L. Pfeiffer in L. Pfeiffer 1847–1848: 190.

1854 *Helix oxytropis* – Reeve in Reeve 1851–1854: pl. 138 fig. 868.

1854 Helix (Ochthphila) oxytropis – Albers: 37, pl. 9 figs 8–10.

1855 Helix (Hystricella) oxytropis – R. T. Lowe: 186.

1867 Helix (Octephila) oxytropis – Paiva: 46.

1878 Helix (Hystricella) oxytropis – Wollaston: 167–168.

1888 *Helix oxytropis* – Tryon in Tryon and [Pilsbry] 1888: 33, pl. 7 fig. 92.

1894 *Geomitra oxytropis* – Pilsbry in Pilsbry 1893–1895: 242.

1931 Geomitra (Actinella) oxytropis – Nobre: pl. 2 fig. 3.

1950 Discula (Hystricella) oxytropis – Mandahl-Barth: 31, 55.

1983 Discula (Hystricella) oxytropis oxytropis – Waldén: 267.

2002 *Geomitra oxytropis oxytropis* – Bank et al.: 124.

2006 *Discula oxytropis* – Cameron et al.: 40 [partim].

2008 *Hystricella oxytropis* – Seddon: 79, map 180.

2009 *Hystricella oxytropis oxytropis* – Groh et al.: 21, fig. 26.

2011 *Hystricella oxytropis* – Seddon: e.T6728A12801442.

##### Type material.

NHM 1968.546, lectotype, (herewith designated), from loc. typ., ex coll. R. T. Lowe; NHM 1948.7.8.12/1 paralectotype, from loc. typ., ex coll. R. T. Lowe. See Fig. 183 for the original figure of *Helix oxytropis* R. T. Lowe, 1831 from Lowe (1831: pl. 6 fig. 18) and Figs 184–185 for the lectotype/paralectotype of *Helix oxytropis* R. T. Lowe, 1831 (Photo: P. Crabb, NHM).

##### Further material examined.

All from Porto Santo, CGK/1, CMN/1, Pico do Maçarico, under stones close to the top, 33°04'00"N/16°18'17"W, 200 m, leg. K. Groh & J. Hemmen, Jul. 10 1983; CKG/2, Pico do Maçarico, under stones close to the top, 33°04'00"N/16°18'17"W, 250–285 m, leg. K. Groh & J. Hemmen, Jul. 10 1983; CGK/2, CMN/1, Pico Novalido, W of Pico do Concelho, under stones, 33°04'43"N/16°18'19"W, 185 m, leg. K. Groh & J. Hemmen, Jun. 29 1983; CGK/5, Pico do Concelho, SW slopes, under stones, 33°04'37"N/16°17'47"W, 200–230 m, leg. K. Groh & J. Hemmen, Jun. 29 1983; CKG/2, slope of the Pico do Maçarico from Serra de Fora via Casas Velhas, 33°04'05"N/16°18'35"W, 100–200 m, leg. K. Groh & J. Hemmen, Jul. 10 1983; CKG/5, Pico do Baixo, W slope, 33°03'45"N/16°17'58"W, 150–210 m, leg. K. Groh & J. Hemmen, Jun. 9 1983; CWDM/25, ridge of Pico do Concelho towards E, under stones in grassland, 33°04'42"N/16°17'56"W, 270 m, leg. W. De Mattia & J. Macor, May 18 2015; CFW 11153/<10, Pico do Concelho, ridge W of the summit, 33°04'43"N/16°18'07"W, 220 m, leg. F. Walther, Apr. 3 2017; ANSP H 11846/10, CMN/2, Pico do Concelho, S slope, 33°04'41"N/16°17'57"W, 280 m, leg. J. Gerber, K. Groh & J. Hemmen, Aug. 12 1985; ANSP H 11845/3, Pico do Concelho, W slope, 33°04'43"N/16°18'04"W, 250 m, leg. K. Groh & J. Hemmen, Jun. 29 1983; ANSP H 11848/12, Pico Malhada, NW slope, 33°04'00"N/16°18'02"W, 120 m, leg. K. Groh & J. Hemmen, Jul. 7 1983; ANSP H 11851/12, Pico do Macaricos, 33°03'58"N/16°18'13"W, 230 m, leg. K. Groh & J. Hemmen, Jul. 10 1983; ANSP H 11850/3, Pico do Maçarico, SE slope, 33°03'57"N/16°18'11"W, 220 m, leg. J. & C. Hemmen, Jan. 8 1981; ANSP H 11849/7, Pico do Concelho, SW slope, 33°04'42"N/16°17'51"W, 200 m, leg. K. Groh & J. Hemmen, Jun. 29 1983; ANSP H 11849/14, approx. 0.5 km S Serra de Dentro, 33°04'44"N/16°18'26"W, 125 m, leg. J. & C. Hemmen, Jan. 6 1981; ANSP H 11852/9, Pico do Maçarico, NE slope, 33°03'58"N/16°18'09"W, 210 m, leg. K. Groh & J. Hemmen, Jul. 10 1983; ANSP H 11847/11, Pico do Baixo, 33°03'45"N/16°17'57"W, 150 m, leg. K. Groh & J. Hemmen, Jul. 9 1983; ZMH 24293/2, Porto Santo, without exact locality data, ex coll. Altonaer Museum; ZMH 24294/1, Porto Santo, without exact locality data, ex coll. Altonaer Museum, ex coll. O. Semper, ex coll. Dohrn.

##### Locus typicus.

Hab. in collibus maritimis Portus S^ti^.

##### Original description.

From Lowe 1831: H. testa depresso-conoidea, supra planulata, perforata, carinata, tota scabra, fusca, sub-fasciata: spira depresso-conica; sutura distincta; anfractibus planiusculis; ultimi carina acuta, distinctissima, supra marginata sc. exarata vel sulco expressa; omnibus distinctissime confertim granulatis, asperis: umbilico minimo, sub-spirali, aperto: apertura rotundata; peristomate continuo, circinato, disjuncto, reflexo. Axis 2 ¼ lin. Diam. 4. Anfr. 6 ½.

##### Shell.

The shell is dextral and hairless. Its shape is rather flattened, almost discoidal, with shallow sutures. The protoconch is brownish with 2–2.3 whorls. It is almost smooth along the first whorl and shows fine radial striae along its remaining portion. The teleoconch has from 3.3 to 3.8 rapidly increasing whorls. It is violet-dark brown with brick red and/or dark violet shades. The dark areas of the shells are mottled with more or less brown to whitish areas, usually arranged longitudinally and slightly slanting. No band pattern is visible along the upper whorls. On the lower part of the last whorl two dark bands are visible that can be more or less broad. The peripheral band is usually broader, even if often rather blurred. In some specimens, the two bands merge together and form a single broad, dark band. The area around the umbilicus is the lightest in colour. The teleoconch whorls are rather flat, with shallow sutures and without a visible keel. The body whorl shows a strong, single keel that sometimes even bends slightly downwards. This keel is of the same colour as the remaining shell and its ornamentation pattern is also not markedly different from that of the rest of the shell’s surface. Contrary to most other *Hystricella* and *Wollastonia* species, the keel does not have the appearance of a rough chord. The surface of the teleoconch is ornamented with very fine, irregularly spaced growth lines and regularly arranged, very densely set small tubercles. The last whorl is usually large, contributing approximately 50% to the total shell height and descending near the aperture. The umbilicus is open, eccentric, and measures approximately 10% of the maximum shell diameter. The aperture is elliptical, with a faint thickening along the columellar portion of the stoma. This thickening can also extend as far the parietal side of the aperture. The peristome is continuous, thin, slightly reflected, with the columellar margin somewhat thicker and more reflected (see also Figs 186–187).

##### Measurements.

D 6.8 ± 0.2 mm (range 7.5–8.0 mm); H 4.9 ± 0.4 mm (range 4.4–5.5 mm); FW 2.7 ± 0.2 mm (range 2.3–3.0 mm); PA 35.8 ± 6.9° (range 33.6–39.3°); DU 0.5 ± 0.05 mm (range 0.4–0.6 mm); NT 89 ± 14 (range 96–61); NW 5.6 ± 0.3 (range 5.3–5.9) (*n* = 25). Ratio D/H 1.4; ratio FW/H 0.6.

##### Body.

As in the genus description. *Wollastonia oxytropis* tends however, to have an overall darker body colouration than the other *Wollastonia* species.

##### Genital anatomy.

The albumen gland is long and thin and is connected to an approximately twice as long sperm-oviduct. The thin vas deferens is roughly 1.5 times longer than the penial complex. The free oviduct is as long as the vagina. The duct of the bursa copulatrix is thin, approximately as long as the penial complex and uniform in diameter. It ends in a roundish bursa copulatrix. The transition area between the duct and the bursa itself is sharply delimited, with the duct abruptly widening and turning into the bursa. The spermatophore is unknown. One tuft of digitiform glands arises from the proximal part of the vagina. There are usually three glands that are approximately equally long and very rarely branched. A short and thin vaginal appendix arises from the wall of the vagina, just distal of the glandular tuft. The internal walls of the vagina are smooth, as are those of the atrium. The atrium is relatively long and thin. The penial flagellum is very short, cylindrical, has a somewhat blunt to slightly pointed apex, and is usually much shorter than the epiphallus. The epiphallus is approximately as long to slightly longer than the penis. Its internal wall is equipped with irregular longitudinal pleats. The retractor muscle is rather large, strong and of variable length. The penis misses any muscular or glandular sheath. It is thick-walled, cylindrical, and slightly swollen at the level of the penial papilla. The inner walls of the penis are smooth. The penial papilla is small and it usually has a blunt shape. It has smooth external walls with the opening emerging apically. The channel of the penial papilla is thin and narrow. The inner lumen of the penial papilla is occupied by a spongy and sturdy tissue which directly connects with the walls of the epiphallus. The longitudinal section of the penial papilla shows that its walls are the continuation of the penial walls that abruptly bend inward. See Figs 188–194.

##### Ecology.

*Wollastonia oxytropis* is found under volcanic stones and rocks, in rock crevices and stone walls built in open, stony fields in sloping grasslands.

##### Distribution.

**Figure 195.**
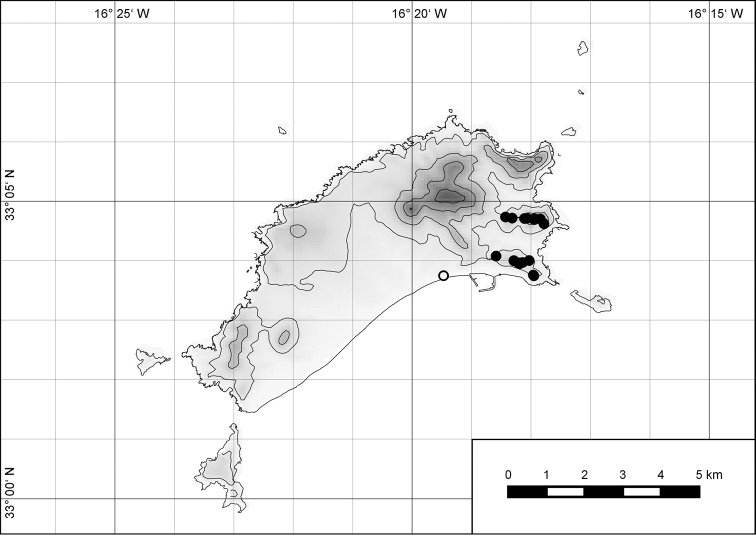
Distribution of *Wollastonia oxytropis*. Filled circles refer to recent and open circles to fossil records.

*Wollastonia oxytropis* has a quite small distributional range. It is known to live exclusively along the SE part of the main island Porto Santo: Pico do Baixo, Pico do Maçarico, Pico do Concelho and Pico do Novalido. These localities were confirmed by survey performed during the 1980’s and 1990’s (CKG, ANSP, Seddon 2008). During recent field surveys (WDM 2012, 2014, 2015, FW 2017) the species has been found living only along the ridge of Pico do Concelho despite careful searches at the above-mentioned localities. The known distribution is shown in Fig. 195.

##### Comparison and comments.

*Wollastonia oxytropis* is clearly distinguishable from all other species belonging to *Hystricella* and *Wollastonia* by the presence of only a single keel, the rather flattened, almost discoidal shell with very shallow sutures and the extremely short flagellum in relation to the epiphallus.

##### Taxonomic remarks.

*Wollastonia oxytropis* is somewhat unusual among the *Wollastonia* gen. n. species as its genital anatomy is more similar to that of the investigated *Discula* s. str. species than to that of the remaining *Wollastonia* gen. n. species. However, in the *cox1* phylogeny (Fig. 5) the species is clearly embedded within the *Wollastonia* gen. n. clade indicating that the similarity in genital anatomy may be the result of convergence. We therefore include the species in *Wollastonia* gen. n. until more in depth phylogenetic analyses and morphological comparisons of the remaining geomitrinid genera will become available.

##### Status and conservation.

According to the current IUCN assessment (Seddon 2011d) the species is considered Near Threatened (NT). Recent surveys indicate however that the species is clearly declining, both with regard to the distributional range and population size, probably as a result of a decline in habitat quality. The area of occupancy and extent of occurrence is approximately 4 km² and the species occurs at relatively few localities clustered in the upper parts of two hills in the south-eastern part of Porto Santo. Therefore, the species should be considered as Endangered (EN B1a, b(iii), 2a, b(iii)).

#### † Wollastonia subcarinulata

Taxon classificationAnimaliaStylommatophoraGeomitridae

(Wollaston, 1878), comb. n. stat. n.

Figs 196–198 , 199

##### List of synonyms.

1878 Helix (Hystricella) oxytropis var.ß subcarinulata Wollaston: 168.

Discula (Hystricella) oxytropis subcarinulata – Waldén: 267.

2002 *Geomitra oxytropis subcarinulata* – Bank et al.: 124.

2006 *Discula oxytropis* – Cameron et al.: 49 (1): 40 [partim].

2009 *Hystricella oxytropis subcarinulata* – Groh et al.: 21, fig. 27.

##### Type material.

**Figures 196–198.**
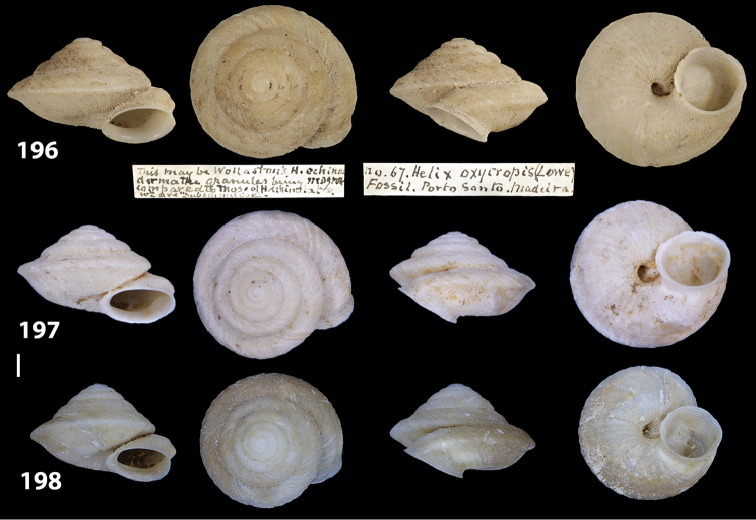
Shells of *Wollastonia subcarinulata*. **196** lectotype, NMWC 80.202, Acc 55.158; **197–198** specimens from Barbinha, Quaternary aeolinites. Scale bar 1 mm.

NMWC 80.202, Acc 55.158, lectotype (herewith designated), from loc. typ., ex coll. Melvill-Tomlin, ex coll. T. V. Wollaston, Porto Santo, Madeira (Fig. 196).

##### Further material examined.

All from Porto Santo, CKG/2, Barbinha, Quaternary aeolinites, layer 3, 33°04'04"N/16°17'49"W, 8 m, leg. K. & C. Groh, Jul. 4 1983 and Jul. 5 1983; CKG/3, Barbinha, Quaternary aeolinites, layer 2, 33°04'04"N/16°17'49"W, 8 m, leg. K. & C. Groh, Jul. 4 1983 and Jul. 5 1983; CKG/8, Barbinha, Quaternary aeolinites, layer 4, 33°04'04"N/16°17'49"W, 8 m, leg. K. & C. Groh, Jul. 4 1983 and Jul. 5 1983; CKG/2, south coast, ditch at the in 1985 new built harbour E Vila Baleira, lens of Quaternary calcareous aeolinite within a marine sandy beach terrace (nowadays beyond the quay of the harbour), 33°03'50"N/16°18'56"W, 0–1.5 m, leg. J. Gerber, K. Groh & J. Hemmen, Aug. 1985; CWDM/8, Porto Santo, excavated mud walls behind the cart speedway E of the new harbour of Porto Santo, 33°03'48"N/16°18'22"W, 30 m, leg. W. De Mattia & J. Macor, May 24 2015; CWDM/9, E of Vila Baleira, S slope of the hill above Vale do Touro, 50 m W of the oil tanks, excavated Quaternary mixed gravel, 33°03'47"N/16°19'26"W, 24 m, leg. W. De Mattia & J. Macor, May 24 2015; CWDM/20, Pico do Baixo, E entrance of the tunnel, Quaternary mud deposit, 33°03'44"N/16°17'45"W, 20 m, leg. W. De Mattia & J. Macor, May 24. 2015.

##### Locus typicus.

Porto Santo, Madeira.

##### Original description.

From Wollaston 1878: var. β. *subcarinulata* – Major, spira magis elevata, ad apicem paulo magis acuta, anfractibus in medio obsolete subcarinulato. – Long. axis 2½ lin.; diam. 3½.

##### Redescription of shell.

Shell large for the genus, with 5.9 regularly increasing whorls, the protoconch with 2.5 whorls. The form of the shell is flat conical, the slightly convex teleoconch whorls separated by a rather shallow but distinct, simple suture. The body whorl shows a distinct, rounded keel, which is enforced by a concave impression below the periphery. The last whorl measures 70%, the penultimate whorl 10% of the total shell height. The lower part of the body whorl is beneath the significantly marked periphery in frontal view first significantly concave, later moderately convex. The keel of the body whorl is located in the upper part of its total height. The aperture, which is inclined to the vertical axis of the shell in an angle of 69° and is descending in its last 5% in an angle of 36° to the horizontal axis, is horizontally elliptic; towards the umbilical region a bit flattened. Its width measures 45% of the total shell width and its height 44% of the total shell height. It has a peristome that is completely detached from the body whorl. The lip is distinctly reflected and very wide at its basal and columellar sides. The eccentric umbilicus, which measures 13% of the total shell width, is shaped like a pinhole in the penultimate whorl and is completely closed in the earlier whorls. The protoconch is smooth, the teleoconch shows a number of oblique radial ribs, 10 in the penultimate quadrant of the body-whorl and is additionally covered by numerous small, round tubercles. The number of tubercles in the standard-quadrate of the base is 108. There is a yellowish hue of colouration in the teleoconch; the keel usually distinctly lighter in colour. See Figs 197–198.

##### Measurements.

D 8.3 ± 0.3 mm (range 8.0–8.8 mm); H 6.1 ± 0.3 mm (range 5.8–6.4 mm); FW 4.1 ± 0.2 mm (range 3.9-4.2 mm); PA 48.2 ± 5.4° (range 42.6–54.4°); DU 0.6 ± 0,06 mm (range 0.5–0.7 mm); NT > 100; NW 5.6 ± 0.1 (range 5.5–5.7) (*n* = 25). Ratio D/H 1.4; ratio FW/H 0.5.

##### Distribution.

**Figure 199.**
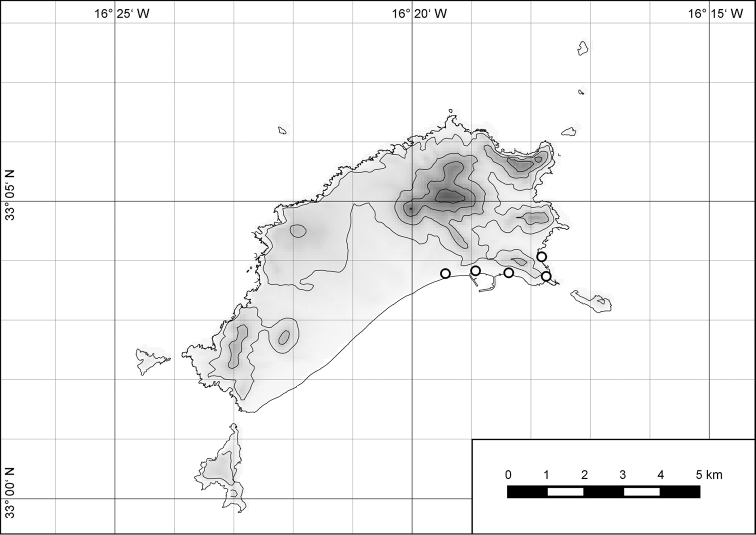
Map of the distribution of *Wollastonia subcarinulata*.

*Wollastonia subcarinulata* is known from the Quaternary mud and slope deposits along the southern slopes of the eastern part of Porto Santo, from Vale do Touro to the area around the tunnel of Ponta da Galé beneath Pico do Baixo. See map in Fig. 199.

##### Comparison and comments.

*Wollastonia subcarinulata* can be confused with other, rather large-sized species of the genera *Hystricella* and *Wollastonia* like *H. echinoderma*, *W. vermetiformis* and *W. falknerorum* sp. n., from which it can be distinguished, however, by the lack of a second keel, the rounded shape of the keel and a much finer sculpture as well as a more depressed conical form. From similar sized *W. inexpectata* it is distinguishable by the wider umbilicus, a flatter form, the keeled instead of angled periphery and a coarser granulation. From the recent *W. oxytropis* it can be distinguished by the larger size, the wider umbilicus, the more convex teleoconch whorls that are separated by a more distinctly marked suture, the finer granulation, and the wider aperture.

##### Taxonomic remarks.

The species is included in the genus *Wollastonia* because of its size and surface sculpture that is similar to *W. oxytropis*, as a variety of which it was described by Wollaston (1878). Subsequent authors, except Cameron et al. (2006), recognised the taxon as a subspecies of *W. oxytropis*.

##### Status and conservation.

Extinct before the islands’ scientific exploration in the 19^th^ century, possibly already before human settlement.

#### † Wollastonia inexpectata

Taxon classificationAnimaliaStylommatophoraGeomitridae

De Mattia & Groh sp. n.

http://zoobank.org/AD8D733D-8248-47EF-B9D8-CF9A3F831437

Figs 200–202 , 203

##### Type material.

**Figures 200–202.**
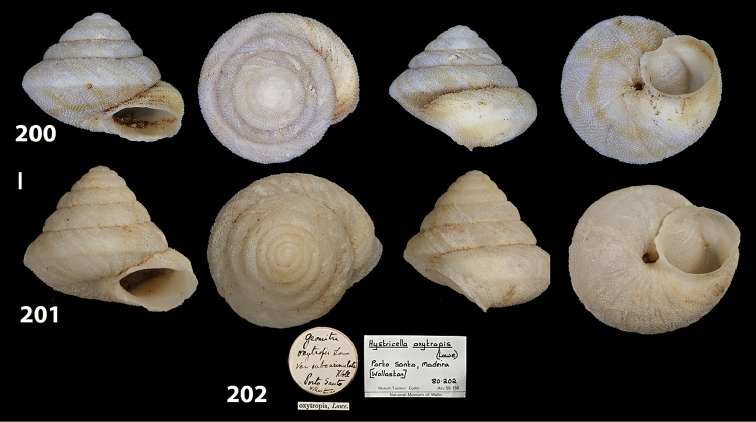
Shells of *Wollastonia inexpectata* sp. n. **200** holotype, SMF 348930 **201** paratype NMWC 80.202 Acc. 55 158 **202** label of the lot NMWC 80.202 Acc. 55 158. Scale bar 1 mm.

SMF 348930, holotype, from loc. typ., leg. W. De Mattia, May 23 2015 (see Fig. 200); NMWC 80.202 Acc. 55 158/1 PT as “Geomitra oxytropis Lowe var. subcarinulata Woll. Porto Santo” (Figs 201–202).

##### Locus typicus.

N of airport, end of the runway towards Fonte de Areia, Quaternary calcareous sand deposit, 33°05'25"N/17°20'58"W, 99 m.

##### Etymology.

*Wollastonia inexpectata* sp. n. was unexpectedly found during intensive field researches at Fonte da Areia when looking for *H. echinoderma*.

##### Diagnosis.

Shell large for the genus, solid and conical. Whorls rounded, with deep sutures. The shell’s surface covered with small and densely set tubercles. Body whorl with a peripheral, rounded keel. Other teleoconch whorls without visible keel. Umbilicus narrow but open. Last whorl descending toward the aperture. Aperture oval with continuous peristome.

##### Description of the shell.

The shell is large for the genus and rather conical, with 6.3 regularly increasing whorls. The protoconch has 1.7 smooth whorls. The teleoconch whorls are distinctly convex and separated by a slightly impressed but simple suture. The body whorl measures 64% and the penultimate whorl 14% of total shell height and is descending towards the aperture in its last 5% in an angle of 36°. It has a rounded angulation in its upper third (in relation to total shell height) that is emphasised by a narrow, only slightly concave impression below the periphery. The lower part of the body whorl (beneath the periphery in frontal view) is otherwise rather convex. The aperture is regular elliptical, measuring 49% of the total shell width and 29% of the total shell height. It is inclined to the vertical axis of the shell in an angle of 57°. The peristome is completely detached from the body whorl, expanded and distinctly reflected, especially in its basal and columellar part. The umbilicus is eccentric, closed in the early whorls, but pinhole-like in the body whorl, measuring 5% of the maximum shell diameter. The sculpture of the teleoconch consists of oblique radial ribs (10 in the penultimate quadrant of the body whorl) and numerous, small roundish tubercles (132 in the standard square basal surface of the shell). The colour is only preserved as a yellowish hue on the teleoconch, a slightly darker yellowish, narrow spiral band in the middle of the base and a lighter marked keel. See Figs 200–202.

##### Measurements.

D 8.6 mm; H 6.7 mm; FW 4.6 mm; PA 41.2°; DU 0.6 mm; NT 132; NW 6.6 (*n* = 1). Ratio D/H 1.3; ratio FW/H 0.7.

##### Distribution.

**Figure 203.**
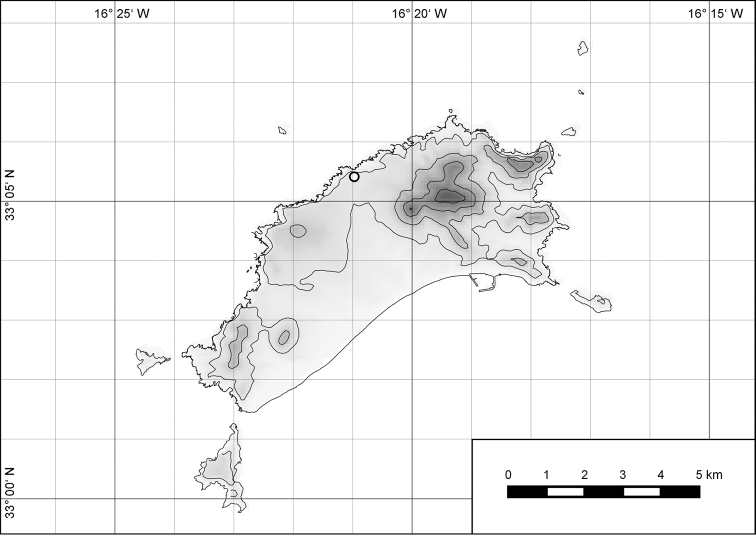
Distribution of *Wollastonia inexpectata* sp. n.

The species is only known from the type locality. A second specimen is known from another unidentified locality in Porto Santo and housed at the NMW. The distribution is shown in Fig. 203.

##### Comparison and comments.

*Wollastonia inexpectata* sp. n. is superficially similar to *H. echinoderma*, or *W. vermetiformis* and *W. falknerorum* sp. n. but is readily distinguishable from these species by the lack of a second keel, its much finer sculpture, narrower umbilicus and regular conical form. From *H. echinoderma* it can also be distinguished by its regular convex and not stepped whorls and the regular elliptical rather than oblique ovate aperture. From the similarly sized *W. subcarinulata* it differs in the narrower umbilicus, the presence of a rounded keel, the finer granulation and the higher shell in relation to its width.

##### Taxonomic remarks.

*Wollastonia inexpectata* sp. n. is included in the genus *Wollastonia* because it is similar to *W. oxytropis* in size and surface sculpture. It is noteworthy that the similarly large-sized *Hystricella echinoderma* most probably also originates from the Quaternary deposits in the north of the island, and likewise is extremely rare there.

##### Status and conservation.

Extinct before the islands’ scientific exploration in the 19^th^ century, possibly already before human settlement.

#### Callina

Taxon classificationAnimaliaStylommatophoraGeomitridae

R. T. Lowe, 1855

Helix (Callina) , Wollaston (1878).
Helix (Tectula) , sensu Albers (1854) and Paiva (1867), partim.
Geomitra (Actinella (Callina)), Pilsbry (1893–1895), partim.
Discula (Callina) , Mandahl-Barth (1950), Seddon (2008) and Bank et al. (2002).
Callina , Waldén (1983) and Schileyko (2005).

##### Type species.

*Helix* [*Helicella*] *rotula* R. T. Lowe, 1831 by monotypy.

##### Description of the genus.

**Shell.** The shell is dextral, solid, hairless, and it is usually discoidal to tectiform in shape. The protoconch is dark brown, with 1.4 to 1.9 whorls. It is almost smooth along the first whorl and shows radial striae along its remaining portion. The teleoconch has from 5.3 to 6.6 rapidly increasing whorls. It is corneous brown in colour on the upper side, mottled with scattered, light-brown small areas. One chestnut band is visible along the last three whorls. On the underside of the last whorl two brown bands are visible. The extent of these bands can be variable, either both of them very thin or the peripheral band very broad. Sometimes a number of thinner additional bands are present next to the main peripheral band or, less frequently, no bands are present at all on the underside of the last whorl. The peri-umbilical area is usually the lightest in colour. The spire is broadly conical, making the shell appear discoidal to tectiform. The periphery of the last whorl is either keeled, angled or rounded. The keel is often very distinct and may be slightly bend downwards. The keel is usually lighter in colour than the remaining surface of the whorls, being light brown to whitish. The external upper surface has very fine but clearly visible, relatively regularly spaced growth lines. Regularly disposed, very small, drop-like tubercles are present all over the teleoconch, especially along the growth lines. The dimensions of the tubercles remain quite stable along the whorls and also their density is approximately the same on all teleoconch whorls. The underside of the last whorl is also equipped with tubercles. The last whorl is only slightly wider than the penultimate whorl, and only slightly descending near the aperture. The umbilicus can either be completely closed or widely open (up to ^1^/_5_ of the maximum shell diameter). The aperture is elliptical. The lower palatal side of the last whorl shows a strong callous immediately behind the aperture or none. The peristome is interrupted along the palatal area, and it is only slightly reflected along the lower section. The palatal area never shows any kind callosities or thickenings.

**Body.** Very similar to *Wollastonia* gen. n. Head and neck are usually grey to dark grey. The sides and the posterior upper section of the foot are whitish to grey. The foot is whitish and the sole is longitudinally divided into three areas. The central area is smooth, whereas the two lateral portions are equipped with bands of muscles that are roughly arranged in a chevron pattern. The mantle border is dark grey, with five more or less well-developed lobes. In some specimens, one of these lobes (either lateral or dorsal) may be totally missing. The walls of the pallial cavity are colourless without any stripes or spots. A strong pulmonary vein is visible. The right ommatophoral retractor is independent from both penis and vagina.

**Genitalia.** The general arrangement of the genitalia is semi-diaulic monotrematic. A convoluted hermaphroditic duct arises from the gonad. The albumen gland is long and thin and connected to an approximately twice as long sperm-oviduct that consists of a prostatic and a uterine portion. The prostatic part extends to a thin vas deferens, which is approximately twice as long as the sperm-oviduct and which is terminating in the penial complex. The distal portion of the uterine part extends into the free oviduct, which transforms into a vagina at the level of the duct of the bursa copulatrix. The free oviduct is approximately as long as the vagina. The duct of the bursa copulatrix is very wide, approximately as long as the penial complex and usually uniform in diameter. It ends with an oval bursa copulatrix. The transition area between the duct and the bursa itself is not sharply delimited but rather gradually widens. The spermatophore is unknown. One tuft of digitiform glands arises from the proximal part of the vagina. There are usually two, equally long and very rarely branched glands present. A vaginal appendix arises from the vagina’s wall, just distal of the glandular tuft. Very smooth, rather wide, and little elevated, irregularly spaced pleats run longitudinally along the inner surface of the vagina, which reach into the genital atrium but not as far as the genital orifice. The atrium is short and wide. Its internal walls are smooth. The penial complex consists of a flagellum, an epiphallus (which extends from insertion of the vas deferens to the penial retractor muscle) and a penis that inserts into the genital atrium. The penial flagellum is short, cylindrical and with a pointed apex. It is usually ¼ as long as the epiphallus. Its internal walls are completely smooth. The epiphallus is usually ⅓ longer than the penis. Its internal walls are equipped with 20–25 very fine, elevated, longitudinal pleats. The retractor muscle is strong and approximately half as long as the epiphallus. The penis lacks any muscular or glandular sheath. It is extremely thick-walled and approximately ⅔ as long as the epiphallus. It is cylindrical and slightly swollen in its distal portion. The inner walls of the penis are smooth. The section where the rather large penial papilla is located is usually detectable from outside by virtue of a circular swelling corresponding to the origin of the papilla itself. The penial papilla is approximately half as long as the entire penis. It is conical to subcylindrical in shape and has smooth external walls, with an apically emerging opening. The channel of the penial papilla is thin and narrow. The inner lumen of the penial papilla is occupied by a spongy and sturdy tissue which directly connects with the walls of the epiphallus. The longitudinal section of the penial papilla (Fig. 226) shows that its walls are the continuation of the penial walls that abruptly bend inwards. See also Schileyko (2005).

**Jaw and radula.** The jaw and the radula of *Callina* are very similar to those of *Hystricella* and *Wollastonia*. Only the number of the lateral and marginal teeth may be slightly higher: 21 to 29. They do not differ markedly from each other, i.e. their shape gradually changes from the first laterals towards the marginals. The jaw is odontognathous and very variable in shape, from almost straight to markedly arched. There are many smooth transverse ridges, ranging from 9 to 10 in number. For jaws and radulae of *Callina* species, see Figs 205–207.

##### Distribution.

**Figure 204.**
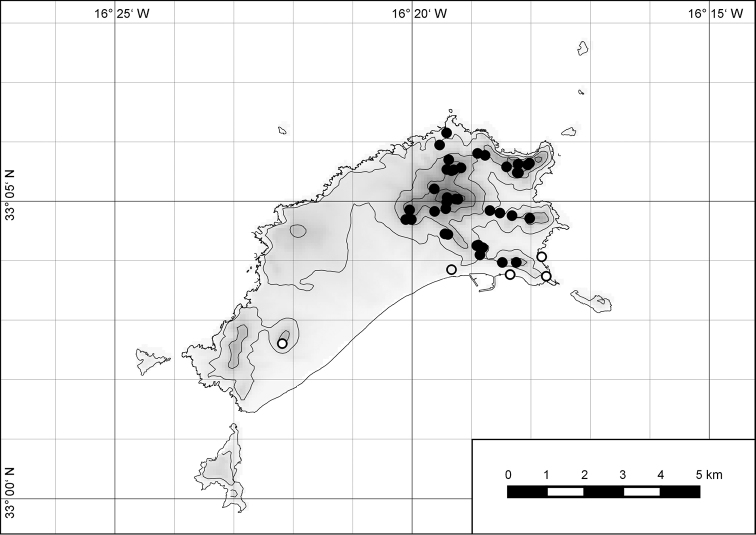
Distribution of the genus *Callina*. Filled circles refer to recent and open circles to fossil records.

The genus *Callina* is endemic to the Island of Porto Santo (Madeiran Archipelago, Portugal). It is distributed along the central-eastern area of the main island. It is not present on the offshore islets. It is only present east of the line Vila Baleira-Dragoal-Camacha-Porto de Salemas. For distributional maps of *Callina* species, see Figs 204, 214, 217 and 224.

**Figures 205–209.**
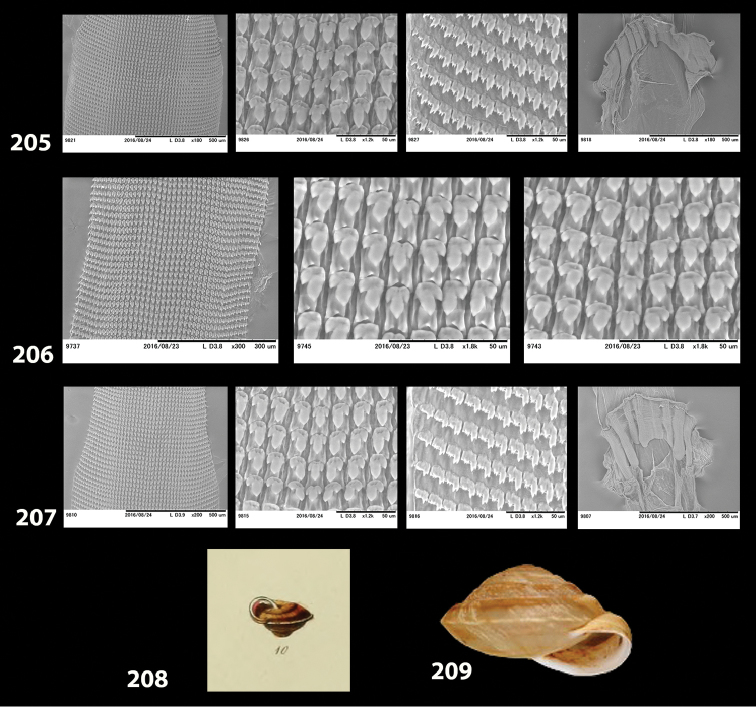
**205, 206** radula and jaw of *Callina rotula* from Ribeira da Areia **207** radula and jaw of *Callina bulverii* **208** original figure of *Helix rotula* R. T. Lowe, 1831 (from Lowe 1831: pl. 6 fig. 10) **209** lectotype of *Callina rotula*, ANSP 97116. Scale bar 1 mm.

##### Taxonomic remarks.

The results of the phylogenetic analyses indicate that *Callina* is not closely related to *Discula* R. T. Lowe, 1852 s. lat. as previously supposed but represents a well-supported clade that is closely related to *Hystricella* and *Wollastonia* gen. n. The taxon was already elevated to generic rank by Waldén (1995) and Schileyko (2005), although without providing strong morphological evidence for this decision. An isolated position of *C. rotula* within *Discula* s. lat. was supposed by these authors on the basis of a closed (or “dot-like” following Schileyko, 2005: 2018) umbilicus. However, our morphological (genitalia) and genetic investigations showed that the closed umbilicus of *C. rotula* cannot be used as a discriminating feature at the genus-level as the widely umbilicated species *C. bulverii* also belongs to *Callina* as supported by the phylogenetic analyses and the peculiar arrangement of pleats on the inner wall of the epiphallus that is equipped with 20–25 very fine, elevated longitudinal pleats (Fig. 211). Contrarily, both *Discula* s. str. and Discula (Mandahlia) have an epiphallus with 5–7 strong and rather elevated, longitudinal pleats (Figs 228–230). The penial papilla of *Callina* (Fig. 211 and Fig. 225) always reaches the genital atrium, whereas in *Discula* s. str. and Discula (Mandahlia) the penial papilla is much shorter and never reaches the genital atrium (Figs 228–229). Discula (Mandahlia) has a very long epiphallus, at least three times longer than the penis (Fig. 230).

#### Callina rotula

Taxon classificationAnimaliaStylommatophoraGeomitridae

(R. T. Lowe, 1831)

Figs 206–209 , 210–213 , 214

##### List of synonyms.

1831 *Helix rotula* R. T. Lowe: 53, pl. 6 fig. 10.

1847 *Helix rotula* – L. Pfeiffer in L. Pfeiffer 1847–1848: 216.

1854 *Helix rotula* – Reeve in Reeve 1851–1854: pl. 137 fig. 854.

1854 *Helix rotula* – Albers: 28, pl. 6 figs 16–18.

1855 Helix (Callina) rotula – R. T. Lowe: 183.

1867 Helix (Tectula) rotula – Paiva: 82.

1867 Helix (Tectula) rotula var. α major Paiva: 83.

1867 Helix (Tectula) rotula var. β minor Paiva: 83.

1867 Helix (Tectula) rotula var.γcerina Paiva: 83.

1867 Helix (Tectula) rotulavar.δmonstrosa Paiva: 83.

1878 Helix (Callina) rotula – Wollaston: 151–152.

1888 *Helix rotula* – Tryon in Tryon [and Pilsbry] 1888: 46, pl. 9 fig. 4.

1894 *Geomitra rotula* – Pilsbry in Pilsbry 1893–1895: 241.

1922 Ochthephila (Callina) rotula mut. *grisea* T. D. A. Cockerell: 44–46 [unavailable, infrasubspecific].

1931 Geomitra (Actinella) rotula – Nobre: 81, figs. 30 + 31.

1950 Discula (Callina) rotula – Mandahl-Barth: 36.

1983 Discula (Callina) rotula – Waldén: 267, note p. 273 [partim].

2002 Discula (Callina) rotula – Bank et al.: 124.

2005 *Callina rotula* – Schileyko: 218–219.

2008 Discula (Callina) rotula – Seddon: 78, pl. 28 fig. E, map 175.

2011 *Discula rotula* – Seddon: e.T156384A4936221.

##### Type material.

ANSP 97116, lectotype (herewith designated), from loc. typ., ex coll. R. T. Lowe-T. V. Wollaston. For the original figure of *Helix rotula* R. T. Lowe, 1831 (from Lowe 1831: pl. 6 fig. 10), see Fig. 206. The lectotype of *Helix rotula* R. T. Lowe, 1831, W = 11.2 mm, is depicted in Fig. 207.

##### Further material examined.

All from Porto Santo. Fossil: CKG/1 juv., SE slope of the Pico de Ana Ferreira, 33°02'36"N/16°22'11"W, 220 m, leg. K. & C. Groh & J. & C. Hemmen, Jul. 6 1983; CKG/15, Barbinha, Quaternary aeolinites, 33°04'04"N/16°17'49"W, 8 m, leg. K. & C. Groh & J. & C. Hemmen, Jul. 4 1983; CFW 11227/1, Ponta da Galé, E end of the tunnel, 33°03'44"N/16°17'45"W, 30 m, leg. F. Walther & E. M. Gryl, Apr. 1 2017. Recent: CKG/20, NW slope of Pico Juliana, along the upper Ribeira do Pedregal, 33°05'32"N/16°19'25"W, 340 m, leg. K. & C. Groh & J. & C. Hemmen, Jul. 3 1983; CKG/1, Terra Chã, path to forestry house, 33°05'37"N/16°18'03"W, 350 m, leg. K. Groh & J. Hemmen, Jul. 8 1983; CKG/2, region NW of the summit of Pico do Concelho, 33°04'43"N/16°18'01"W, 200–230 m, leg. K. Groh & J. Hemmen, Jun. 29 1983; CKG/1, SW slope of Lombo Branco, 33°05'46"N/16°18'46"W to 33°05'38"N/16°18'13"W, 150–400 m, leg. K. Groh & J. Hemmen, Jul. 8 1983; CKG/5, CMN/5, W slope and crest of the hill W of Pico do Concelho (Pico Novalido?), 33°04'50"N/16°19'37"W, 100–250 m, leg. K. Groh & J. Hemmen, Jun. 29 1983; CKG/10, N slope of Pico do Facho, 33°05'04"N/16°19'25"W, approx. 450 m, *Tamarix*- and *Pinus*-forest, leg. K. Groh & J. Hemmen, Jun. 28 1983; CMN/1, Pico de Juliana towards Cabo de Grafa, 33°05'34"N/16°19'11"W, 300 m, leg. K. & C. Groh, Aug. 13 1985; CWDM/5, Ribeira da Areia, serpentine 240 m NNW the quarry, under stones, 33°04'51"N/16°18'41"W, 120 m, leg. W. De Mattia & J. Macor, May 15 2014; CWDM/4, beginning of path to Pico Branco, 33°05'48"N/16°18'54"W, 180 m, leg. W. De Mattia & J. Macor, May 15 2015; CWDM/5, Pico de Cabrita, Pedregal de Dentro, northern slope close to the road, under stones, 33°05'42"N/16°19'23"W, 240 m, leg. W. De Mattia & J. Macor, May 15 2015; CWDM/5, path to Pico Branco, terraced S slope of Pico Branco, under stones, 33°05'35"N/16°18'25"W, 270 m, leg. W. De Mattia & J. Macor, May 16, 2015; CWDM/4, Cabeco dos Bades at the confluence of the two streams, under stones, 33°06'09"N/16°19'25"W, 30 m, leg. W. De Mattia & J. Macor, May 19 2015; CWDM/4, lake S of Ribeira da Serra de Dentro, under stones, 33°04'48"N/16°18'31"W, 70 m, leg. W. De Mattia & J. Macor, May 22 2014; CWDM/5, path to Pico Branco, eastern steep part of path to Pico Branco, under stones, 33°05'29"N/16°18'14"W, 320 m, leg. W. De Mattia & J. Macor, May 22 2014; CWDM/4, Terra Chã-Pico Branco ridge, under stones, 33°05'38"N/16°18'01"W, 335 m, leg. W. De Mattia & J. Macor, May 16 2014; ZMH 120617/2, Pico de Cabrita, 33°05'02"N/16°19'16"W, 460 m, ex coll. W. Fauer, leg. M. Vilella, Mar. 20 1964; ZMH 120616/3, Pico de Juliana, 33°05'32"N/16°19'18"W, c. 360 m, ex coll. W. Fauer, leg. J. & C. Hemmen, Jun. 29 1983; ZMH 110140/15, slopes of Pico do Facho, under stones, c. 33°04'59"N/16°19'25"W, 430 m, leg. E. Clauss, Sep. 22 1992; CFW 10997/11, ZMH 92904/4, southwestern slope of Pico Branco ca. 250 m SW of the top, 33°05'29"N/16°18'13"W, 310 m, leg. F. Walther & E. M. Gryl, Mar. 31 2017; CFW 11107/2, S slope of Pico do Castelo, near parking area, 33°04'42"N/16°20'06"W, 260 m, leg. F. Walther & E. M. Gryl, Apr. 1 2017; CFW 10889/4, ZMH 92889/1, Ribeiro do Pedregal, upper part, downstream of the abandoned houses, 33°05'59"N/16°19'32"W, 120 m, leg. F. Walther, Apr. 2 2017; CFW 10878/4, Pico do Facho, northwestern slope, 33°05'13"N/16°19'38"W, 370 m, leg. F. Walther & E. M. Gryl, Apr. 3 2017; CFW 10835/4, ZMH 92811/1, Casinhas, N of Capela da Graça, 33°04'27"N/16°19'27"W, 150 m, leg. F. Walther, Apr. 4 2017; ZMH 24259/2, Madeira Archipelago, without locality data, ex coll. Altonaer Museum; ZMH 24260/1, Porto Santo, without exact locality data, ex coll. Altonaer Museum, ex coll. O. Semper, ex coll. Dohrn.

##### Loci typici.

[*rotula*] Hab. in montibus Portus S^ti^.; not given for the var. α to δ of Paiva. [*grisea*] Porto Santo, main island.

##### Original descriptions.

[*rotula*] from Lowe, 1831: H. testa rotundata, conoideo-depressa, supra sub-planulata, subperforata, carinata, scabra, nitidiuscula, fasciata: spira conoidea, obtusissima; sutura obsoleta; anfractibus planis, transverse striatis et granulatis; ultimo acute carinato, carina ad peristoma obsoleta: aperture lunata, extrorsum dilatata; peristomate intiis incrassato, acuto, sub-expanso; ad angulum internum reflexo, calloso, perforationem obtegente. Axis 3 lin. Diam. 6. Anfr. 8; from Paiva 1867: [var. α *major*] testa distincto fasciata, ampliore; [var. β minor] testa duplo minore, fasciis angustioribus; [var. γ cerina] testa albida, omnio efasciata; [var. δ *monstrosa*] testa fasciata, anfractibus subsolutis, carina supra suturam valde prominent, rarissima; [*grisea*]; from Cockerell 1922: Shell pale gray, flecked with creamy white.

##### Redescription of shell.

The shell is dextral, solid, hairless, and it is usually discoidal to tectiform. The protoconch is dark brown with 1.5 to 1.7 whorls. It is almost smooth along the first whorl and shows radial striae along its remaining portion. The teleoconch has from 5.3 to 5.7 rapidly increasing whorls. It is more or less horn brown in colour on the upper side, mottled with small light brown scattered areas. One more or less blurred chestnut band is sometimes visible along the last three whorls. On the underside of the last whorl two brown bands are present that are usually not very broad. The peri-umbilical area is usually the lightest in colour. The spire has a rather conical shape, with the rather flat whorls being separated by a shallow suture. The last whorl is not equipped with a real keel but is rather distinctly angled. The peripheral angulation is often lighter in colour compared to the remaining shell surface, usually whitish to light brown. The surface of the shell has very fine but clearly visible, rather regularly spaced, growth lines. Small tubercles are present on surface of the teleoconch. These tubercles are concentrated along the growth lines, and especially on the underside quite dense. The last whorl is only slightly wider than the penultimate whorl and slightly descending towards the aperture. The umbilicus is completely closed. The aperture is elliptical and the peristome is white and rather solid. A strong callous along the lower palatal side is present, just behind the aperture. The peristome is interrupted along the palatal area and partially reflected along its basal side. The palatal area does not show any callouses or thickenings. See Figs 208–209.

##### Measurements.

D 11.1 ± 0.6 mm (range 10.4–11.8 mm); H 7.2 ± 0.7 mm (range 6.3–8.1 mm); FW 5.1 ± 0.2 mm (range 5.0–5.6 mm); PA 44.0 ± 2.1° (range 42.3–46.4°); NT > 100; NW 7.4 ± 0.3 (range 7.0–7.6) (*n* = 25). Ratio D/H 1.6; ratio FW/H 0.6.

##### Body.

As in the genus description.

##### Genital anatomy.

**Figures 210–213.**
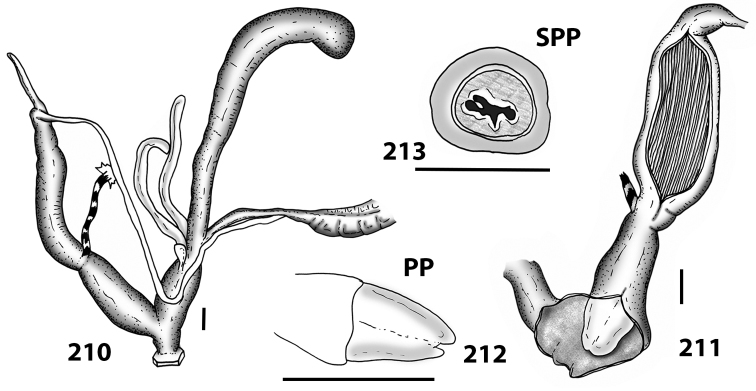
Genitalia and anatomy of *Callina rotula*. Ribeira da Areia: **210** whole genitalia excluding part of OSD, AG and gonads **211** ornamentation of the inner walls of the flagellum, the penial complex and the genital atrium **212** penial papilla **213** cross section of penial papilla. Scale bars 1 mm.

A convoluted hermaphroditic duct arises from the gonad. The albumen gland is long and thin and is connected to an approximately twice as long sperm-oviduct that consists of a prostatic and a uterine portion. The prostatic part extends into a thin vas deferens which is approximately twice as long as the sperm-oviduct and which inserts into the penial complex. The distal portion of the uterine part extends into the free oviduct, turning into a vagina at the level of the duct of the bursa copulatrix. The free oviduct is approximately as long as the vagina. The duct of the bursa copulatrix is very wide, approximately as long as the penial complex and usually uniform in diameter. It ends in an oval bursa copulatrix. The transition area between the duct and the bursa itself is not sharply delimited but rather gradually widens. The spermatophore is unknown. One tuft of digitiform glands arises from the proximal part of the vagina. There are usually two, equally long and very rarely branched glands present. A vaginal appendix arises from the vagina’s wall, just distal of the glandular tuft. Very smooth, rather widely separated, and little elevated, irregularly spaced pleats run longitudinally along the inner surface of the vagina, reaching the genital atrium but not as far as the genital orifice. The atrium is short and wide. Its internal walls are smooth. The penial complex consists of a flagellum, an epiphallus (which extends from insertion of vas deferens to penial retractor muscle) and a penis that inserts into the genital atrium. The penial flagellum is short, cylindrical and with a pointed apex. It is usually ¼ as long as the epiphallus. Its internal walls are completely smooth. The epiphallus is usually ⅓ longer than the penis. Its internal walls are equipped with 20–25 very fine, and elevated longitudinal pleats. The retractor muscle is strong and approximately half as long as the epiphallus. The penis lacks any muscular or glandular sheath. It is extremely thick-walled and approximately ⅔ as long as the epiphallus. It is cylindrical and slightly swollen in its distal part. The inner walls of the penis are smooth. The section where the large penial papilla is located is usually detectable from the outside by virtue of a circular swelling corresponding to the origin of the papilla itself. The penial papilla reaches half the length of the penis. It is conical to subcylindrical in shape and has smooth external walls. Its opening emerges apically. The channel of the penial papilla is thin and narrow. The inner lumen of the penial papilla is occupied by a spongy and sturdy tissue which directly connects with the walls of the epiphallus. The longitudinal section of the penial papilla shows that its walls are the continuation of the penial walls that abruptly bend inwards (See Schileyko (2005) and Figs 210–213).

##### Ecology.

*Callina rotula* is commonly found under volcanic rocks scattered on grassland in open fields that are more or less sloping. The specimens aestivate among the soil and are only rarely attached to the lower surface of the rocks.

##### Distribution.

**Figure 214.**
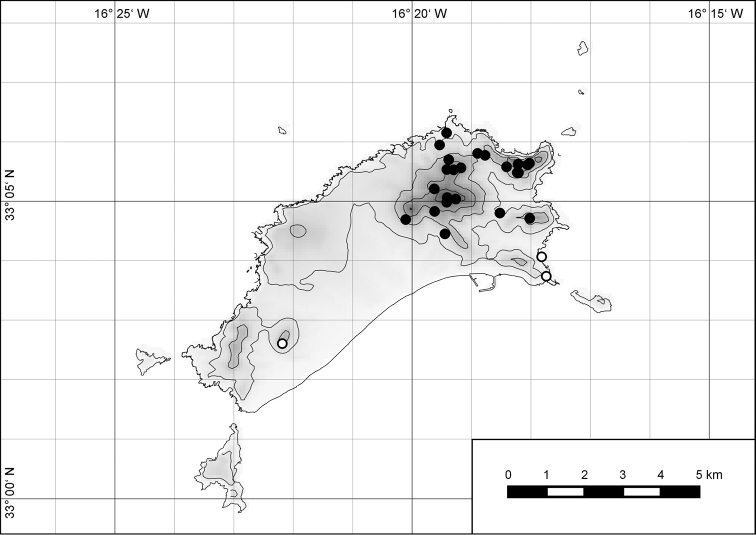
Distribution of *Callina rotula*. Filled circles refer to recent and open circles to fossil records.

*Callina rotula* is endemic to the island of Porto Santo (Madeiran Archipelago, Portugal). It is commonly found in the central-northern areas, north of the line Farrobo-Calhau da Serra de Fora. It is not present on the small islets surrounding the eastern side of the main island, namely Ilhéu de Cima and Ilhéu de Cenouras. See the distributional map in Fig. 214.

##### Taxonomic remarks.

Waldén (1983: 273) referred to a fossil specimen resembling *C. rotula* under his remark 72 from the MMF collection. After checking Waldén's specimen in the collection of the MMF and a recently collected shell, this fossil form apparently belongs to a new species that is described below.

##### Status and conservation.

According to Seddon (2011f) the species is considered Least Concern (LC).

#### † Callina waldeni

Taxon classificationAnimaliaStylommatophoraGeomitridae

Groh & De Mattia sp. n.

http://zoobank.org/033EFDF1-F522-4652-B259-57C8E313878D

Figs 215–216 , 217

##### List of synonyms.

1983 Discula (Callina) rotula – Waldén: 267, note p. 273 [partim].

##### Type material.

MMF 46276, holotype, from loc. typ.; CWDM/1 PT, Porto Santo, E of Vila Baleira, S slope of the hill above Vale do Touro, 50 m W of the oil tanks, excavated Quaternary mixed gravel, 33°03'47"N/16°19'26"W, 24 m, leg. W. De Mattia & J. Macor, May 24 2015.

##### Locus typicus.

Porto Santo, Quaternary slope deposits in Vale do Touro, Quaternary mixed gravel, 33°03'51"N/16°19'20"W, 30 m.

##### Diagnosis.

*Callina* species with closed umbilicus and well-rounded rather than angulated or keeled last whorl; granulation arranged along the growth lines and not evenly scattered across the entire shell surface.

##### Description of the holotype.

The shell is dextral, very solid, hairless, and discoidal to conical in shape. The protoconch is dark brown, with 1.3 to 1.6 whorls and almost smooth except for very fine radial striae. The teleoconch has from 6.4 to 6.7 rapidly increasing whorls. It is whitish in colour, probably bleached. The whorls are slightly convex. The last whorl is rather rounded, neither distinctly angled nor keeled. The external upper surface has very fine but clearly visible, irregularly spaced, growth lines. Small scattered tubercles are present on the upper surface of the teleoconch. These tubercles are arranged along the growth lines. Small and rather densely set (exclusively along the growth lines) tubercles are also present on the underside of the last whorl. The last whorl is only slightly wider than the penultimate whorl and only slightly descending towards the aperture. The umbilicus is closed. The aperture is more or less elliptical and the peristome is white and very solid. A strong callous is present along the lower palatal side just behind the aperture. The peristome is interrupted along the palatal area and partially reflected along its basal portion. The palatal area does not show any callouses or thickenings.

##### Measurements.

D 15.7 ± 0.1 mm (range 15.6–15.8 mm); H 9.1 ± 0.3 mm (range 8.9–9.2 mm); FW 5.6 ± 0.2 mm (range 5.5–5.7); PA 52.4 ± 2.4° (range 51.2–54.4°); NW 7.9 ± 0.3 (range 7.7–8.1) (*n* = 2). Ratio D/H 1.7; ratio FW/H 0.6.

##### Distribution.

*Callina waldeni* sp. n. is only known from the locus typicus. See map in Fig. 217.

##### Taxonomic remarks.

**Figures 215–216.**
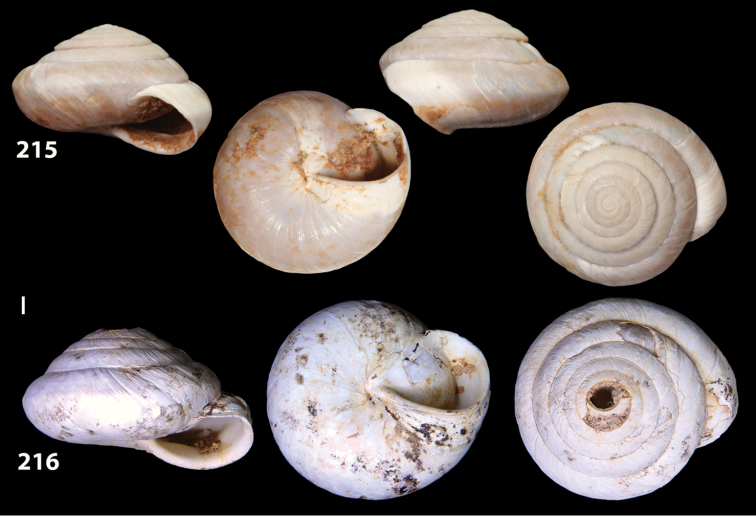
Shells of *Callina waldeni* sp. n. **215** holotype, MMF 46276 **216** paratype from E of Vila Baleira, S slope of the hill above Vale do Touro. Scale bar 1 mm.

Waldén (1983: 273) reported in note 72: “A single giant, subfossil specimen (diam. maj. = 15.8 mm, H = 8.9 mm, whorls: 7.9) has been found in Vale do Touro, Porto Santo (Voucher specimen in Funchal Museum)”. Waldén clearly introduced the fossil specimen of the MMF collection (n° 46276) as belonging to *Callina rotula* as also reported in the original museum label depicted in Fig. 215. *Callina waldeni* sp. n. differs from *C. rotula* by its larger dimensions and a more conical shape, with slightly more convex whorls. The body whorl is not distinctly angled but rather well rounded. The tubercles are only arranged along the growth lines and not scattered on the entire surface of the shell. The aperture is also less elliptical.

*Callina waldeni* sp. n. is currently the only known fossil representative of the genus.

**Figure 217.**
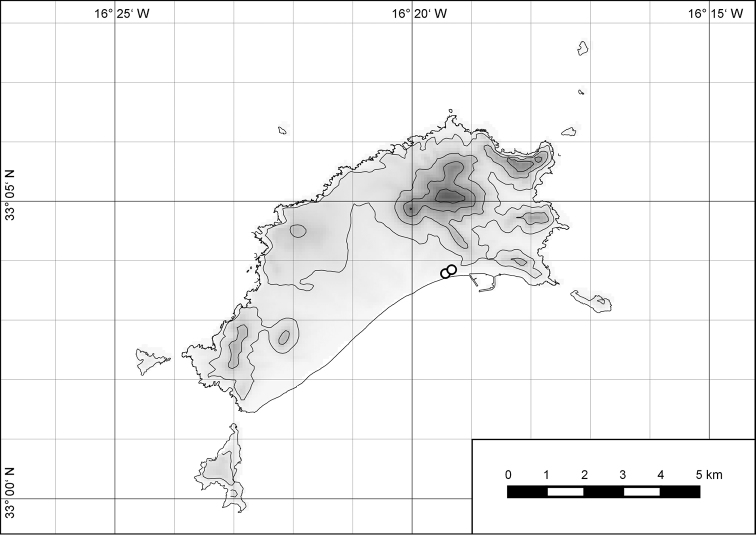
Distribution of *Callina waldeni* sp. n.

##### Status and conservation.

Extinct before the islands’ scientific exploration in the 19^th^ century, possibly already before human settlement.

#### Callina bulverii

Taxon classificationAnimaliaStylommatophoraGeomitridae

(W. Wood, 1828) comb. n.

Figs 218–219 , 220–226 , 227

##### List of synonyms.

1828 *Helix bulverii* W. Wood: 25, pl. 8 fig. 82.

1831 *HelixBulveriana* R. T. Lowe: 44–45, pl. 5 fig. 11.

1838 *Helix rota* Potiez & Michaud: 106–107.

1847 *HelixBulweriana* – L. Pfeiffer in L. Pfeiffer 1847–1848: 208.

1852 *Helix* [*Discula*] *Albersii* R. T. Lowe: 117.

1853 *Helix Bulverii* – L. Pfeiffer: 161.

1853 *Helix Bulverii* var β – L. Pfeiffer: 161.

1854 *HelixBulveriana* – Reeve in Reeve 1851–1854: pl. 136 fig. 849.

1854 *Helix* [*Tectula*] *Bulwerii* – Albers: 24, pl. 4 figs 12+13, 16–18, pl. 5 figs 1–3.

1854 *Helix* [*Tectula*] *Bulwerii* var. γ – Albers: 24, pl. 4 figs 16–18.

1854 *Helix* [*Tectula*] Bulwerii var. pallidior Albers: 91, pl. 4 figs 14–15.

1854 *Helix* [*Discula*] *Bulveriana* – R. T. Lowe: 192.

1854 *Helix* [*Discula*] *Albersii* – R. T. Lowe: 192.

1867 *Helix* [*Tectula*] *Bulweriana* – Paiva: 95.

1867 *Helix* [*Tectula*] Bulweriana var.βmajor Paiva: 95.

1888 *Helix Bulweri* – Tryon in Tryon and Pilsbry 1888: 42, pl. 9 fig. 88.

1894 *Geomitra Bulweri* – Pilsbry in Pilsbry 1893–1895: 243.

1922 Ochthephila (Tectula) bulverii mut. *albescens* T. D. A. Cockerell: 45 [unavailable, infrasubspecific].

1931 *Geomitra (Actinella) Bulweri* – Nobre: 100–102, figs. 47, 48.

1950 Discula (Discula) bulweri bulweri – Mandahl-Barth: 469: 36.

1950 Discula (Discula) bulweri albersi – Mandahl-Barth: 36, pl. 2 fig. 5, pl. 4 fig. 4, pl. 9 fig. 7.

1983 Discula (Discula) bulweri – Waldén: 20: 267.

1983 Discula (Discula) albersi – Waldén: 20: 267.

2002 Discula (Discula) bulverii – Bank et al.: 123.

2008 Discula (Discula) bulverii – Seddon: 78, pl. 29 fig. G, map 170.

2011 *Discula bulverii* – Seddon: e.T6725A12800858.

##### Type material.

**Figures 218–219.**
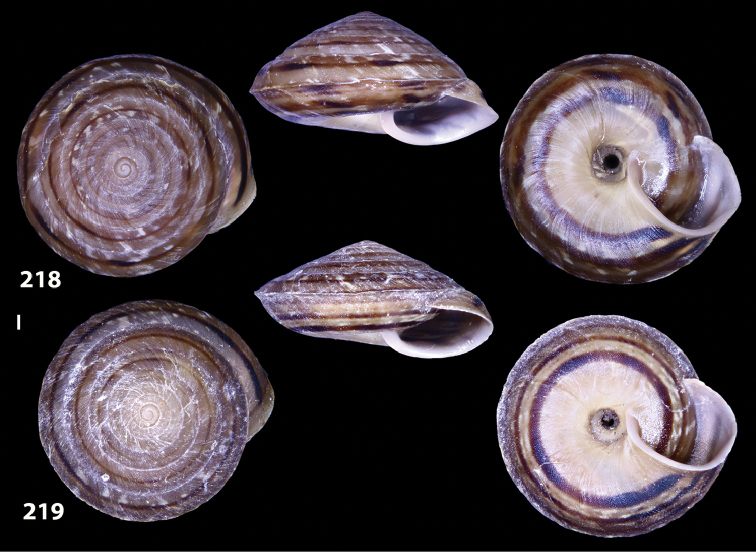
Shells of *Callina bulverii*. 218 neotype, SMF 348938 **219** specimen from Zimbreiro. Scale bar 1 mm.

Despite intensive research in multiple museum collections (NHM, NMW, MMUE, ANSP, NHC, NMS, OUMNH, RAM, SMF) that could have held the type material of the taxon, no such material could be traced and therefore we deem it reasonable to assume that the type material is lost. To stabilise the present interpretation of *Helix bulverii* W. Wood, 1828 and to clarify its taxonomic status we designate a neotype here, which is deposited in the collection of the SMF, Frankfurt a. M. under SMF 348938, W. De Mattia & J. Macor, May 15 2014. See Fig. 218. The neotype is consistent with the figure in Wood (1828: pl. 8 fig. 82) in shape, the development of the aperture and the umbilicus and colouration. The taxon was originally described from the Madeiras (= Madeira Archipelago) without more detailed locality data, which is consistent with origin from Porto Santo of the specimen selected as neotype.

##### Loci typici.

[*bulverii*] Porto Santo, S slope of Pico do Facho, along path at the S border of the pine wood, under stones, 33°04'52.47"N/16°19'25.95"W, 355 m a.s.l. (through the designation of the neotype); [*Bulveriana*] Hab. in montibus Insulae Portus S^ti.^; [*rota*] Hab. Porto-Santo (Afrique); [*Albersii*] Hab. in Portu S^to^.; [*Bulwerii*] Habitat in Portosanto, in locis siccis, aridis; [*Bulwerii* var. γ] unknown, as not mentioned; [*pallidior*] unknown, as not mentioned; [*Bulweriana*]: Habitat copiose in insulae Portosancti aridis, saxosis sub lapidibus ad montes Pico do Facho, Pico dos Maçaricos, prope N.[ossa] Senhora da Graça, Casas Velhas, Cabeço das Fontainhas, fossil ad Zimbral d’Aréa rarior; [Bulweriana var. β major]: prope N.[ossa] Senhora da Graça, rarissima; [*albescens*] Slopes of Pico do Facho, Porto Santo.

##### Further material examined.

All from Porto Santo. Fossil: ZMH 110291/5, coastal slopes at street between harbor and SE coast, 33°03'46"N/16°18'21"W, c. 10–50 m, Sep. 22 1992 and Jul. 1 1996, leg. E. Clauss; ZMH 110292/1, coastal slopes at street from harbor towards the east, 33°03'46"N/16°18'21"W, c. 10–50 m, Sep. 22 1992, leg. E. Clauss. Recent: CWDM/12, S slope of Pico do Facho, along path at the S border of the pine wood, under stones, 33°04'52"N/16°19'26"W, 355 m, leg. W. De Mattia & J. Macor, May 15 2014; CWDM/8, Pico de Juliana, under stones, 33°05'31"N/16°19'20"W, 365 m, leg. K. & C. Groh, Oct. 27 1980; CKG/10, Zimbreiro, 200 m SW of the village, near the road turn serpentine, under stones, 33°04'16"N/16°18'53"W, 90 m, leg. W. De Mattia & J. Macor, May 2014; CKG/2, summit of smaller hill and saddle below the Pico do Maçarico, 33°03'58"N/16°18'29"W, approx. 160 m, leg. K. Groh & J. Hemmen, Jul. 2 1983; CKG/2, upper Ribeira do Formosa between Covao and Serra de Fora, 33°05'02"N/16°19'14"W, 140 m, leg. K. & C. Groh & J. & C. Hemmen, Jun. 19 1983; CKG/9, bank of the road above the quarry above the Miradouro da Portela; walls and pasture above Capela da Graça towards the rocky slopes of the Pico do Facho (Motos de Fora), 33°04'42"N/16°20'01"W, 250–400 m, leg. K. & C. Groh & J. & C. Hemmen, Jun. 23 1983; ZMH 120607/4, Pico do Castelo, 33°04'42"N/16°20'01"W to 33°04'51"N/16°20'03"W, 350–430 m, leg. J. & C. Hemmen, ex coll. W. Fauer, Jul. 11 1983; ZMH 120608/2, Graça, 33°04'27"N/16°19'24"W, 190 m, leg. Balka, ex coll. W. Fauer, Jun. 25 1964; CKG/3, Pico Juliana towards Cabo de Grafa, 33°05'34"N/16°19'11"W, 300 m, leg. C. & K. Groh, Aug. 13 1985; CKG/1, Pico do Castelo, 33°04'42"N/16°20'01"W to 33°04'51"N/16°20'03"W, 350–430 m, leg. C. &. K. Groh, Aug. 13 1985; CFW 10782/<10, Casinhas, N of Capela da Graça, 33°04'27"N/16°19'29"W, 150 m, leg. F. Walther, Apr. 4 2017; CFW 10783/<10, CMN/3, ZMH 92919/>10 (tissue only), ridge between Zimbreiro and the quarry, 33°04'13"N/16°18'49"W, 110 m, leg. F. Walther, Apr. 3 2017; CFW 10847/<10, ZMH 92830/11, Casa Velhas, next to the W edge of the old quarry, 33°04'06"N/16°18'52"W, 125 m, leg. F. Walther, Apr. 3 2017; CFW 10918/7, Pico do Concelho, NW slope above water reservoir, 33°04'46"N/16°18'19"W, 170 m, leg. F. Walther, Apr. 4 2017; CFW 11126/<10, ZMH 92905/4, 200 m SW of the Zimbreiro near the road turn serpentine, 33°04'15"N/16°18'54"W, 100 m, leg. F. Walther, Mar. 31 2017; ZMH 92812/2, Pico do Maçarico, small saddle at the NW ridge, leg. 33°03'58"N/16°18'15"W, 220 m, F. Walther, Apr. 3 2017; ZMH 24261/2 [as albersi], Porto Santo, without exact locality data, ex coll. Altonaer Museum, ex coll. O. Semper, leg. Wessel; ZMH 24264/2, Porto Santo, without exact locality data, ex coll. Altonaer Museum.

##### Original descriptions.

[*bulverii*] only name and figure; [*Bulveriana*] from Lowe (1831): H. testa rotundato-depressa, hemisphaerica, rotata, supra planulata, acutissime carinata, tenui, nitidiuscula, tota minutissime et confertim granulata, fusco-castanea, supra fasciata; spira convexo-depressa, plus minus elevata, obtusissima; sutura obsoleta; anfractibus planis, acquis, quasi attritis vel confluentibus, ultimi carina acutissima, tenui, supra sulco exarata, limbata; umbilico patulo, spirali, profundo; apertura rotundato-lunata; peristomate interrupto, ad umbilicum incrassato, reflexo. Axis 3–2½ lin. Diam. 7–8. Anfr. 8–7; [*rota*] from Potiez & Michaud (1838): Cette coquille est plus petite que l’*hel. lapicida* à laquelle elle peut être comparée par sa forme générale; elle est carénée et ombiliquée comme elle, sa couleur est à peu près la même, mais elle diffère par la disposition des taches brunes; le péristome n’est ni continu, ni évasé , et l'avant-dernier tour fait une saillie dans l’ouverture. Toute la coquille est lisse et luisante à la première vue, mais à la loupe, elle est finement chagrinée et striée; la carène est très tranchante. Hab. Porto-Santo (Afrique.); [*Bulverii* var. β] from Pfeiffer (1847): Paulo minor, corneo-lutea , subtiliter marmorata et fasciata, anfractu ultimo subtus convexiore, carina minus compressa; [*Albersii*] from R. T. Lowe, 1852: Species eximia inter insigniores, in honorem cl. J. C. Albers, M. D., Helicosophi peritissimi necnon taxophilorum omnium optime meriti dicata. *H. Bulveriana* similis differt testa subminore solidiore trochiformi colore cerino-corneo magis opaco, minus (praesertim juniore) planato-depressa, subtus convexiore, carina media minus limbato-prominente, apertura ad carinam haud angulata; [*Bulwerii*] from Albers 1854: Testa umbilicata, depresse conoidea, solidula, leviter striata et eleganter dense granulate, badia, maculis pallidioribus variegate; spira parum elevate, apice obtuse; anfractus 8 regulariter accrescentes, sutura obsolete juncti, ultimus acute limbato-carinatus, subtus planulatus, saturate flavus 1-3 fasciatus, umbilicus mediocris, perspectivus; aperture oblique, elliptico-rotundata, ad carinam angulata; peristoma simplex, marginibus conniventibus, supero recto, basali crassiori, sinuato, ad umbilicus reflexiusculo. Diam. maj. 18, min. 16 ½, al. 8 ½ millim.; [Bulwerii var. γ] from Albers 1854: Testa pallida cornea, striata, sparsim granulate, anfractus ultimus subtus convexus, antice parum descendens, aperture subcircularis. Diam maj. 16, min. 15, alt. 7 millim.; [*pallidior*] from Albers 1854: only name and figure; [Bulveriana var. β major] from Paiva 1867: *β major*, spira altissima; [*albescens*] from Cockerell 1922: Shell greenish-white.

##### Redescription of the shell.

The shell is dextral, hairless, and discoidal to tectiform in shape. The protoconch is whitish to light brown with 2.1 to 2.5 whorls. It is almost smooth along the first whorl and shows fine radial striae along its remaining portion. The teleoconch has from 5.2 to 5.6 rapidly increasing whorls. It is horn brown in colour on the upper side, mottled with scattered, dark or light brown small areas. The surface of the shell is somewhat shiny. One chestnut band is present on the upper side of the teleoconch that is gradually widening and becoming darker towards the body whorl. On the underside of the last whorl one main brown band is present. Sometimes, a number of thinner additional bands are present next to the main band. The peri-umbilical area is usually the lightest in colour. The spire is broadly conical, letting the shell appear tectiform. Along the last whorl a single, distinct keel is present which may be slightly bend downwards. The keel is usually lighter in colour than the remaining surface of the whorls, being whitish to light brown. The upper surface is equipped with very fine but clearly visible, rather regularly spaced, growth lines. Small, drop-like tubercles are scattered all over the teleoconch, but concentrated along the growth lines. The last whorl is only slightly wider than the penultimate whorl and is only slightly descending towards the aperture. The umbilicus is widely open (measuring approximately 10% of the maximum shell diameter). The aperture is elliptical. The lower palatal side of the last whorl may be equipped with a strong callous just behind the aperture. The peristome is interrupted along the palatal area, and it is only slightly reflected along its basal portion. The palatal area never shows any callosities or thickenings. See Fig. 219.

##### Measurements.

D 14.2 ± 1.0 mm (range 13.3–15.6 mm); H 7.5 ± 0.3 mm (range 7.1–7.9 mm); FW 6.2 ± 0.2 mm (range 5.9–6.5); PA 48.0 ± 7.3° (range 44.6–49.4°); NT > 100; NW 7.4 ± 0.2 (range 7.2–7.6) (*n* = 30). Ratio D/H 1.9; ratio FW/H 0.8.

##### Body.

As in the genus description. *Callina bulverii* tends to have an overall slightly darker body colouration than *C. rotula*.

##### Genital anatomy.

**Figures 220–226.**
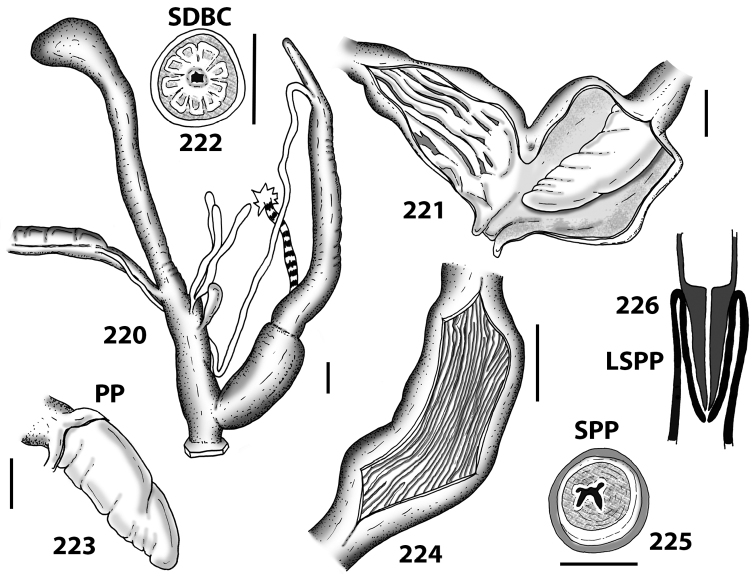
Genitalia and anatomy of *Callina bulverii*. S slope of Pico do Facho: **220** whole genitalia excluding part of OSD, AG and gonads **221** ornamentation of the inner walls of the vagina, the penis and the genital atrium **222** cross section of the duct of the bursa copulatrix **223** penial papilla **224** inner walls of epiphallus **225** cross section of penial papilla **226** longitudinal section of penial papilla. Scale bars 1 mm.

The albumen gland is long and thin and is connected to an approximately twice as long sperm-oviduct that consists of a prostatic and a uterine portion. The prostatic part extends into a thin vas deferens which is approximately twice as long as the sperm-oviduct and which inserts into the penial complex. The distal portion of the uterine part extends into the free oviduct and transforms into a vagina at the level of the duct of the bursa copulatrix. The free oviduct is ⅔ as long as the vagina. The duct of the bursa copulatrix is very wide, approximately ½ as long as the penial complex and uniform in diameter. It ends into an oval bursa copulatrix. The transition area between the duct and the bursa itself is not sharply delimited but rather gradually widens. The spermatophore is unknown. One tuft of digitiform glands arises from the proximal part of the vagina. There are two to three, approximately equally long and very rarely branched glands. A vaginal appendix arises from the vagina’s wall just distal of the glandular tuft. Irregularly spaced pleats run longitudinally along the inner surface of the vagina, reaching the genital atrium but extending not as far as the genital orifice. The atrium is short and wide. Its internal walls are smooth. The penial complex consists of a flagellum, an epiphallus, and a penis that inserts into the genital atrium. The penial flagellum is short, cylindrical and has a pointed apex. It is ⅕ as long as the epiphallus. Its internal walls are completely smooth. The epiphallus is twice as long as the penis. Its internal walls are equipped with 20–25 very fine and elevated, longitudinal pleats. The retractor muscle is strong and approximately ½ as long as the epiphallus. The penis is cylindrical, it lacks any muscular or glandular sheath, and it is extremely thick-walled. The internal walls of the penis are smooth. The section where the large penial papilla is located is usually detectable from outside by virtue of a circular swelling corresponding to the origin of the papilla itself. The penial papilla almost reaches the genital atrium and is approximately as long as the penis. It is conical to subcylindrical in shape and has smooth external walls, with the opening emerging apically. The channel of the penial papilla is thin and narrow. The inner lumen of the penial papilla is occupied by a spongy and sturdy tissue which directly connects with the walls of the epiphallus. The longitudinal section of the penial papilla (Fig. 226) shows that its walls are the continuation of the penial walls that abruptly bend inwards. See Figs 220–226.

Schileyko (2005: 2018, fig. 2549) depicted the genital anatomy of *Callina bulverii*, but, except for the penis papilla, only dealt with the outer appearance of the genitalia. Mandahl-Barth (1950) also depicted some anatomical details of *C. bulverii*, i.e. the foot pedal gland, the digitiform glands, and the vaginal appendix.

##### Jaw and radula.

The jaw has been depicted by Mandahl-Barth (1950).

##### Distribution.

**Figure 227.**
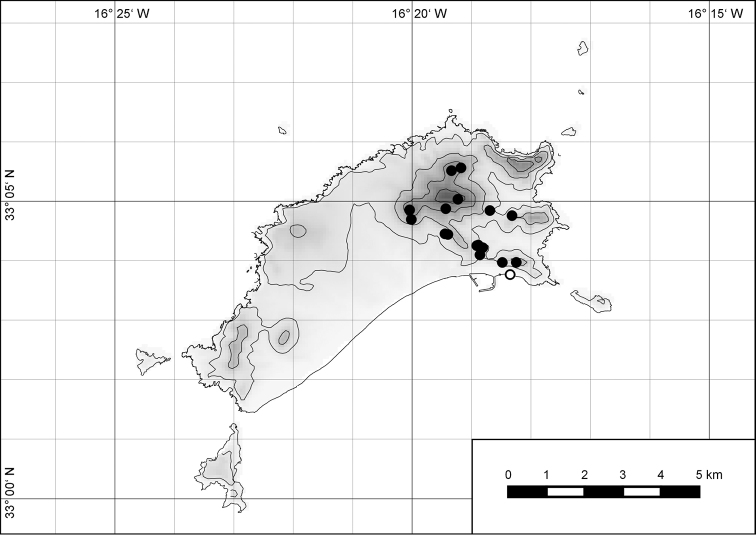
Distribution of *Callina bulverii*.

*Callina bulverii* is endemic to the island of Porto Santo (Madeiran Archipelago, Portugal). It is restricted to the area delimited by the Pico do Facho, Calhau de Serra de Dentro, Portela and Porto dos Frades. It is not present on the small islets surrounding the eastern side of the main island, namely Ilhéu de Cima and Ilhéu de Cenouras. See distribution map in Fig. 227.

##### Taxonomic remarks.

Although the shell morphology is rather different from the two other *Callina* species, the very similar genital anatomy and its position in the *cox1* tree clearly support the inclusion of the species in the genus *Callina*. Seddon (2008: 78) thoroughly summarised the nomenclatural vicissitudes of the synonyms of *C. bulverii*.

##### Status and conservation.

Seddon (2011g) assessed the species as Critical Endangered (CR B2a, b(iii, v)) but because of the wide distribution and high frequency the species should be considered Least Concern (LC).

**Figures 228–232.**
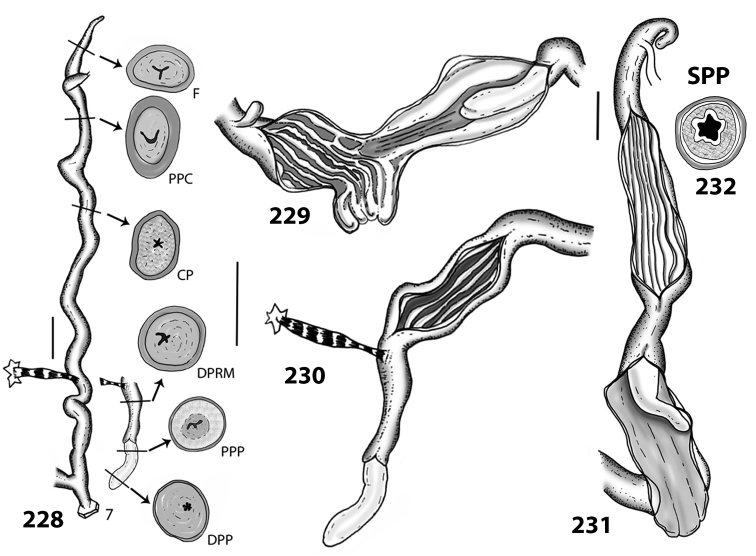
Comparison of the inner genitalia of Discula (Discula) and Discula (Mandahlia) species. **228–230** Discula (Mandahlia) tectiformis from summit slopes of Pico do Baixo: **228** cross sections of the penial complex **229** inner walls of penis, atrium and distal vagina **230** inner walls of epiphallus **231–232** Discula (Discula) polymorpha polymorpha from Porto Moniz, Madeira: **231** inner walls of epiphallus, penis, and atrium **232** cross section of penial papilla.

## Supplementary Material

XML Treatment for Hystricella

XML Treatment for Hystricella bicarinata

XML Treatment for Hystricella aucta

XML Treatment for Hystricella microcarinata

XML Treatment for Hystricella echinulata

XML Treatment for Hystricella echinoderma

XML Treatment for Wollastonia

XML Treatment for Wollastonia turricula

XML Treatment for Wollastonia vermetiformis

XML Treatment for Wollastonia ripkeni

XML Treatment for Wollastonia falknerorum

XML Treatment for Wollastonia leacockiana

XML Treatment for Wollastonia beckmanni

XML Treatment for Wollastonia jessicae jessicae

XML Treatment for Wollastonia jessicae monticola

XML Treatment for Wollastonia klausgrohi

XML Treatment for Wollastonia oxytropis

XML Treatment for Wollastonia subcarinulata

XML Treatment for Wollastonia inexpectata

XML Treatment for Callina

XML Treatment for Callina rotula

XML Treatment for Callina waldeni

XML Treatment for Callina bulverii
